# A Novel Utilization of Water Extract of Suaeda Salsa in the Pd/C Catalyzed Suzuki–Miyaura Coupling Reaction

**DOI:** 10.3390/molecules27196623

**Published:** 2022-10-06

**Authors:** Changyue Ren, Hang Zhang, Zhengjun Chen, Jie Gao, Mingyan Yang, Zeli Yuan, Xinmin Li

**Affiliations:** 1 College of Pharmacy, Zunyi Medical University, Zunyi 563003, China; 2 Key Laboratory of Basic Pharmacology of Ministry of Education and Joint International Research Laboratory of Ethnomedicine of Ministry of Education, Zunyi Medical University, Zunyi 563000, China; 3 Guizhou International Scientific and Technological Cooperation Base for Medical Photo-Theranostics Technology and Innovative Drug Development, Zunyi 563003, China

**Keywords:** Pd/C, Suzuki–Miyaura reactions, suaeda salsa, water, ligand-free

## Abstract

Using biomass-derived solvents in various organic reactions is challenging for the fine chemicals industry. We herein report a Pd/C catalyzed Suzuki–Miyaura reaction in water extract of suaeda salsa (WES) without using external phosphine ligand, base, and organic solvent. The cross-coupling reactions were carried out in a basic WES medium with a broad substrate scope and wide functional group tolerance. Furthermore, the high purity of solid biaryl products can be obtained by column chromatography or filtration.

## 1. Introduction

Green chemistry has demonstrated how fundamental scientific methodologies can protect the environment and human health in an economically safer way [1,2]. More researchers have focused on using sustainable technologies to manufacture fine chemicals and pharmaceuticals [3,4,5]. In this context, the development of greener or sustainable solvents, such as water [6], ionic liquids [7], supercritical fluids [8], and deep eutectic solvents [9], for organic reactions has attracted great attention. Recently, biomass-derived solvents as a novel green reaction medium have been successfully employed in many organic reactions, such as transition-metal-catalyzed cross-coupling reactions [10], Dakin reaction [11], Henry reaction [12], peptide synthesis [13], amides synthesis, and biodiesel synthesis [14]. Biaryl and heteroaryl are fundamental units of numerous natural products and advanced functional materials [15]. Palladium-catalyzed Suzuki–Miyaura (S–M) cross-coupling reaction has been recognized as one of the most effective methods for preparing biaryl and heteroaryl compounds [16,17,18,19]. Over the past decade, some research results demonstrated that S–M reactions could be carried out by treated plant materials and bio-wastes, which bear the features of eco-friendliness, cost-effectiveness, and safety. In 2015, Saikia and co-workers [20] reported the Pd(OAc)_2_-catalyzed S–M reaction in the presence of WEB (water extract of banana peel) at room temperature. The substrates in the reaction system showed excellent conversion under base, ligand, and organic solvent-free conditions. Subsequently, WERSA [21] (water extract of rice straw ash) and ESP [22] (eggshell powder) for the ligand-free S–M coupling reaction at room temperature were reported by Sarma group. Later, Bora and co-workers employed the water extract of waste papaya bark ash [23] and eichhornia crassipes ash [24], respectively, in the S–M reaction, which provided in situ basic conditions generated by the metal ions from plant materials ash. Suaeda salsa is a genus of plants that is non-toxic and has a wide geographical distribution around the world, so it is a type of natural feedstock. However, the application of suaeda salsa in chemistry is still not reported. In this paper, we report a Pd/C catalyzed S–M reaction in water extract of suaeda salsa ash (WES). To the best of our knowledge, this is the first report about using WES as a solvent for organic reaction.

## 2. Results and Discussion

**Figure 1:**
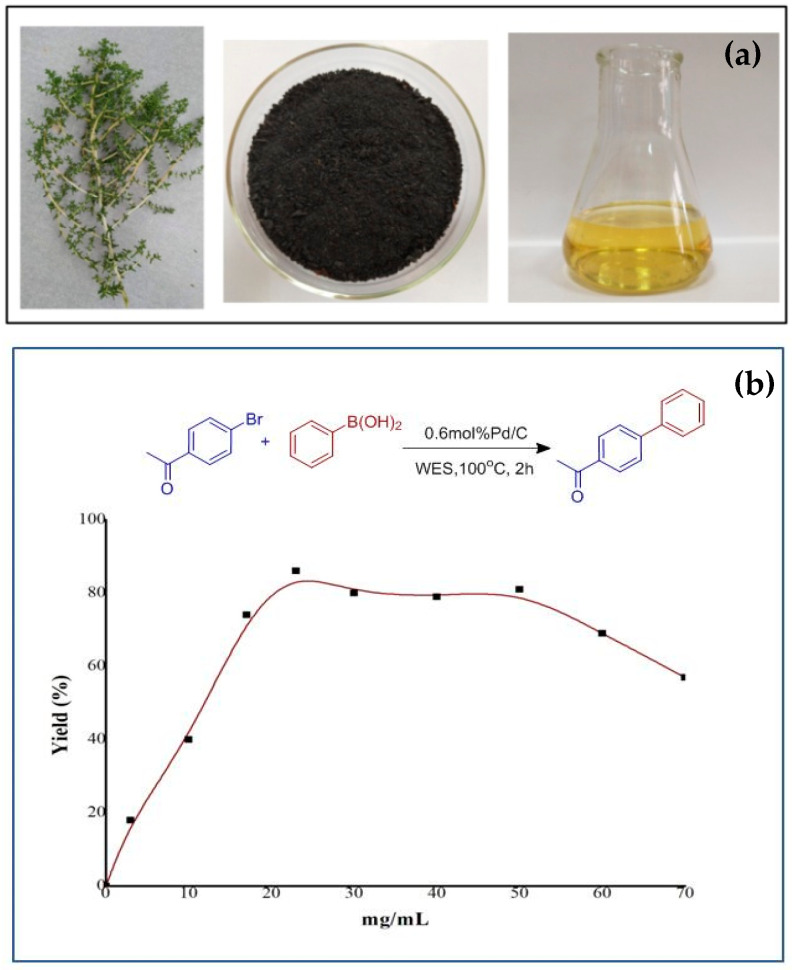
(**a**) Preparation of water extract of suaeda salsa (WES); (**b**) Optimization of WES concentration for the S–M reaction.

The water extract of suaeda salsa (WES) was prepared by burning oven-dried suaeda salsa into ashes. After that, 10 g of the ash was suspended in 100 mL distilled water and stirred for 2 h at 100 °C. The suspension was then filtered, and a light-yellow colored extract was obtained (Figure 1a). We first investigated the effect of WES concentration on the S–M reaction. Therefore, the WES solution was evaporated to light yellow solid powder which was further used to prepare a series of solutions with different concentrations. The cross-coupling of 4-bromoacetophenone with phenylboronic acid was chosen as the model reaction to explore the optimized concentration of the WES solution. As shown in Figure 1b, the S–M reaction did not proceed without the WES solution. The cross-coupling product yield increased with the increase of WES concentration. The highest product yield of 87% was obtained when the concentration of WES was 23 mg/mL. However, the product yield decreased after further increasing the concentration of WES, indicating that the concentration strongly affects the reaction. Mechanistic studies of S–M reaction indicated that bases were required in the transmetallation step for the formation of PhB(OH)^3−^ from PhB(OH)_2_, however, an excess of base could suppress the process of the S–M reaction, which is consistent with our observations [24,25,26]. We also tested the pH of each WES solution at different concentrations in the S–M reaction, which were in the range of 10.3~10.5, indicating a limited change of basicity in the WES solution. Therefore, the WES solution at a concentration of 23 mg/mL was selected as the solvent for the following study.

**Figure 2:**
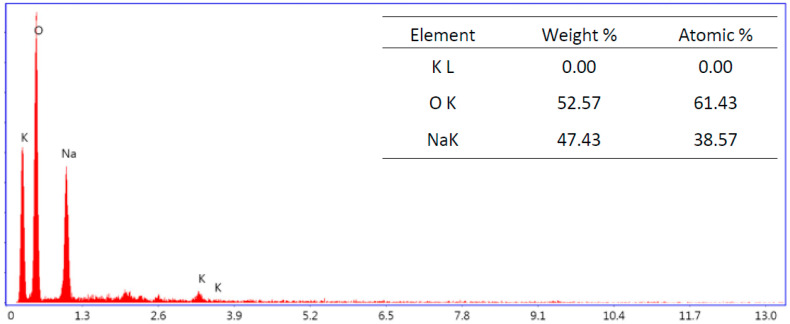
EDX spectrum of solid of WES.

To illustrate the origin of the basicity and to analyze the species and concentrations of the remaining elements, the suaeda salsa ash was characterized by energy-dispersive X-ray (EDX), revealing an abundance of the K, Na, and O (Figure 2). This suggests the presence of alkaline oxides or carbonates, which account for the basicity of extract. 

**Scheme 1:**
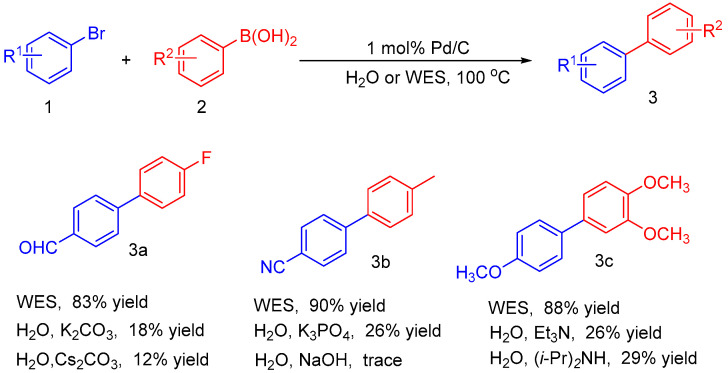
Comparison reaction between WES and other bases in water. Aryl bromide (0.5 mmol), arylboronic acid (0.525 mmol), 10 wt. % Pd/C (1 mol%), base (1 mmol), H_2_O (3 mL) or WES (3 mL), 2 h, isolated yield by column chromatography.

Several control examples were carried out to explore the differences of S–M reaction in WES media and commonly used alkaline aqueous solution system. The results were summarized in Scheme 1. The S–M reactions between different aryl halides and aryl boronic acids performed smoothly in WES, and the corresponding cross-coupling products 3a, 3b, and 3c were obtained in good yields. On the other hand, the same S–M reactions in water with inorganic or organic bases showed poor activity. This result indicates that the S–M reaction using WES was more efficient than the traditional S–M system using basic aqueous solution.

The cross-coupling reaction of 4-bromoacetophenone and phenylboronic acid in WES (23 mg/mL) was selected as a model reaction to optimize reaction conditions. The effect of the temperature was initially investigated, and a 94% yield of the cross-coupling product could be achieved at 100 °C (Table 1, entry 4). We were pleased to find that the model cross-coupling reaction still provided a 94% product yield when the loading of Pd/C decreased to 0.2 mol% (Table 1, entry 6). However, the product yield decreased significantly when the loading of Pd/C was lower than 0.2 mol% (Table 2, entry 7). Furthermore, other supported Pd catalysts were investigated, Pd/CaCO_3_ and Pd/BaSO_4_ showed almost identical catalytic activity (Table 2, entries 9 and 10), but Pd/Al_2_O_3_ gave a low product yield (Table 2, entry 8). Therefore, the optimized reaction conditions for this cross-coupling reaction are 0.2 mol% Pd/C, 3 mL WES at 100 °C.

With the optimized reaction conditions in hand, the scope and functional-group tolerance of aryl bromides and arylboronic acids were explored in this protocol. As shown in Table 2, 4-bromobenzonitrile coupled efficiently with phenylboronic acid to afford a 94% yield of 3e through silica gel column chromatography (Table 2, entry 1). It is interesting to note that the starting materials were completely reacted, and no side product of biphenyl was observed after reaction. Since the product 3e is insoluble in water, we assumed that it might be possible to obtain 3e by filtration. Therefore, we performed the same cross-coupling reaction again. The mixture was filtered through a filter funnel after reaction. The filter cake was next washed three times with water to remove the water-soluble substances. Then, the filter cake was dissolved in ethyl acetate, followed by filtration of the solution again and the ethyl acetate was concentrated in a vacuum to obtain 3e in 92% yield with high purity (Table 2, entry 1). Therefore, in the following substrate extensions, we employed filtration for separate solid biaryl products. Aryl bromides containing an electron-withdrawing group showed good reactivity in this protocol (Table 2, entries 2–5), however, 4-bromobenzoic acid provided only a 36% yield of 3i due to acidity of carboxyl group (Table 2, entry 6). The cross-coupling of 1-bromo-4-methylbenzene and phenylboronic acid afforded 3k in 13% yield (Table 2, entry 8). Aryl bromides with a group at the *para* or *meta* position were also tested, and moderate to good product yields were observed (Table 2, entries 9–13). Arylboronic acids with various groups reacted smoothly with electron-poor or electron-rich aryl bromides to provide the corresponding cross-coupling products in good yields (Table 2, entries 14–25). However, *o*-tolylboronic acid showed low activity because of the steric effect (Table 2, entries 26 and 27). Other arylboron compounds were also explored, but only potassium trifluorophenyl gave 3e in 49% yield (Table 2, entry 29). 

**Scheme 2:**
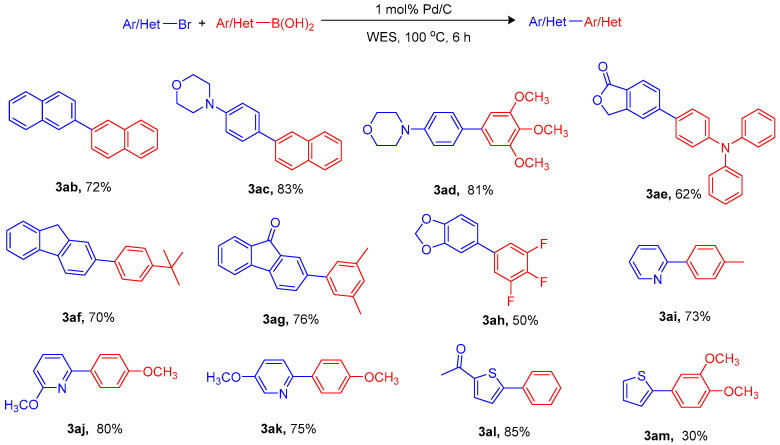
Substrate scope of Suzuki–Miyaura reaction in WES. Reaction condition: aryl/heteroaryl bromide (0.5 mmol), aryl/heteroaryl acid (0.525 mmol), 10 wt. % Pd/C (1 mol%), WES (3 mL), 100 °C, the product is obtained by column chromatography.

A variety of aryl/heteroaryl halides and boronic acids were tested to further explore the scope of the present protocol. To achieve better conversion of these substrates, we used 1 mol% Pd/C as the catalyst in the following study. As shown in Scheme 2, the cross-coupling reaction of 2-naphthaleneboronic acid with aryl bromides gave the desired products in good yield (Scheme 2, 3ab and 3ac). Arylboronic acids containing three methoxy groups provided the corresponding products 3ad in an 81% yield. 4-(Diphenylamino)phenylboronic acid coupled with 5-bromoisobenzofuran-1(3H)-one to give the corresponding product 3ae in a 62% yield. In addition, cross-coupling products of 3af and 3ag can be obtained in moderate yield. However, (3,4,5-trifluorophenyl)boronic acid coupled with 5-bromobenzo[d][1,3]dioxole to afford 3ah in only a 50% yield. Notably, this catalytic system is also tolerant of various heteroaryl bromides. The cross-coupling products 3ai-3ak can be obtained with a yield of 73–80%. 2-Acetyl-5-bromothiophene coupled with phenylboronic acid to provide 3al in an 85% yield. However, 2-brominethiophene as substrate only gave a 30% yield of 3am.

**Scheme 3:**
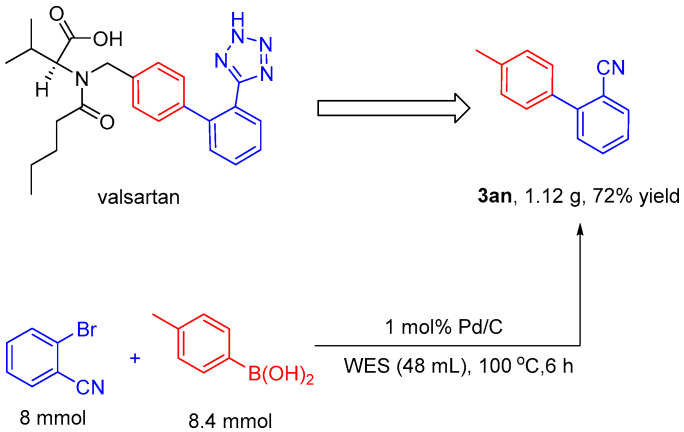
The synthesis of 2-cyano-4-methylbiphenyl by Suzuki–Miyaura cross-coupling in WES.

Valsartan (Diovan) is therapeutically useful in treating congestive heart failure and high blood pressure. 2-Cyano-4-methylbiphenyl is the core intermediate of valsartan, which can be prepared by S-M cross-coupling reaction [27]. Next, we explored the preparation of 2-cyano-4-methylbiphenyl in gram scale using WES as a reaction medium. As shown in Scheme 3, 2-bromobenzonitrile was coupled with p-tolylboronic acid in WES for 6 h in the presence of 1 mol% Pd/C and then 1.12 g 2-cyano-4-methylbiphenyl was obtained by column chromatography in a 72% yield.

In further experiments, the model reaction was carried out in gram scale to test the reusability of the Pd/C catalyst under optimized condition. After the first reaction cycle, the Pd/C catalyst was recovered by simple filtration, then washed with water and ethyl acetate, and eventually dried overnight for use in the next reaction cycle. For the first three reaction cycles, the yield of 3d dropped slightly. However, in the fifth cycle, only a 52% yield of 3d was obtained after 8 h, which was probably attributed to the loss of Pd species due to repeated filtration (Table 3, entry 5).

## 3. Experimental Section

### 3.1. General Remarks

All commercially available reagents (from Acros, Aldrich, Fluka, Energy Chemical) were used without further purification. 10 wt. % Pd/C was purchased from Energy Chemical (Palladium 10% on Carbon, ca. 50% water). All reactions were carried out under air. NMR spectra were recorded on a Brucker Advance II 400 spectrometer using TMS as internal standard (400 MHz for ^1^H NMR and ^13^C NMR). The isolated yield of products was obtained by short chromatography on a silica gel (200–300 mesh) column using petroleum ether (60–90 °C) and ethyl acetate, unless otherwise noted.

### 3.2. The Procedure for Preparing Water Extract of Suaeda Salsa

The suaeda salsa was dried overnight in an oven at 120 °C, then the dried suaeda salsa was burned to ash. After that, 10 g of the ash was suspended in 100 mL distilled water and stirred for 2 h at 100 °C. The suspension was filtered, and the light yellow colored solution was next concentrated to give a yellow solid substance, which was used to prepare suaeda salsa aqueous solution with a concentration of 23 mg/mL for Suzuki cross-coupling reactions.

### 3.3. General Procedure for the Synthesis of Biaryl and Heteroaryl Compounds

A mixture of aryl halide (0.5 mmol), arylboronic acid (0.525 mmol), 10 wt.% Pd/C (0.2–1 mol%), and WES solution (23 mg/mL, 3 mL) was stirred at 100 °C under air for the indicated time. After reaction, the mixture was cooled to room temperature. [a] The mixture was filtered and washed by water (10 mL) three times. The residue was dissolved in ethyl acetate (20 mL) and then filtered to remove the palladium carbon. Then, the ethyl acetate was collected and concentrated in vacuo to give the pure product. [b] The mixture was concentrated in vacuo and the product was isolated by short chromatography on a silica gel (200–300 mesh) column using petroleum ether and ethyl acetate.

4′-Fluoro-[1,1′-biphenyl]-4-carbaldehyde (3a), white solid. ^1^H NMR (400 MHz, CDCl_3_) *δ* 10.06 (s, 1H), 7.96 (d, *J* = 8.4 Hz, 2H), 7.72 (d, *J* = 8.2 Hz, 2H), 7.65–7.56 (m, 3H), 7.18 (t, *J* = 7.5 Hz, 2H). ^13^C NMR (101 MHz, CDCl_3_) *δ* 191.8, 146.1, 135.1, 130.3, 129.0, 128.9, 127.5, 116.0, 115.8.

4′-Methylbiphenyl-4-carbonitrile (3b), white solid. ^1^H NMR (400 MHz, CDCl_3_) *δ* 7.68 (dd, *J* = 16.1, 7.3 Hz, 4H), 7.49 (d, *J* = 7.2 Hz, 2H), 7.36–7.08 (m, 2H), 2.41 (s, 3H). ^13^C NMR (101 MHz, CDCl_3_) *δ* 145.5, 138.7, 136.2, 132.5, 129.8, 127.4, 127.0, 119.0, 110.5, 21.1.

3,4,4′-Trimethoxy-1,1′-bipheny (3c). ^1^H NMR (400 MHz, CDCl_3_) *δ* 7.49 (d, *J* = 8.3 Hz, 2H), 7.17–7.02 (m, 2H), 6.95 (dd, *J* = 14.4, 8.3 Hz, 3H), 3.94 (s, 3H), 3.92 (s, 3H), 3.85 (s, 3H). ^13^C NMR (101 MHz, CDCl_3_) *δ* 158.8, 149.0, 148.1, 133.9, 133.6, 127.8, 118.9, 114.1, 111.4, 110.1, 55.9, 55.9, 55.3.

4-Acetylbiphenyl (3d), white solid. ^1^H NMR (400 MHz, CDCl_3_) *δ* 8.04 (d, *J* = 8.6 Hz, 2H), 7.69 (d, *J* = 8.6 Hz, 2H), 7.63 (d, *J* = 7.6 Hz, 2H), 7.48 (t, *J* = 7.4 Hz, 2H), 7.40 (t, *J* = 7.3 Hz, 1H), 2.64 (s, 3H). ^13^C NMR (101 MHz, CDCl_3_) *δ* 197.9, 145.9, 139.9, 135.9, 129.0, 129.0, 128.3, 127.4, 127.3, 26.8.

4-Carbonitrilebiphenyl (3e)*,* white solid. ^1^H NMR (400 MHz, CDCl_3_) *δ* 7.73 (d, *J* = 7.7 Hz, 2H), 7.69 (d, *J* = 8.5 Hz, 2H), 7.59 (d, *J* = 8.1 Hz, 1H), 7.49 (t, *J* = 7.4 Hz, 2H), 7.46–7.39 (m, 1H). ^13^C NMR (101 MHz, CDCl_3_) *δ* 145.8, 139.3, 132.7, 129.2, 128.7, 127.87, 127.3, 119.0, 111.0.

4-Nitrobiphenyl (3f)*,* yellow solid. ^1^H NMR (400 MHz, CDCl_3_) *δ* 8.30 (d, *J* = 8.9 Hz, 2H), 7.74 (d, *J* = 9.0 Hz, 2H), 7.63 (d, *J* = 7.6 Hz, 2H), 7.58–7.36 (m, 3H). ^13^C NMR (101 MHz, CDCl_3_) *δ* 147.7, 138.9, 129.3, 129.0, 127.9, 127.5, 124.2.

4-Carbaldehydebiphenyl (3g), white solid. ^1^H NMR (400 MHz, CDCl_3_) *δ* 10.06 (s, 1H), 7.96 (d, *J* = 7.5 Hz, 2H), 7.76 (d, *J* = 7.7 Hz, 2H), 7.64 (d, *J* = 7.1 Hz, 2H), 7.49 (t, *J* = 7.1 Hz, 2H), 7.43 (d, *J* = 6.6 Hz, 1H). ^13^C NMR (101 MHz, CDCl_3_) δ 191.9, 147.18 139.7, 135.1, 130.2, 129.0, 128.4, 127.6, 127.3.

[1,1′-biphenyl]-4-ol (3h), white solid. ^1^H NMR (400 MHz, CDCl_3_) *δ* 7.55 (d, *J* = 7.1 Hz, 2H), 7.49 (d, *J* = 8.7 Hz, 2H), 7.42 (t, *J* = 7.6 Hz, 2H), 7.31 (t, *J* = 7.3 Hz, 1H), 6.91 (d, *J* = 8.7 Hz, 2H), 4.88 (s, 1H). ^13^C NMR (101 MHz, CDCl_3_) *δ* 155.1, 140.8, 134.1, 128.8, 128.5, 126.8, 115.7.

[1,1′-biphenyl]-4-carboxylic acid (3i), white solid. ^1^H NMR (400 MHz, DMSO) *δ* 13.02 (s, 1H), 8.02 (d, *J* = 7.9 Hz, 2H), 7.80 (d, *J* = 7.7 Hz, 2H), 7.74 (d, *J* = 7.1 Hz, 2H), 7.50 (t, *J* = 7.1 Hz, 2H), 7.46–7.34 (m, 1H). ^13^C NMR (101 MHz, DMSO) *δ* 167.5, 144.7, 139.4, 130.3, 130.0, 129.5, 128.7, 127.3, 127.2.

4-Methoxy-1,1′-biphenyl (3j), white solid. ^1^H NMR (400 MHz, CDCl_3_) *δ* 7.56 (t, *J* = 8.2 Hz, 4H), 7.43 (t, *J* = 7.6 Hz, 2H), 7.32 (t, *J* = 7.4 Hz, 1H), 7.00 (d, *J* = 8.5 Hz, 2H), 3.87 (s, 3H). ^13^C NMR (101 MHz, CDCl_3_) *δ* 159.2, 140.9, 133.9, 128.8, 128.2, 126.8, 126.7, 114.3, 55.4.

4-Methyl-1,1′-biphenyl (3k), colorless liquid. ^1^H NMR (400 MHz, CDCl_3_) *δ* 7.57 (d, *J* = 7.6 Hz, 2H), 7.49 (d, *J* = 7.9 Hz, 2H), 7.42 (t, *J* = 7.2 Hz, 2H), 7.31 (t, *J* = 6.8 Hz, 1H), 7.24 (d, *J* = 7.4 Hz, 2H), 2.39 (s, 3H). ^13^C NMR (101 MHz, CDCl_3_) *δ* 141.1, 138.3, 137.0, 129.4, 128.7, 127.1, 126.9, 126.9, 21.11.

Biphenyl-3-carbonitrile (3l), colorless liquid. ^1^H NMR (400 MHz, CDCl_3_) *δ* 7.85 (s, 1H), 7.80 (d, *J* = 7.8 Hz, 1H), 7.62 (d, *J* = 7.7 Hz, 1H), 7.54 (dt, *J* = 10.0, 4.9 Hz, 3H), 7.47 (t, *J* = 7.6 Hz, 2H), 7.41 (t, *J* = 7.4 Hz, 1H). ^13^C NMR (101 MHz, CDCl_3_) *δ* 142.5, 138.9, 131.5, 130.7, 129.6, 129.2, 128.4, 127.1, 118.9, 113.0.

3-Nitro-1,1′-biphenyl (3m), white solid. ^1^H NMR (400 MHz, CDCl_3_) *δ* 8.46 (s, 1H), 8.20 (d, *J* = 9.4 Hz, 1H), 7.92 (d, *J* = 7.7 Hz, 1H), 7.64–7.59 (m, 3H), 7.50 (t, *J* = 7.4 Hz, 2H), 7.43 (t, *J* = 7.2 Hz, 1H), 7.66–7.58 (m, 3H). ^13^C NMR (101 MHz, CDCl_3_) *δ* 142.8, 138.6, 133.0, 129.7, 129.1, 128.5, 127.1, 122.0, 121.9.

3-Methoxy-1,1′-biphenyl (3n), white solid. ^1^H NMR (400 MHz, CDCl_3_) *δ* 7.65 (d, *J* = 8.1 Hz, 2H), 7.48 (t, *J* = 7.7 Hz, 2H), 7.45–7.34 (m, 2H), 7.24 (d, *J* = 6.8 Hz, 1H), 7.19 (s, 1H), 6.95 (d, *J* = 8.2 Hz, 0H), 3.89 (s, 3H). ^13^C NMR (101 MHz, CDCl_3_) *δ* 159.9, 142.8, 141.1, 129.8, 128.8, 127.4, 127.2, 119.7, 112.9, 112.7, 55.3.

2-Carbonitrilebiphenyl (3o), colorless liquid. ^1^H NMR (400 MHz, CDCl_3_) *δ* 7.77 (d, *J* = 7.5 Hz, 1H), 7.65 (t, *J* = 7.5 Hz, 1H), 7.57 (d, *J* = 7.2 Hz, 2H), 7.55–7.37 (m, 5H). ^13^C NMR (101 MHz, CDCl_3_) *δ* 145.4, 138.1, 133.7, 132.8, 130.0, 128.7, 128.7, 127.5, 118.7, 111.2.

2-Methoxy-1,1′-biphenyl (3p), colourless liquid. ^1^H NMR (400 MHz, CDCl_3_) *δ* 7.52 (d, *J* = 7.3 Hz, 2H), 7.38 (t, *J* = 7.1 Hz, 2H), 7.29 (t, *J* = 8.1 Hz, 3H), 7.01 (t, *J* = 7.3 Hz, 1H), 6.95 (d, *J* = 8.1 Hz, 1H), 3.76 (s, 3H). ^13^C NMR (101 MHz, CDCl_3_) *δ*156.46, 138.54, 130.88, 130.73, 129.53, 128.60, 127.96, 126.90, 120.82, 111.24, 77.34, 77.02, 76.70, 55.55.

4′-Fluoro-[1,1′-biphenyl]-4-carbonitrile (3q), white solid. ^1^H NMR (400 MHz, CDCl_3_) *δ* 7.73 (d, *J* = 8.1 Hz, 2H), 7.64 (d, *J* = 8.1 Hz, 2H), 7.56 (dd, *J* = 8.0, 5.6 Hz, 2H), 7.18 (t, *J* = 8.5 Hz, 2H). ^13^C NMR (101 MHz, cdcl_3_) δ 164.4, 161.9, 144.6, 135.3, 135.2, 132.6, 128.9, 128.9, 127.5, 118.8, 116.2, 116.0, 110.9.

4′-Methoxybiphenyl-4-carbonitrile (3r), white solid. ^1^H NMR (400 MHz, CDCl_3_) *δ* 7.69 (d, *J* = 6.9 Hz, 2H), 7.64 (d, *J* = 7.9 Hz, 2H), 7.54 (d, *J* = 7.0 Hz, 2H), 7.01 (d, *J* = 7.9 Hz, 2H), 3.87 (s, 3H). ^13^C NMR (101 MHz, CDCl_3_) *δ* 160.1, 145.2, 132.5, 131.4, 128.3, 127.1, 119.0, 114.5, 110.0, 55.4.

3′,4′-Dimethoxy-[1,1′-biphenyl]-4-carbonitrile (3s), white solid. ^1^H NMR (400 MHz, CDCl_3_) *δ* 7.67 (dd, *J* = 20.1, 7.6 Hz, 4H), 7.17 (d, *J* = 8.2 Hz, 1H), 7.09 (s, 1H), 6.97 (d, *J* = 8.2 Hz, 1H), 3.95 (d, *J* = 8.2 Hz, 6H). ^13^C NMR (101 MHz, CDCl_3_) *δ*149.74, 149.42, 145.42, 132.54, 131.94, 127.27, 119.84, 119.02, 111.57, 110.22, 77.33, 77.01, 76.69, 56.01.

4′-Methyl-[1,1′-biphenyl]-4-carbaldehyde (3t), white solid. ^1^H NMR (400 MHz, CDCl_3_) *δ* 10.05 (s, 1H), 7.94 (d, *J* = 8.0 Hz, 2H), 7.74 (d, *J* = 8.1 Hz, 2H), 7.55 (d, *J* = 7.9 Hz, 2H), 7.29 (d, *J* = 7.8 Hz, 2H), 2.42 (s, 3H). ^13^C NMR (101 MHz, CDCl_3_) *δ* 191.9, 147.1, 138.5, 136.7, 134.9, 130.2, 129.7, 127.3, 127.1, 21.1.

4′-Methoxy-[1,1′-biphenyl]-4-carbaldehyde (3u), white solid. ^1^H NMR (400 MHz, CDCl_3_) *δ* 10.03 (s, 1H), 7.92 (d, *J* = 7.6 Hz, 2H), 7.71 (d, *J* = 7.6 Hz, 2H), 7.59 (d, *J* = 7.9 Hz, 2H), 7.01 (d, *J* = 8.0 Hz, 2H), 3.87 (s, 3H). ^13^C NMR (101 MHz, CDCl_3_) *δ* 191.8, 160.0, 146.7, 134.6, 132.0, 130.3, 128.4, 127.0, 114.4, 55.3.

4-Methoxy-4′-methyl-1,1′-biphenyl (3v), white solid. ^1^H NMR (400 MHz, CDCl_3_) *δ* 7.51 (d, *J* = 8.6 Hz, 2H), 7.44 (d, *J* = 7.7 Hz, 2H), 7.22 (d, *J* = 7.5 Hz, 2H), 6.96 (d, *J* = 8.4 Hz, 2H), 3.84 (s, 3H), 2.38 (s, 3H). ^13^C NMR (101 MHz, CDCl_3_) *δ* 158.9, 137.9, 136.3, 133.7, 129.4, 127.9, 126.5, 114.1, 55.3, 21.0.

4,4′-Dimethoxy-1,1′-biphenyl (3w), white solid. ^1^H NMR (400 MHz, CDCl_3_) *δ* 7.48 (d, *J* = 8.8 Hz, 4H), 6.96 (d, *J* = 8.8 Hz, 4H), 3.84 (s, 6H). ^13^C NMR (101 MHz, CDCl_3_) *δ* 158.6, 133.4, 127.7, 114.1, 55.3.

4-Fluoro-4′-methoxy-1,1′-biphenyl (3x), white solid. ^1^H NMR (400 MHz, CDCl_3_) *δ* 7.62–7.35 (m, 2H), 7.10 (t, *J* = 8.6 Hz, 2H), 6.97 (d, *J* = 8.6 Hz, 2H), 3.85 (s, 2H). ^13^C NMR (101 MHz, CDCl_3_) *δ* 163.2, 160.8, 159.0, 136.9, 136.9, 132.8, 128.2, 128.1, 128.0, 115.6, 115.4, 114.2, 55.3.

4-Chloro-4′-methoxy-1,1′-biphenyl (3y), brown solid. ^1^H NMR (400 MHz, cdcl_3_) δ 7.51–7.44 (m, 4H), 7.38 (d, *J* = 8.4 Hz, 2H), 6.98 (d, *J* = 8.6 Hz, 2H), 3.85 (s, 3H). ^13^C NMR (101 MHz, cdcl_3_) δ 159.3, 139.2, 132.6, 132.4, 128.8, 127.9, 127.9, 114.2, 55.3.

2-Methoxy-2′-methylbiphenyl (3z), colorless oil. ^1^H NMR (400 MHz, CDCl_3_) *δ* 7.34 (d, *J* = 5.0 Hz, 1H), 7.29–7.09 (m, 5H), 7.06–6.91 (m, 2H), 3.76 (s, 3H), 2.14 (s, 3H). ^13^C NMR (101 MHz, CDCl_3_) *δ* 156.5, 138.5, 136.8, 130.9, 130.7, 129.9, 129.5, 128.5, 127.2, 125.4, 120.4, 110.5, 55.3, 19.9.

2′-Methyl-[1,1′-biphenyl]-2-carbonitrile (3aa), colorless liquid. ^1^H NMR (400 MHz, CDCl_3_) *δ* 7.73 (d, *J* = 7.6 Hz, 1H), 7.61 (t, *J* = 7.5 Hz, 1H), 7.44 (t, *J* = 7.6 Hz, 1H), 7.39–7.24 (m, 4H), 7.21 (t, *J* = 8.9 Hz, 1H), 2.19 (s, 3H). ^13^C NMR (101 MHz, CDCl_3_) *δ* 145.8, 138.0, 135.6, 132.8, 132.5, 130.4, 130.4, 129.4, 128.7, 127.5, 125.8, 118.1, 112.8, 19.9.

2,2′-Binaphthalene (3ab), white solid. ^1^H NMR (400 MHz, CDCl_3_) *δ* 8.16 (s, 2H), 7.94 (dd, *J* = 10.5, 8.2 Hz, 4H), 7.90–7.83 (m, 4H), 7.51 (ddd, *J* = 7.3, 6.0, 3.4 Hz, 2H). ^13^C NMR (101 MHz, CDCl_3_) *δ* 138.3, 133.7, 132.6, 128.4, 128.2, 127.6, 126.3, 126.0, 125.9, 125.7.

4-(4-(Naphthalen-2-yl)phenyl)morpholine (3ac), white solid. ^1^H NMR (400 MHz, CDCl_3_) *δ* 8.01 (s, 1H), 7.94–7.81 (m, 3H), 7.74 (dd, *J* = 8.6, 1.7 Hz, 1H), 7.67 (d, *J* = 8.6 Hz, 2H), 7.56–7.42 (m, *J* = 6.9, 1.2 Hz, 2H), 7.02 (d, *J* = 8.7 Hz, 2H), 3.94–3.85 (m, 4H), 3.26–3.18 (m, 4H). ^13^C NMR (101 MHz, CDCl_3_) *δ* 137.9, 133.7, 132.3, 128.3, 128.1, 128.0, 127.6, 126.2, 125.6, 125.2, 124.7, 116.1, 66.7, 49.4.

4-(3′,4′,5′-Trimethoxy-[1,1′-biphenyl]-4-yl)morpholine (3ad), white solid. ^1^H NMR (400 MHz, CDCl_3_) *δ* 7.54–7.41 (m, 2H), 6.97 (d, *J* = 8.5 Hz, 2H), 6.72 (s, 2H), 3.93–3.85 (m, 13H), 3.23–3.14 (m, 4H). ^1^H NMR (400 MHz, CDCl_3_) *δ* 153.3, 137.5, 137.0, 136.8, 127.7, 115.7, 104.6, 103.8, 92.8, 66.7, 60.9, 56.1, 49.2.

5-(4-(Diphenylamino)phenyl)isobenzofuran-1(3H)-one (3ae), white solid. ^1^H NMR (400 MHz, CDCl_3_) *δ* 7.93 (d, *J* = 8.0 Hz, 1H), 7.70 (d, *J* = 8.1 Hz, 1H), 7.62 (s, 1H), 7.51–7.44 (m, 2H), 7.28 (tt, *J* = 3.7, 1.9 Hz, 4H), 7.17–7.10 (m, 6H), 7.10–7.03 (m, 2H), 5.34 (s, 2H). ^13^C NMR (101 MHz, CDCl_3_) *δ* 148.5, 147.4, 147.2, 146.8, 132.6, 129.4, 128.1, 127.7, 126.0, 124.9, 123.7.

2-(4-(Tert-butyl)phenyl)-9H-fluorene (3af), white solid. ^1^H NMR (400 MHz, CDCl_3_) *δ* 7.83 (dd, *J* = 15.7, 7.7 Hz, 3H), 7.64 (d, *J* = 7.9 Hz, 3H), 7.58 (d, *J* = 7.2 Hz, 1H), 7.52 (d, *J* = 7.9 Hz, 2H), 7.42 (t, *J* = 7.2 Hz, 1H), 7.34 (t, *J* = 7.1 Hz, 1H), 3.97 (s, 2H), 1.42 (s, 9H). ^13^C NMR (101 MHz, CDCl_3_) *δ* 150.1, 143.8, 143.4, 141.4, 140.6, 139.7, 138.5, 126.8, 126.6, 125.8, 125.7, 125.0, 123.6, 120.0, 119.9, 37.0, 34.5, 31.4.

2-(3,5-Dimethylphenyl)-9H-fluoren-9-one (3ag), white solid. ^1^H NMR (400 MHz, CDCl_3_) *δ* 7.85 (d, *J* = 1.2 Hz, 1H), 7.70–7.61 (m, 2H), 7.53–7.43 (m, 3H), 7.27 (dd, *J* = 7.1, 1.5 Hz, 1H), 7.21 (s, 3H), 7.00 (s, 1H), 2.37 (s, 6H). ^13^C NMR (101 MHz, CDCl_3_) *δ* 193.9, 144.3, 142.9, 142.4, 139.6, 138.4, 134.7, 134.4, 133.0, 129.5, 128.8, 124.6, 124.3, 122.9, 120.5, 120.3, 21.38.

5-(3,4,5-Trifluorophenyl)benzo[d][1,3]dioxole (3ah), white solid. ^1^H NMR (400 MHz, CDCl_3_) *δ* 7.13–7.00 (m, 2H), 6.98–6.89 (m, 2H), 6.85 (dd, *J* = 7.3, 1.3 Hz, 1H), 6.00 (s, 2H). ^13^C NMR (101 MHz, CDCl_3_) *δ* 152.6, 150.1, 148.3, 147.9, 140.1, 137.6, 137.0, 132.4, 120.5, 110.7, 108.7, 107.2, 101.4.

2-(P-tolyl)pyridine (3ai), yellow oil. ^1^H NMR (400 MHz, CDCl_3_) *δ* 8.68 (d, J = 5.3 Hz, 1H), 7.90 (d, *J* = 8.0 Hz, 2H), 7.78–7.64 (m, 2H), 7.28 (t, *J* = 7.2 Hz, 2H), 7.24–7.14 (m, 1H), 2.41 (s, 3H). ^13^C NMR (101 MHz, CDCl_3_) *δ* 157.4, 149.5, 138.9, 136.6, 136.5, 129.4, 126.7, 121.7, 120.2, 21.2.

2-Methoxy-6-(4-Methoxyphenyl)pyridine (3aj), white solid. ^1^H NMR (400 MHz, CDCl_3_) *δ* 8.01 (d, *J* = 8.6 Hz, 2H), 7.59 (t, *J* = 7.8 Hz, 1H), 7.27 (d, *J* = 8.0 Hz, 2H), 6.97 (t, J = 9.0 Hz, 2H), 6.63 (d, *J* = 8.2 Hz, 1H), 4.03 (s, 3H), 3.86 (s, 3H). ^13^C NMR (101 MHz, CDCl_3_) *δ* 163.6, 160.3, 154.4, 139.0, 131.7, 127.9, 113.9, 111.9, 108.2, 55.3, 53.1.

5-Methoxy-2-(4-Methoxyphenyl)pyridine (3ak), white solid. ^1^H NMR (400 MHz, CDCl_3_) *δ* 8.34 (s, 1H), 7.74 (dd, *J* = 8.5, 2.0 Hz, 1H), 7.45 (d, *J* = 8.5 Hz, 2H), 6.98 (d, *J* = 8.5 Hz, 2H), 6.80 (d, *J* = 8.6 Hz, 1H), 3.97 (s, 3H), 3.85 (s, 3H). ^13^C NMR (101 MHz, CDCl_3_) *δ* 163.1, 159.1, 144.4, 137.1, 130.4, 129.7, 127.7, 114.4, 110.6, 55.3, 53.4.

5-Acetyl-2-phenylthiophene (3al), yellow solid. ^1^H NMR (400 MHz, CDCl_3_) δ 7.70–7.62 (m, 3H), 7.40 (dt, *J* = 13.9, 7.0 Hz, 3H), 7.32 (d, *J* = 3.9 Hz, 1H), 2.57 (s, 3H). ^13^C NMR (101 MHz, CDCl_3_) *δ* 190.5, 152.7, 143.1, 133.4, 129.1, 126.2, 123.8, 26.5.

2-(3,4-Dimethoxyphenyl)thiophene (3am), yellow oil. ^1^H NMR (400 MHz, CDCl_3_) *δ* 7.25–7.14 (m, 3H), 7.11 (s, 1H), 7.08–7.02 (m, 1H), 6.88 (d, *J* = 8.3 Hz, 1H), 3.94 (s, 3H), 3.91 (s, 3H). ^13^C NMR (101 MHz, CDCl_3_) *δ* 149.1, 148.7, 144.4, 127.8, 127.5, 123.9, 122.3, 118.5, 111.4, 109.5, 55.9.

4′-Methyl-[1,1′-biphenyl]-2-carbonitrile (3an), white solid, 105–106 °C. ^1^H NMR (400 MHz, CDCl_3_) *δ* 7.69 (q, *J* = 8.3 Hz, 4H), 7.49 (d, *J* = 8.1 Hz, 2H), 7.34–7.27 (m, 2H), 2.41 (s, 3H). ^13^C NMR (101 MHz, CDCl_3_) *δ* 145.5, 138.7, 136.2, 132.5, 129.8, 127.4, 127.0, 119.0, 110.5, 21.1.

## 4. Conclusions

In conclusion, we have developed a simple, efficient, and biomass-based Suzuki–Miyuara reaction system without using organic solvents, promoters, and ligands. A wide range of aryl/heteroaryl bromides coupled with arylboronic acids to provide the corresponding products in good yields. In addition, most solid cross-coupling products can be easily isolated by filtration. To the best of our knowledge, suaeda salsa has not been reported as a reaction media in organic reactions. We expect the suaeda salsa will demonstrate more potential in organic chemistry in the future.

## Figures and Tables

**Table 1:**
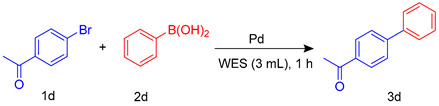
Optimization of the Suzuki–Miyaura reaction conditions

Entry	Pd Catalyst	Loading (mol%)	Temperature (°C)	Yield (%)
1	Pd/C	1	25	trace
2	Pd/C	1	50	17
3	Pd/C	1	80	71
4	Pd/C	1	100	94
5	Pd/C	0.5	100	95
6	Pd/C	0.2	100	94
7	Pd/C	0.1	100	48
8	Pd/Al_2_O_3_	0.2	100	16
9	Pd/CaCO_3_	0.2	100	91
10	Pd/BaSO_4_	0.2	100	92

Reaction condition: 4-bromoacetophenone (0.5 mmol), phenylboronic acid (0.525 mmol), catalyst, WES (3 mL), under air, 1 h, isolated yield by column chromatography.

**Table 2:**
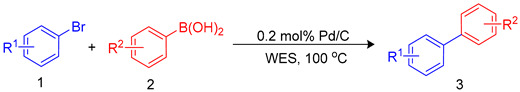
The Suzuki–Miyaura reaction in aqueous extract of suaeda salsa ^a.^

Entry	R^1^	R^2^	Time (h)	Number	Yield (%)
1	4-CN	H	1	3e	92 ^a^ 94 ^b^
2	4-NO_2_	H	1	3f	89 ^a^
3	4-CHO	H	2	3g	91 ^a^
4	4-COCH_3_	H	2	3d	94 ^a^
5	4-OH	H	1	3h	87 ^a^
6	4-COOH	H	8	3i	36 ^a^
7	4-OCH_3_	H	1	3j	67 ^b^
8	4-CH_3_	H	2	3k	13 ^b^
9	3-CN	H	1	3l	90 ^b^
10	3-NO_2_	H	2	3m	82 ^b^
11	3-OCH_3_	H	2	3n	63 ^b^
12	2-CN	H	1	3o	88 ^b^
13	2-OCH_3_	H	2	3p	72 ^b^
14	4-CN	4-F	1	3q	88 ^a^
15	4-CN	4-OCH_3_	1	3r	90 ^a^
16	4-CN	3,4-(OCH_3_)_2_	2	3s	60 ^b^
17	4-CN	4-CH_3_	2	3b	88 ^b^
18	4-CHO	4-CH_3_	1	3t	92 ^a^
19	4-CHO	4-OCH_3_	1	3u	90 ^a^
20	4-CHO	4-F	2	3a	82 ^b^
21	4-OCH_3_	4-CH_3_	4	3v	71 ^b^
22	4-OCH_3_	4-OCH_3_	4	3w	68 ^b^
23	4-OCH_3_	3,4-(OCH_3_)_2_	4	3c	85 ^b^
24	4-F	4-OCH_3_	2	3x	72 ^b^
25	4-Cl	4-OCH_3_	2	3y	70 ^b^
26	2-OCH_3_	2-CH_3_	2	3z	30 ^b^
27	2-CN	2-CH_3_	2	3aa	36 ^b^
28	4-CN	H	8	3e	trace ^c^
29	4-CN	H	4	3e	49 ^d^
30	4-CN	H	4	3e	trace _e_

^a^ Reaction condition: Aryl halide (0.5 mmol), arylboronic acid (0.525 mmol), 10 wt.% Pd/C (0.2 mol%), WES (3 mL), 100 °C, the product is obtained by filtration. ^b^ The product is obtained by column chromatography. ^c^ Phenylboronic acid pinacol ester instead of phenylboronic acid. ^d^ Potassium phenyltrifluoroborate instead of phenylboronic acid. ^e^ Phenylboronic acid MIDA ester instead of phenylboronic acid.

**Table 3:**
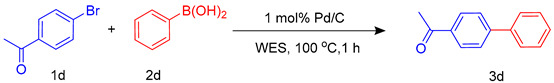
Catalyst recovery of Suzuki–Miyaura reaction with Suaeda salsa extract as solution.

Entry	Time (h)	Yield (%)
1	1.0	95
2	1.0	90
3	1.0	83
4	2.0	71
5	8.0	52

Reaction condition: 4-bromoacetophenone (6 mmol), phenylboronic acid (6.3 mmol), 10 wt. %Pd/C (1 mol%), WES (36 mL), under air, 1 h, isolated yield by column chromatography.

## Data Availability

The data presented in this study are available in article or Supplementary Materials.

## Supplementary Materials

The following supporting information can be downloaded at: https://www.mdpi.com/article/10.3390/molecules27196623/s1, Supplementary Materials file (^1^H and ^13^C NMR spectra).

## Sample Availability

Samples of the prepared compounds are available from the authors
