# Supplementary material for: A Novel Utilization of Water Extract of Suaeda Salsa in the Pd/C Catalyzed Suzuki–Miyaura Coupling Reaction
Source: Molecules. 2022 Oct 6;27(19):6623. doi: 10.3390/molecules27196623 (PMC9573658; doi:10.3390/molecules27196623)
Supplement: Supplementary file 1 [file molecules-27-06623-s001.zip › molecules-1942828-supplementary.pdf]

# A Novel Utilization of Water Extract of Suaeda Salsa in the Pd/C Catalyzed Suzuki-Miyaura Coupling Reaction

Changyue Ren, [1] Hang Zhang, [1] Zhengjun Chen, [1] Jie Gao, [1, 2, 3, *] Mingyan Yang, [1, 2, 3,] Zeli Yuan, [1, 2, 3,*] Xinmin Li[1, 2, 3, *]

[1] College of Pharmacy, Zunyi Medical University, Zunyi, Guizhou 563003, China;

[2] Key Laboratory of Basic Pharmacology of Ministry of Education and Joint International Research Laboratory of Ethnomedicine of Ministry of Education, Zunyi Medical University, Zunyi, Guizhou 563000, China;

[3] Guizhou International Scientific and Technological Cooperation Base for Medical Photo-Theranostics Technology and Innovative Drug Development, Zunyi, Guizhou 563003, China.

* Correspondence: jiegao@mail.nankai.edu.cn; zlyuan@zmu.edu.cn; lixm@zmu.edu.cn

**Contents**

**1. Materials and Methods and Experimental Procedure** **S1-S2**

**2. Plausible Mechanism for the Suzuki-Miyaura Reaction in WES** **S2**

**3. NMR Spectra for Cross-Coupling Products** **S3-S42**

## 1. Materials, Methods and Experimental Procedure

### 1.1 General remarks

All commercially available reagents (from Acros, Aldrich, Fluka, Energy Chemical) were used without further purification. 10 wt. %Pd/C was purchased from Energy Chemical (Palladium 10% on Carbon, ca. 50% water). All reactions were carried out under air. NMR spectra were recorded on a Brucker Advance II 400 spectrometer using TMS as internal standard (400 MHz for $^{1}H$ NMR and $^{13}C$ NMR). The isolated yield of products was obtained by short chromatography on a silica gel (200-300 mesh) column using petroleum ether (60-90 ºC) and ethyl acetate, unless otherwise noted.

### 1.2 The procedure for preparing water extract of suaeda salsa

The suaeda salsa was dried overnight in an oven at 120 $^{o}C$, then the dried suaeda salsa was burned to ash. After that, 10 g of the ash was suspended in 100 mL distilled water and stirred for 2 hours at 100 $^{o}C$. The suspension was filtered, and the light yellow colored solution was next concentrated to give a yellow solid substance which was used to prepare suaeda salsa aqueous solution with a concentration of 23 mg/mL for Suzuki cross-coupling reactions.

### 1.3 General procedure for the synthesis of biaryl and heteroaryl compounds.

A mixture of aryl halide (0.5 mmol), arylboronic acid (0.525 mmol), 10 wt.% Pd/C (0.2-1 mol%), WES solution (23 mg/mL, 3 mL) was stirred at 100 ℃ under air for the indicated time. After reaction, the mixture was cooled to room temperature. [a] The mixture was filtered and washed by water (10 mL) for three times. The residue was dissolved in ethyl acetate (20 mL) and then filtered to remove the palladium carbon. Then the ethyl acetate was collected and concentrated in vacuo to give the pure product. [b] The mixture was concentrated in vacuo and the product was isolated by short chromatography on a silica gel (200-300 mesh) column using petroleum ether and ethyl acetate.

## 2. Plausible Mechanism for the Suzuki-Miyaura Reaction in WES

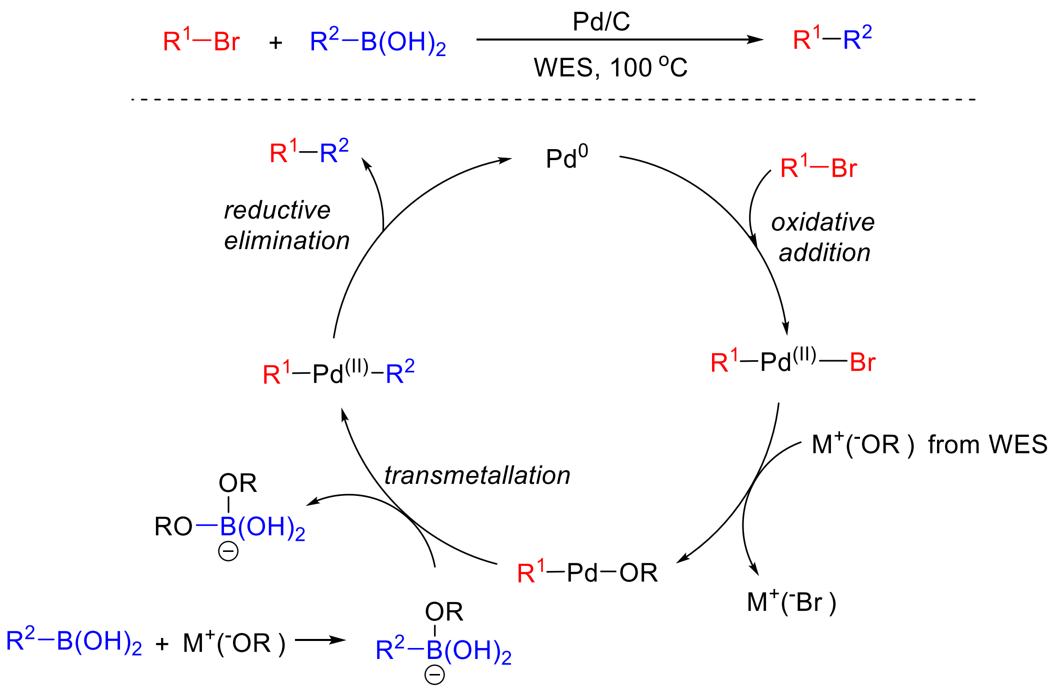


**Scheme S1**. Plausible mechanism for the Suzuki-Miyaura Reaction in WES

## 3. NMR Spectra for Cross-Coupling Products

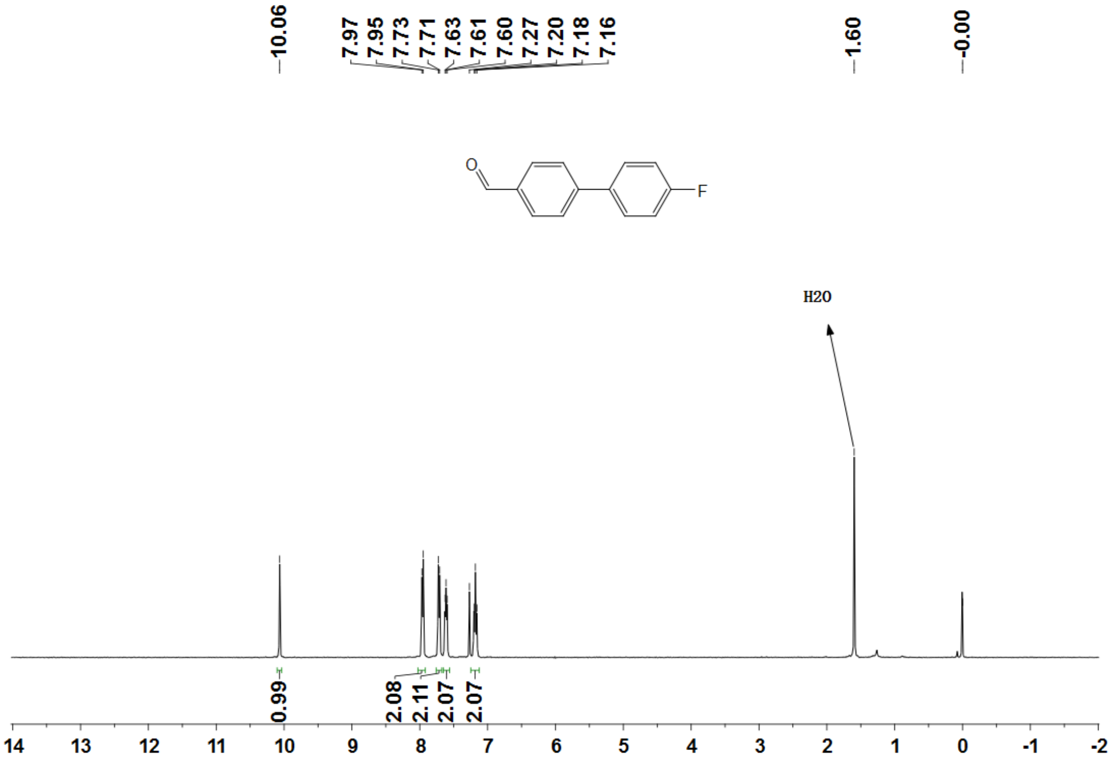


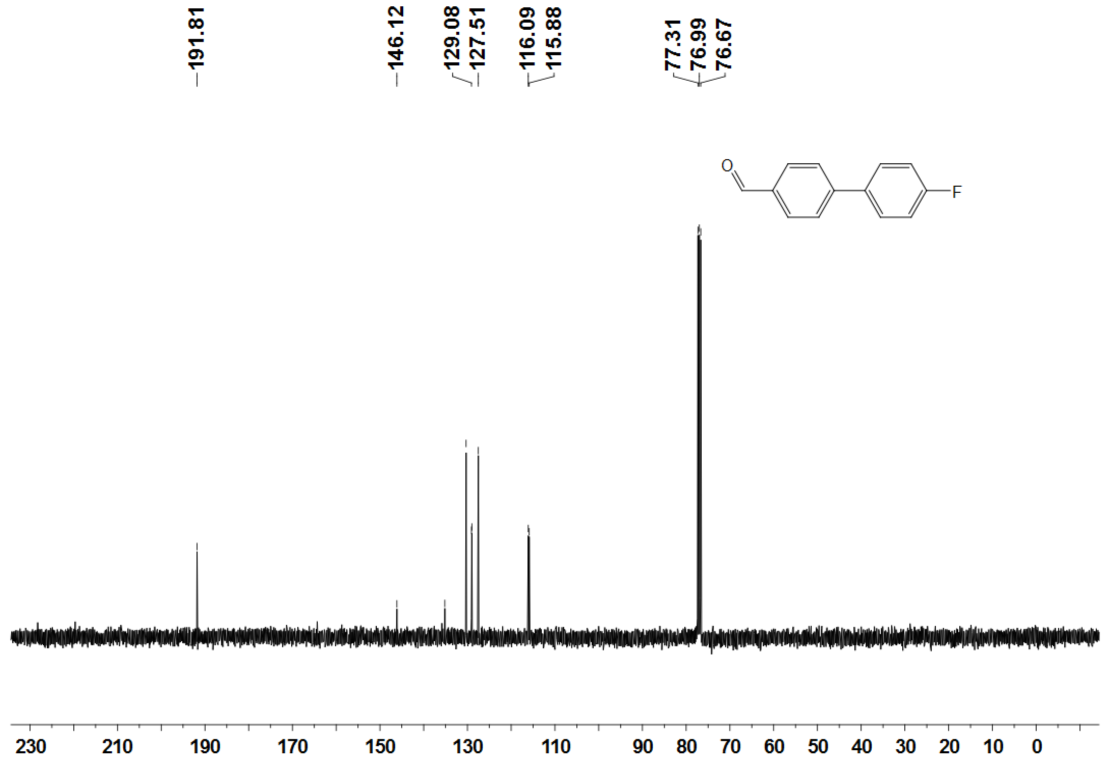

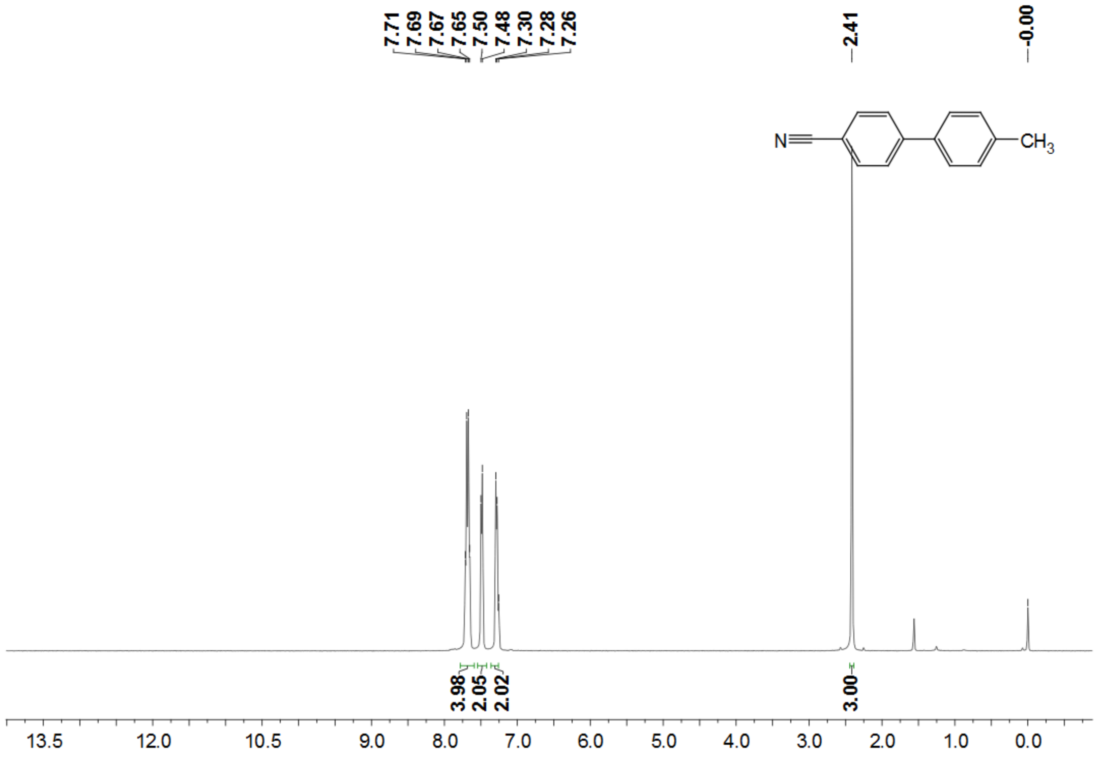
7.71
7.69
7.67
7.65
7.50
7.48
7.30
7.28
7.26
2.41
-0.00
N
CH3
3.98
2.05
2.02
3.00
13.5
12.0
10.5
9.0
8.0
7.0
6.0
5.0
4.0
3.0
2.0
1.0
0.0

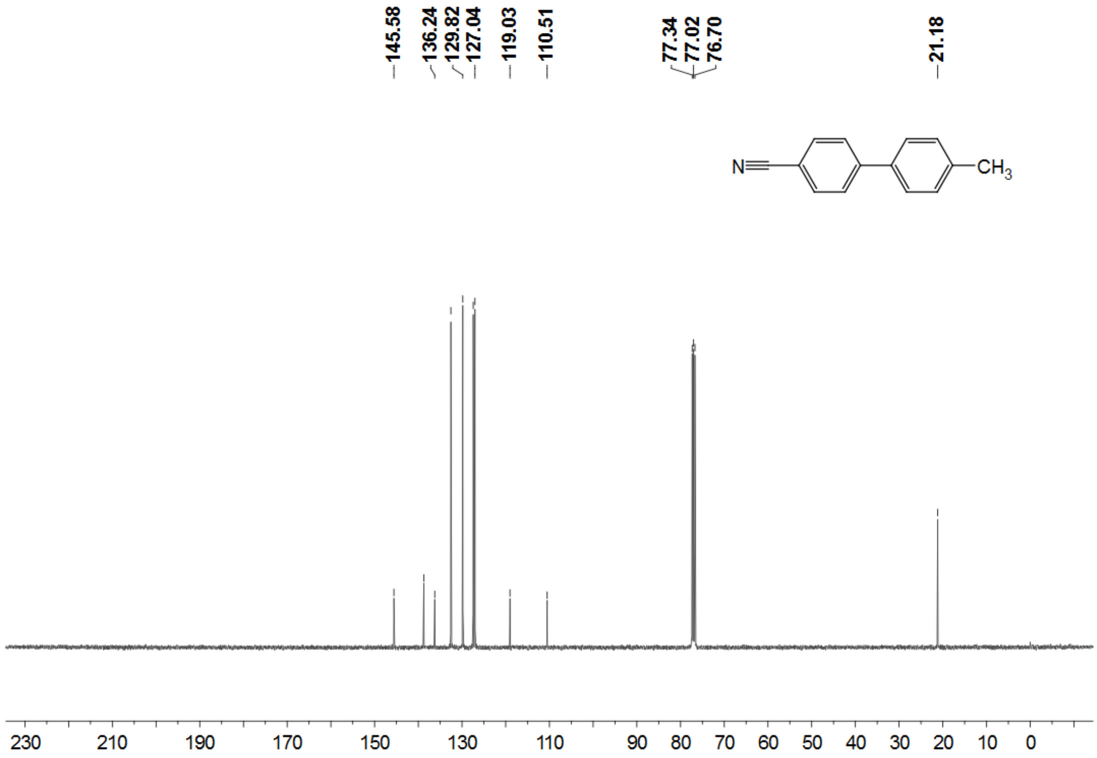
145.58
136.24
129.82
127.04
119.03
110.51
77.34
77.02
76.70
21.18
N
CH3
230
210
190
170
150
130
110
90
80
70
60
50
40
30
20
10
0

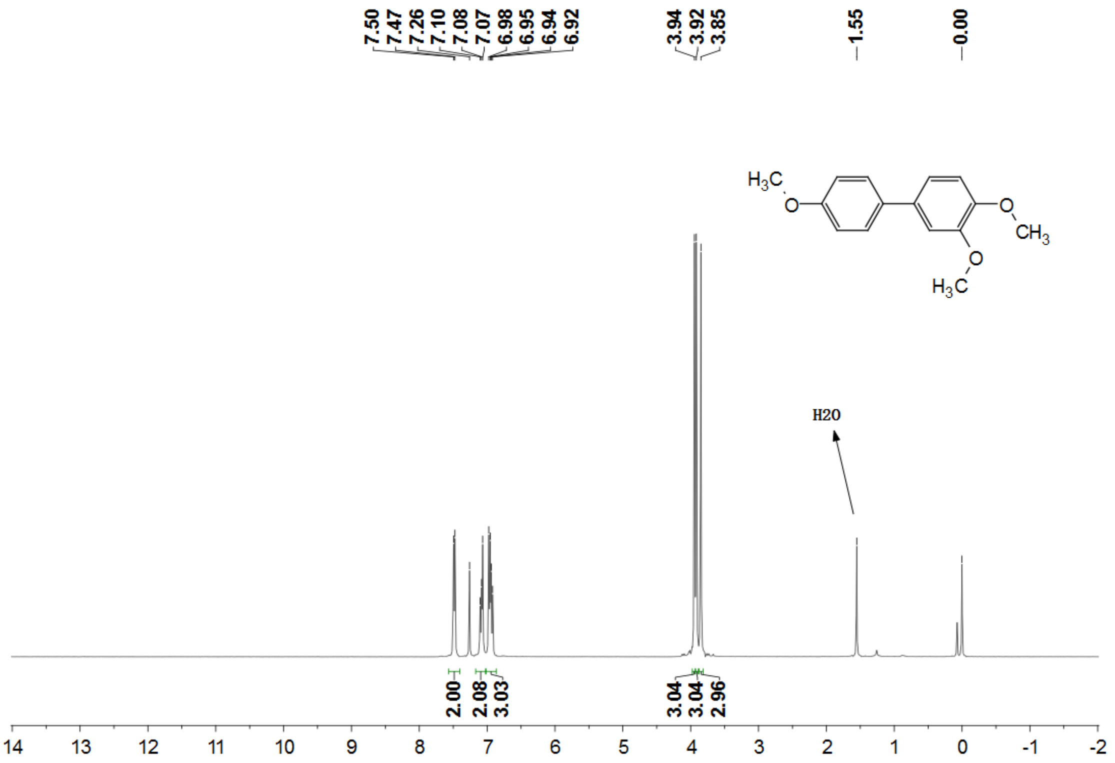

7.50
7.47
7.26
7.10
7.08
7.07
6.98
6.95
6.94
6.92
3.94
3.92
3.85
1.55
0.00
H2O
2.00
2.08
3.03
3.04
3.04
2.96
14 13 12 11 10 9 8 7 6 5 4 3 2 1 0 -1 -2


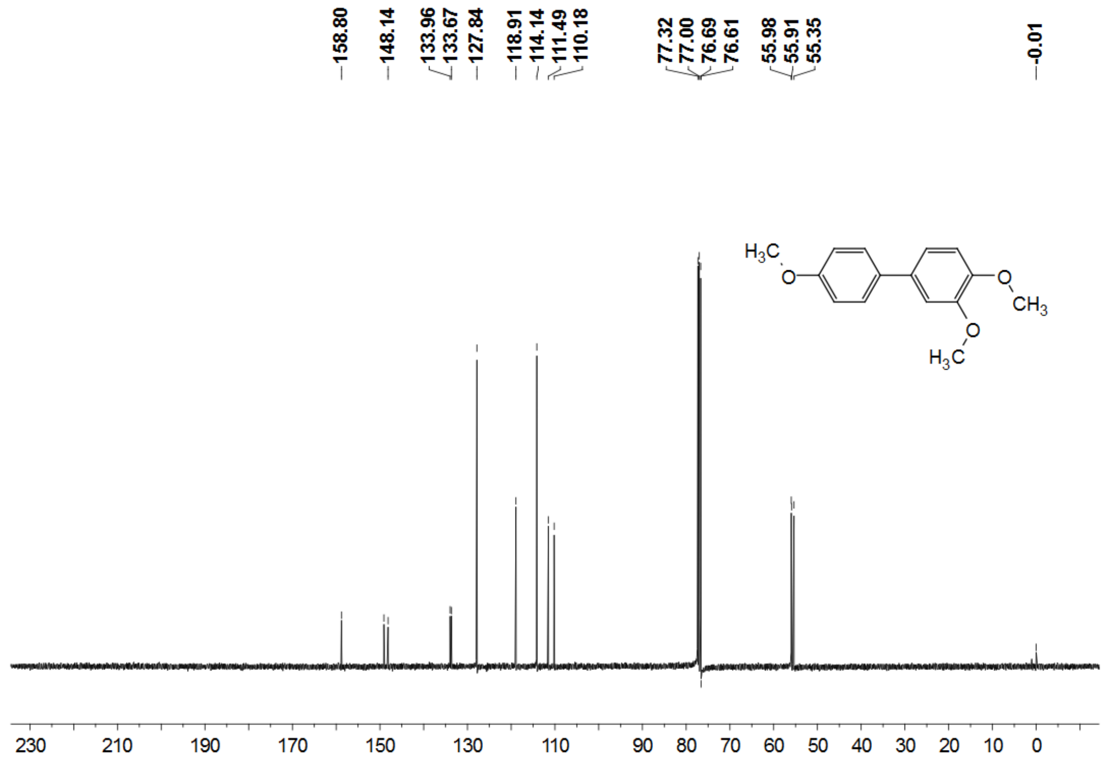

158.80
148.14
133.96
133.67
127.84
118.91
114.14
111.49
110.18
77.32
77.00
76.69
76.61
55.98
55.91
55.35
-0.01
230 210 190 170 150 130 110 90 80 70 60 50 40 30 20 10 0

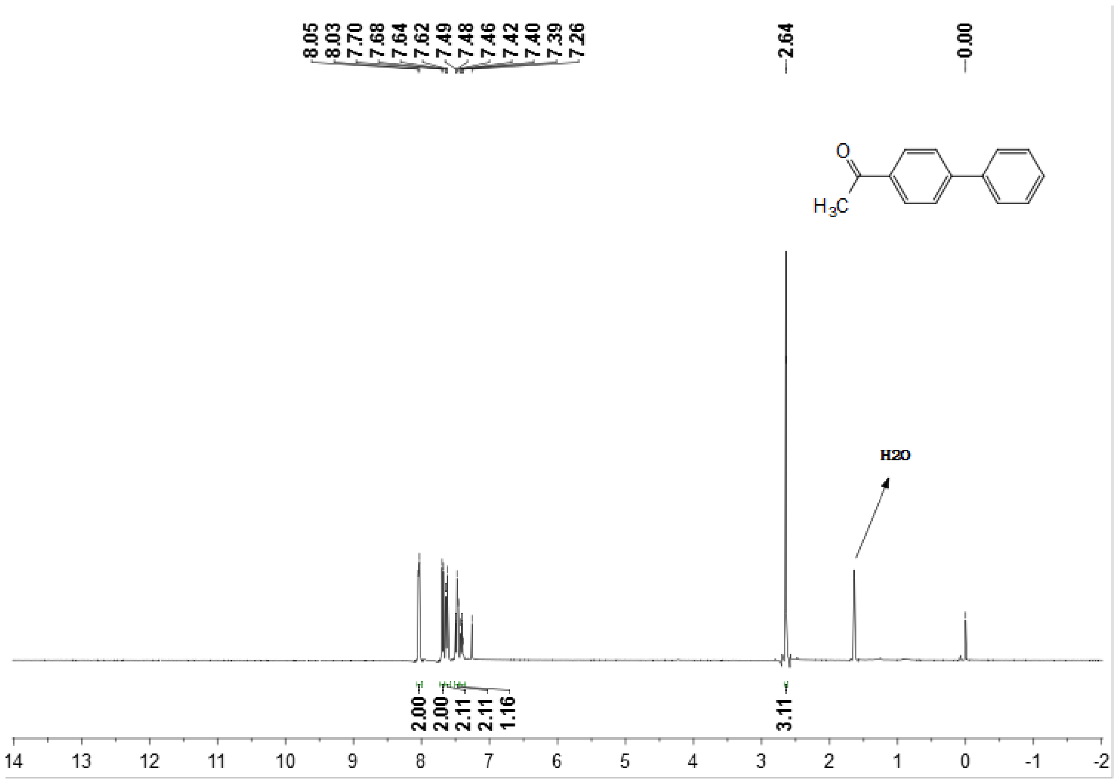
8.05
8.03
7.70
7.68
7.64
7.62
7.49
7.48
7.46
7.42
7.40
7.39
7.26
2.64
0.00
O
H3C
H2O
2.00
2.00
2.11
2.11
1.16
3.11
14 13 12 11 10 9 8 7 6 5 4 3 2 1 0 -1 -2

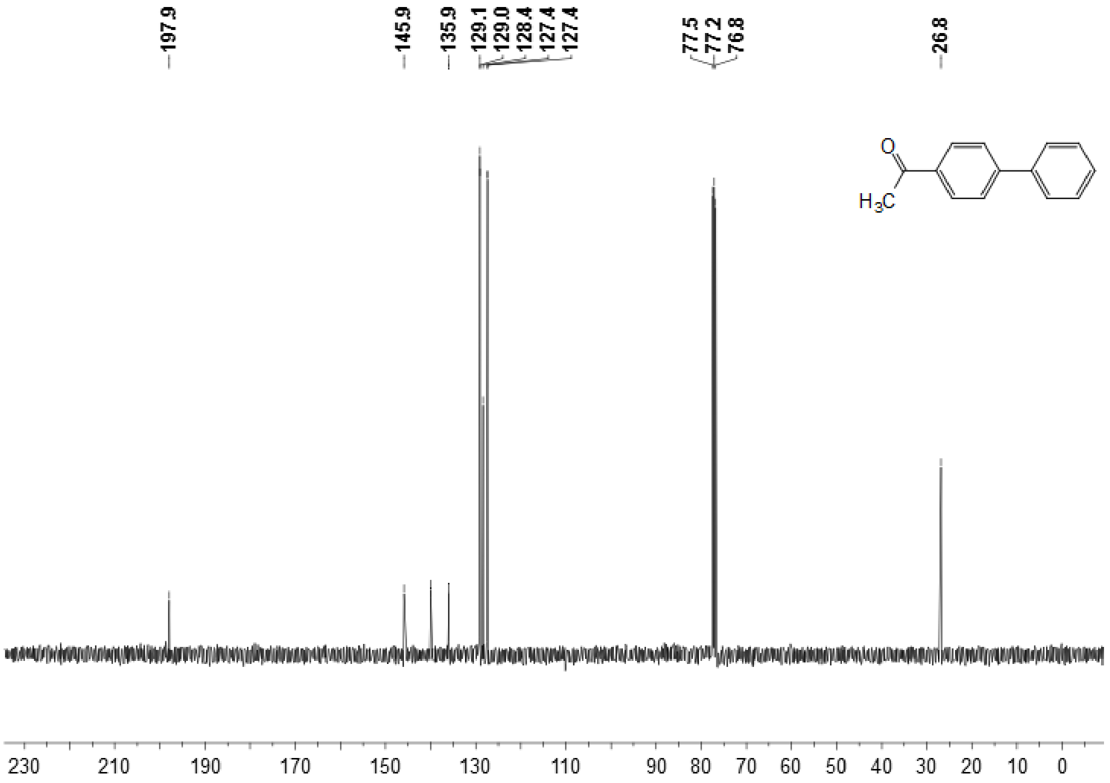
197.9
145.9
135.9
129.1
129.0
128.4
127.4
127.4
77.5
77.2
76.8
26.8
O
H3C
230 210 190 170 150 130 110 90 80 70 60 50 40 30 20 10 0

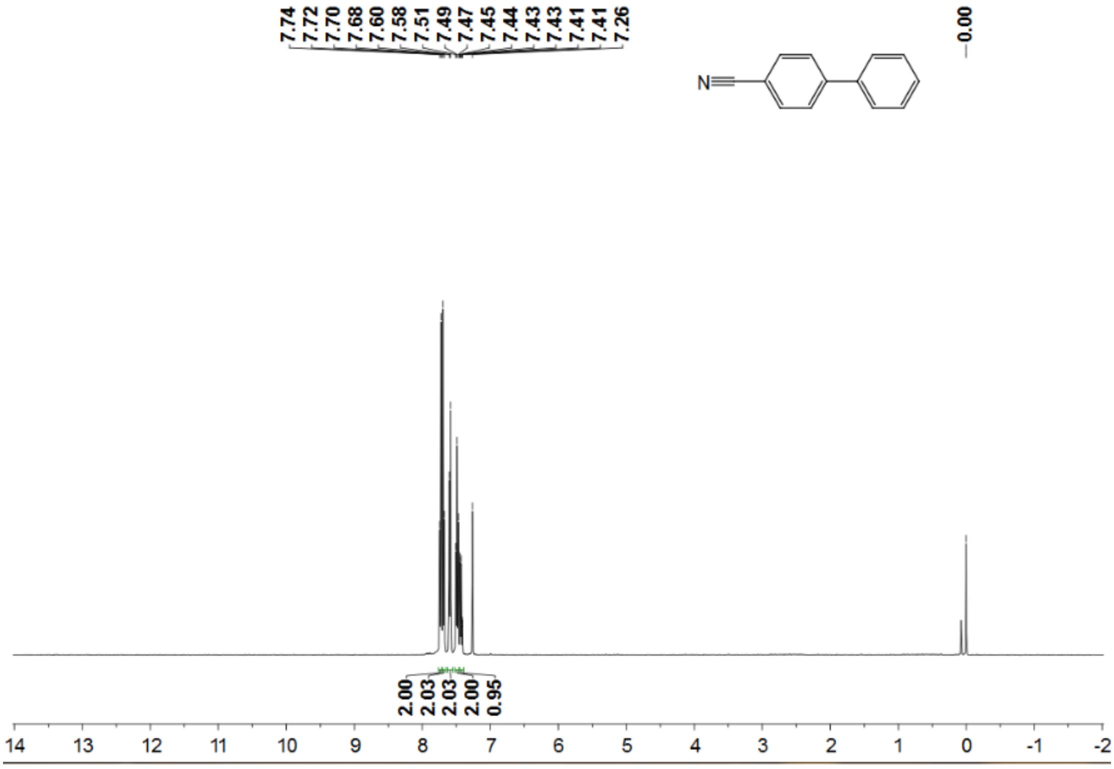
7.74
7.72
7.70
7.68
7.60
7.58
7.51
7.49
7.47
7.45
7.44
7.43
7.43
7.41
7.41
7.26
0.00
N
2.00
2.03
2.03
2.00
0.95
14 13 12 11 10 9 8 7 6 5 4 3 2 1 0 -1 -2

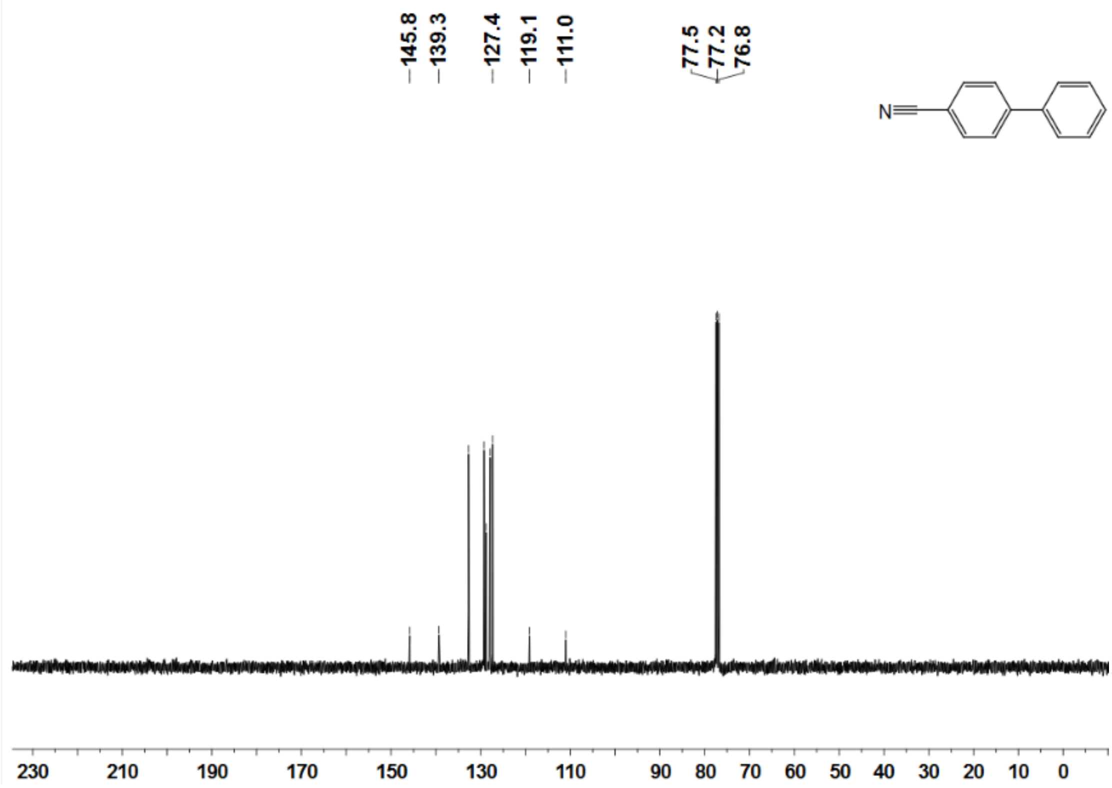
145.8
139.3
127.4
119.1
111.0
77.5
77.2
76.8
N
230 210 190 170 150 130 110 90 80 70 60 50 40 30 20 10 0

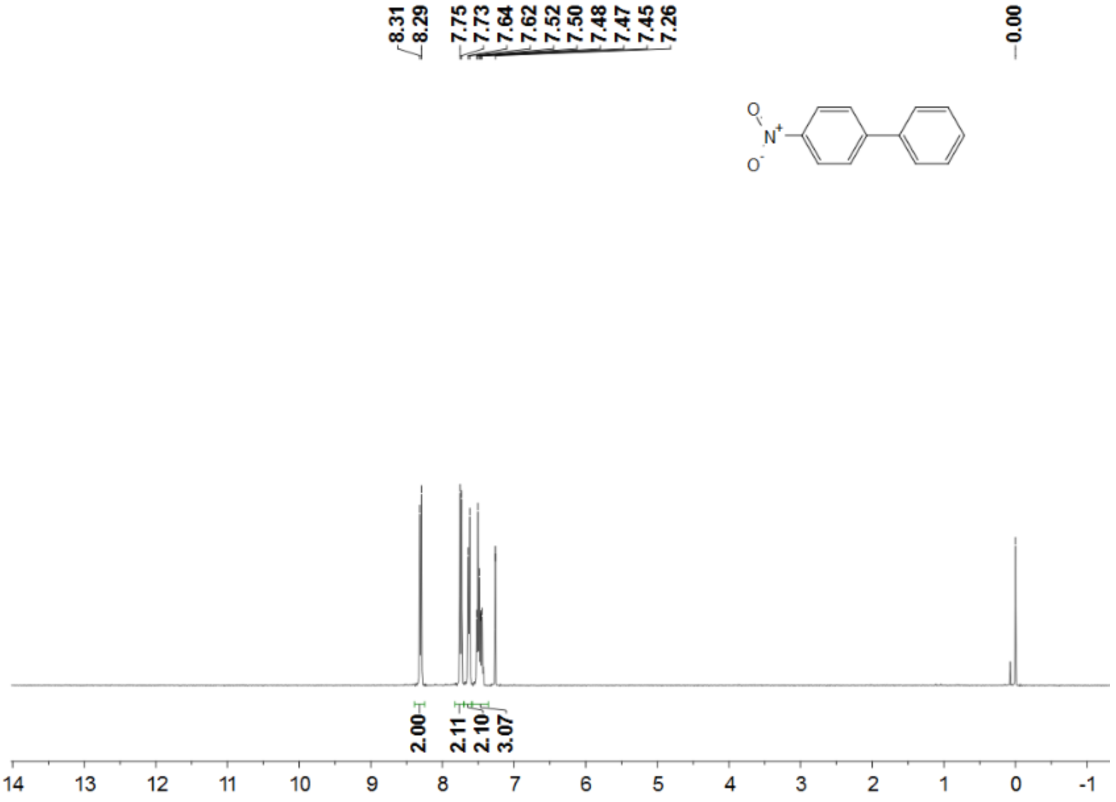
8.31
8.29
7.75
7.73
7.64
7.62
7.52
7.50
7.48
7.47
7.45
7.26
0.00
2.00
2.11
2.10
3.07
14 13 12 11 10 9 8 7 6 5 4 3 2 1 0 -1

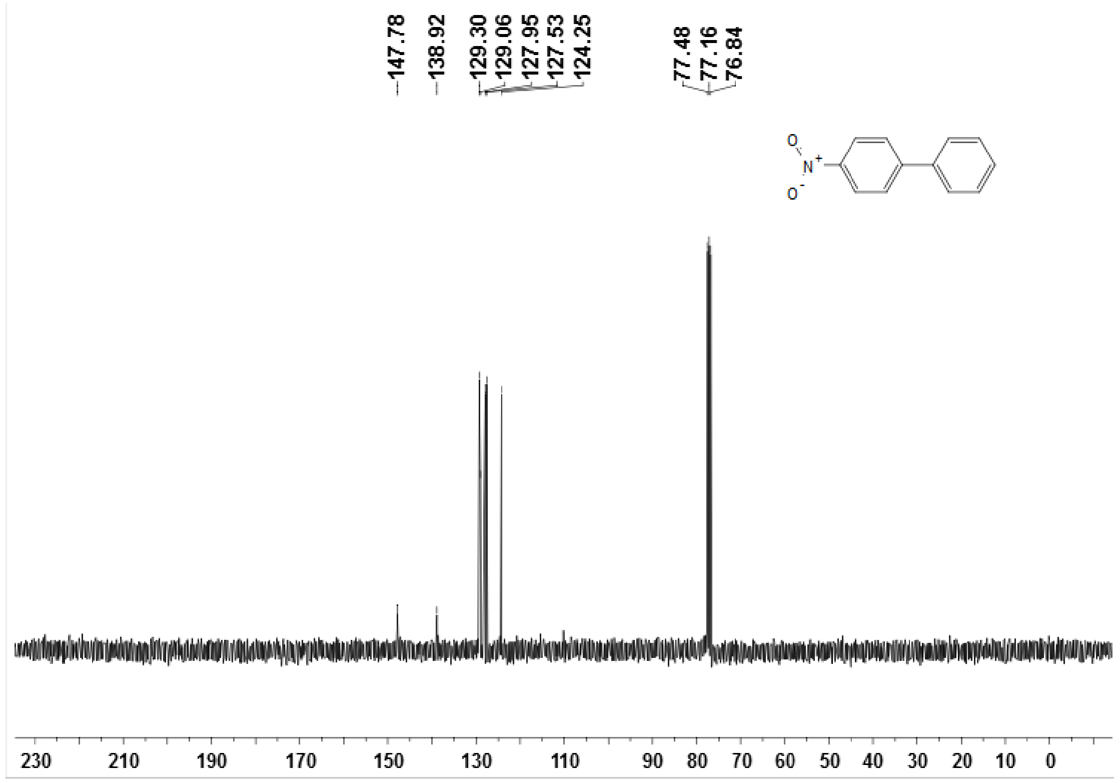
147.78
138.92
129.30
129.06
127.95
127.53
124.25
77.48
77.16
76.84
230 210 190 170 150 130 110 90 80 70 60 50 40 30 20 10 0

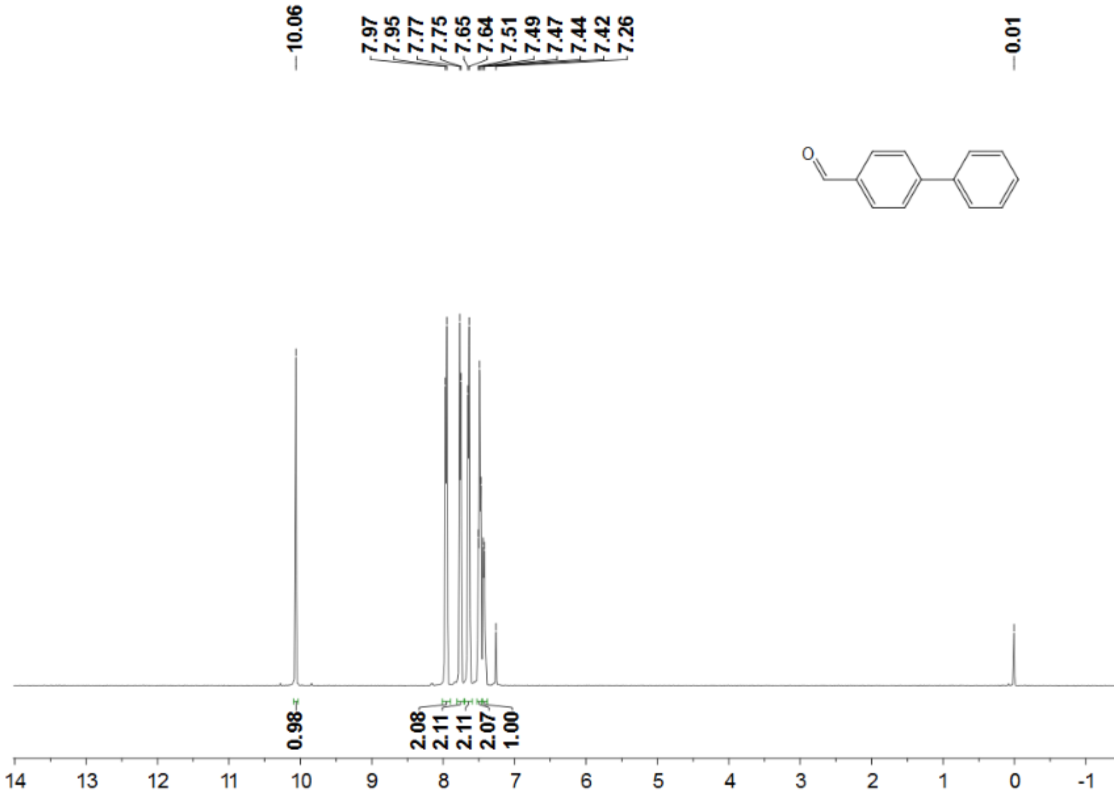
10.06
7.97
7.95
7.77
7.75
7.65
7.64
7.51
7.49
7.47
7.44
7.42
7.26
0.01
O
0.98
2.08
2.11
2.11
2.07
1.00
14
13
12
11
10
9
8
7
6
5
4
3
2
1
0
-1

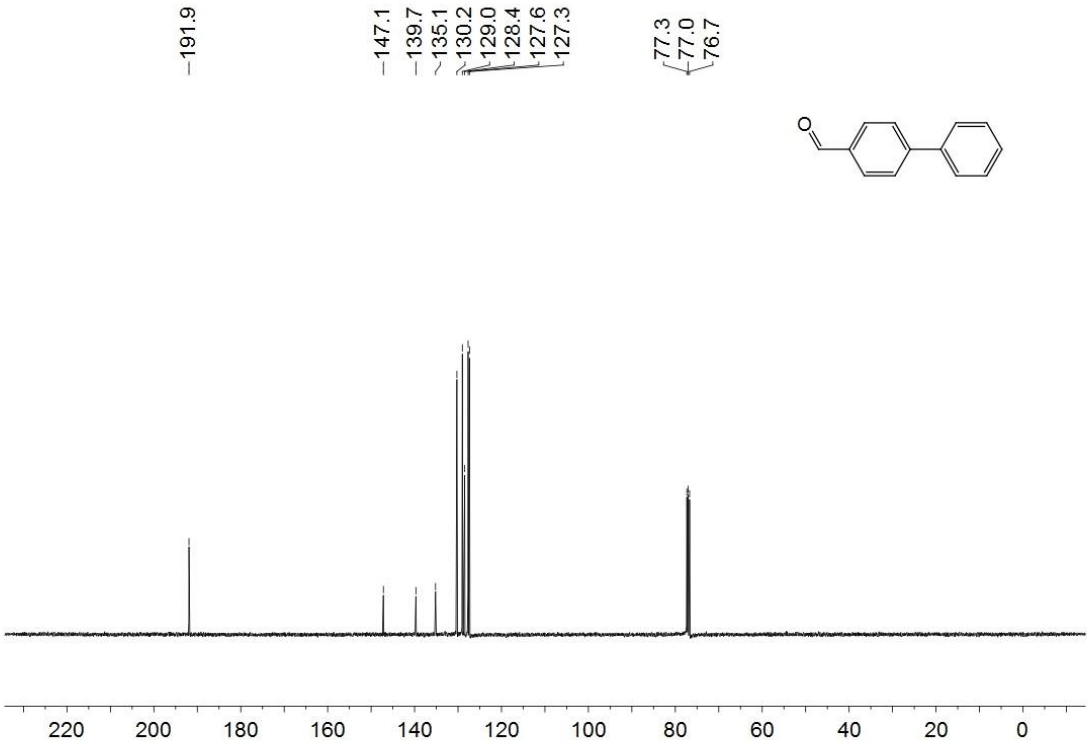
191.9
147.1
139.7
135.1
130.2
129.0
128.4
127.6
127.3
77.3
77.0
76.7
O
220
200
180
160
140
120
100
80
60
40
20
0

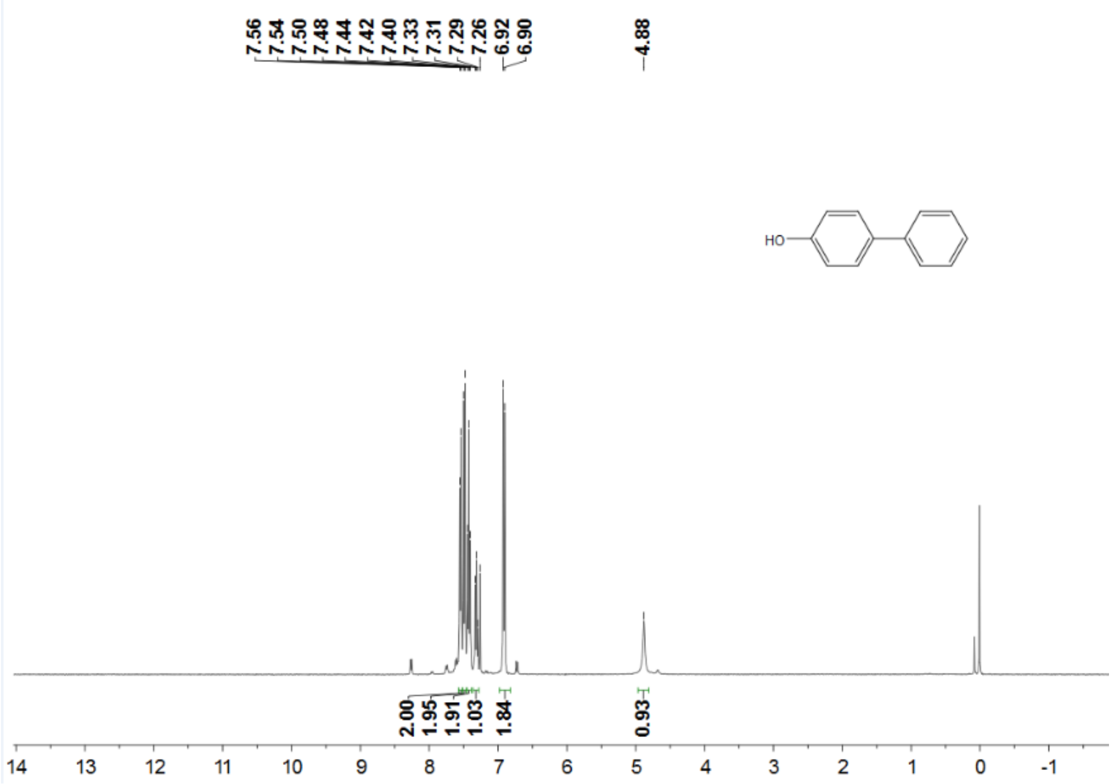
7.56
7.54
7.50
7.48
7.44
7.42
7.40
7.33
7.31
7.29
7.26
6.92
6.90
4.88
HO
2.00
1.95
1.91
1.03
1.84
0.93
14 13 12 11 10 9 8 7 6 5 4 3 2 1 0 -1

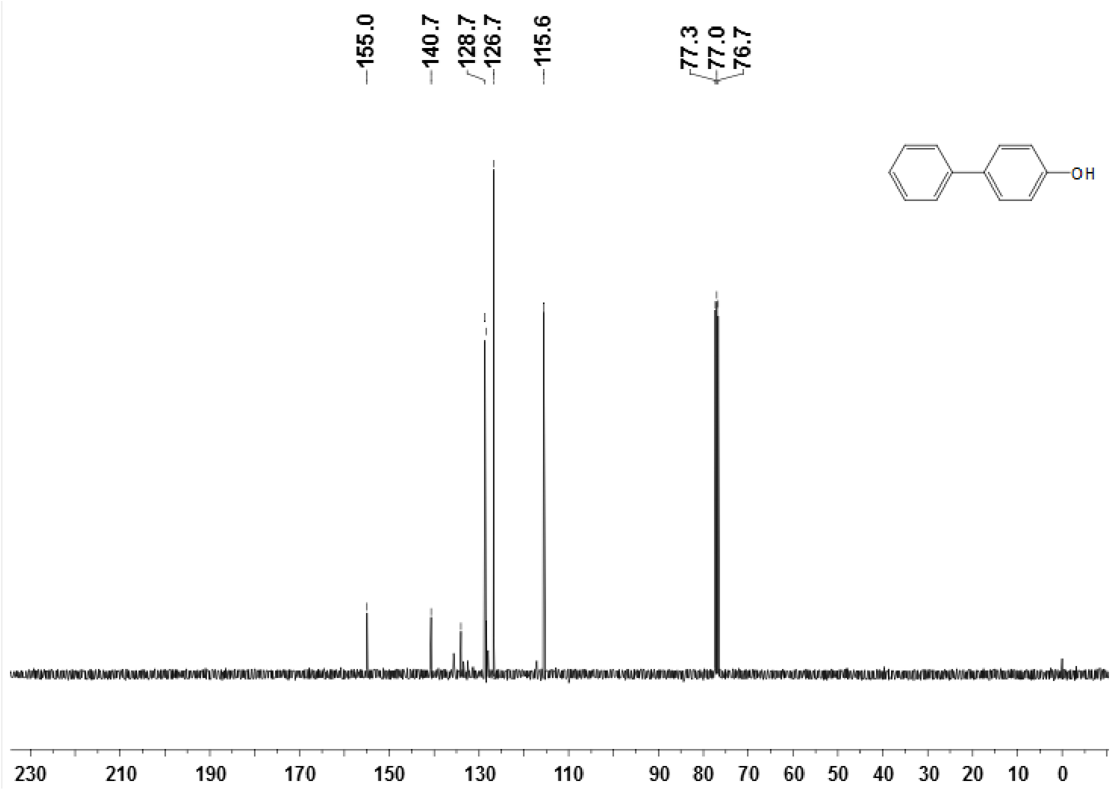
155.0
140.7
128.7
126.7
115.6
77.3
77.0
76.7
OH
230 210 190 170 150 130 110 90 80 70 60 50 40 30 20 10 0

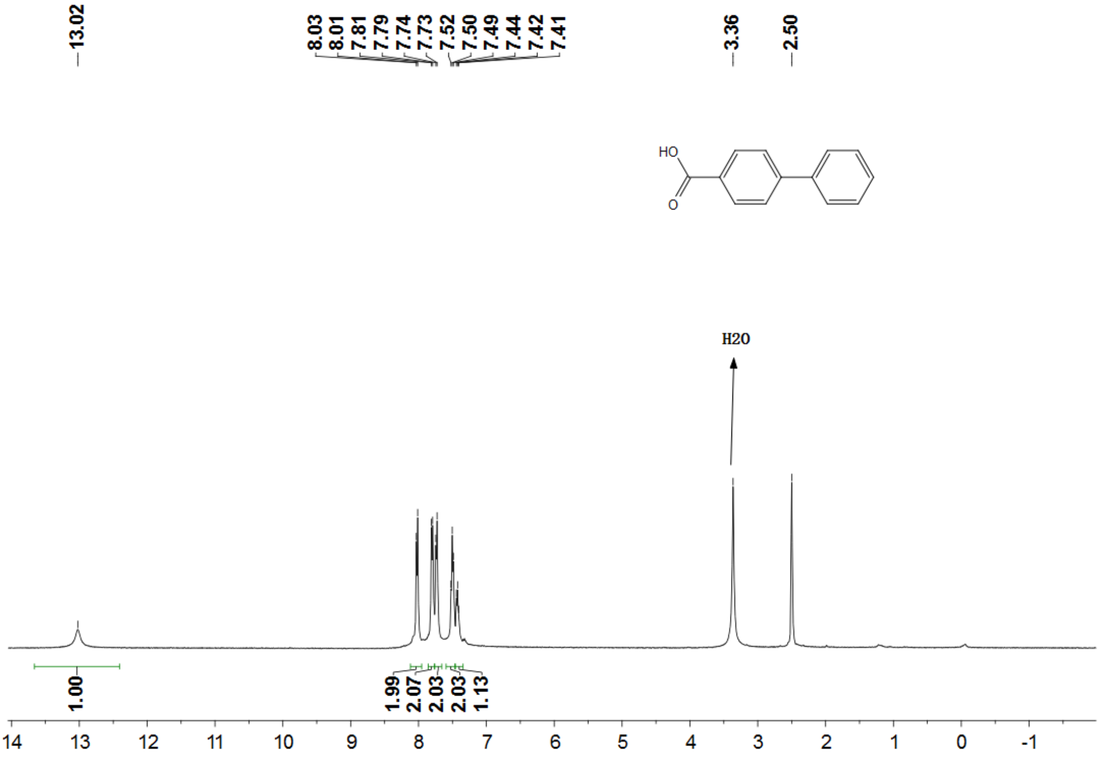
13.02
8.03
8.01
7.81
7.79
7.74
7.73
7.52
7.50
7.49
7.44
7.42
7.41
3.36
2.50
HO
O
H2O
1.00
1.99
2.07
2.03
2.03
1.13
14
13
12
11
10
9
8
7
6
5
4
3
2
1
0
-1

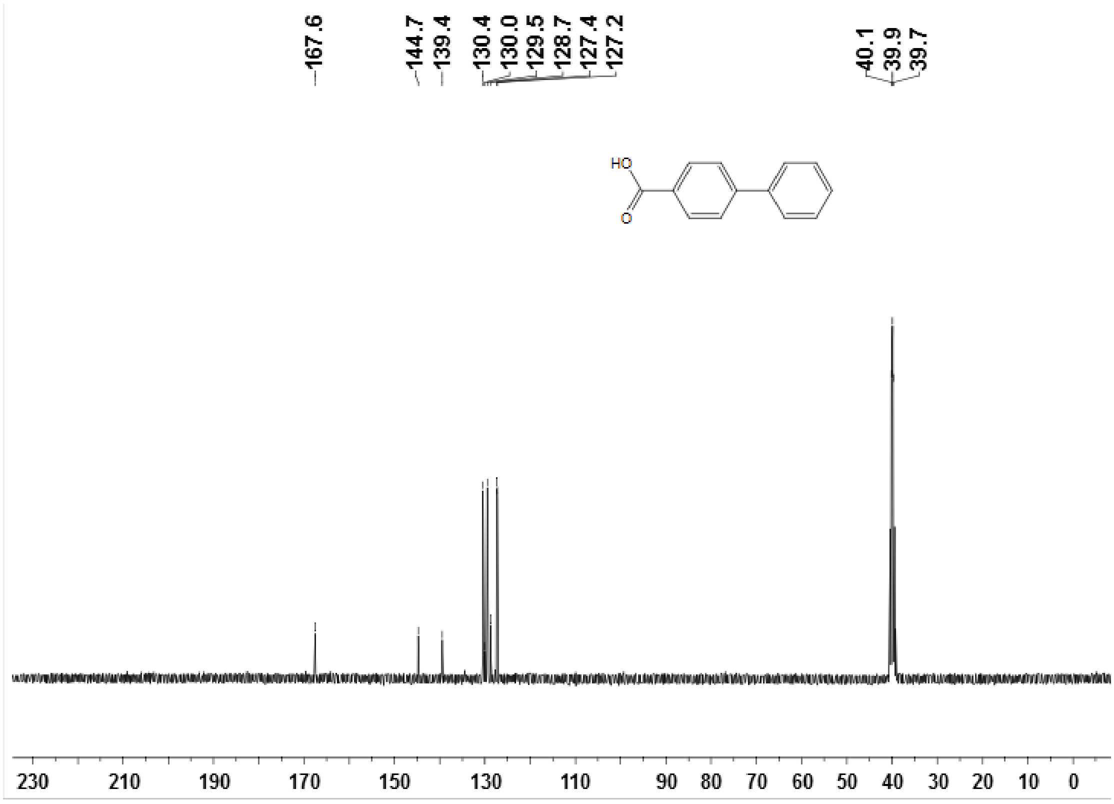
167.6
144.7
139.4
130.4
130.0
129.5
128.7
127.4
127.2
40.1
39.9
39.7
HO
O
230
210
190
170
150
130
110
90
80
70
60
50
40
30
20
10
0

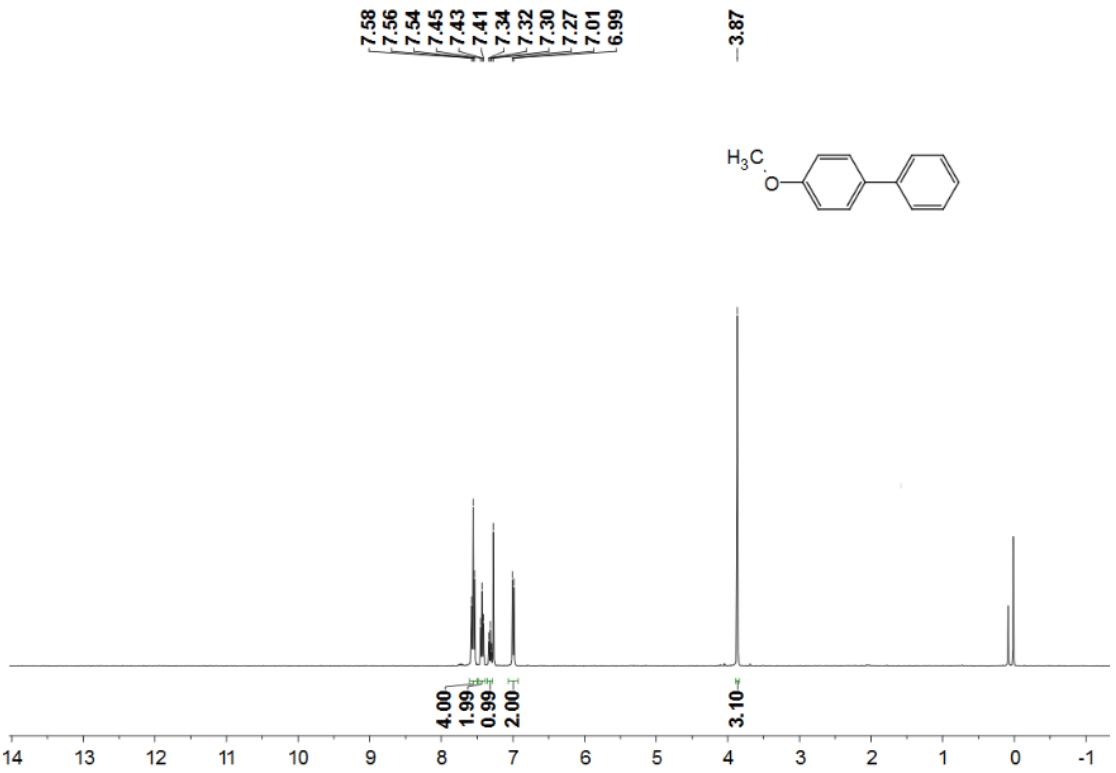

7.58
7.56
7.54
7.45
7.43
7.41
7.34
7.32
7.30
7.27
7.01
6.99
3.87
H3C
O
4.00
1.99
0.99
2.00
3.10


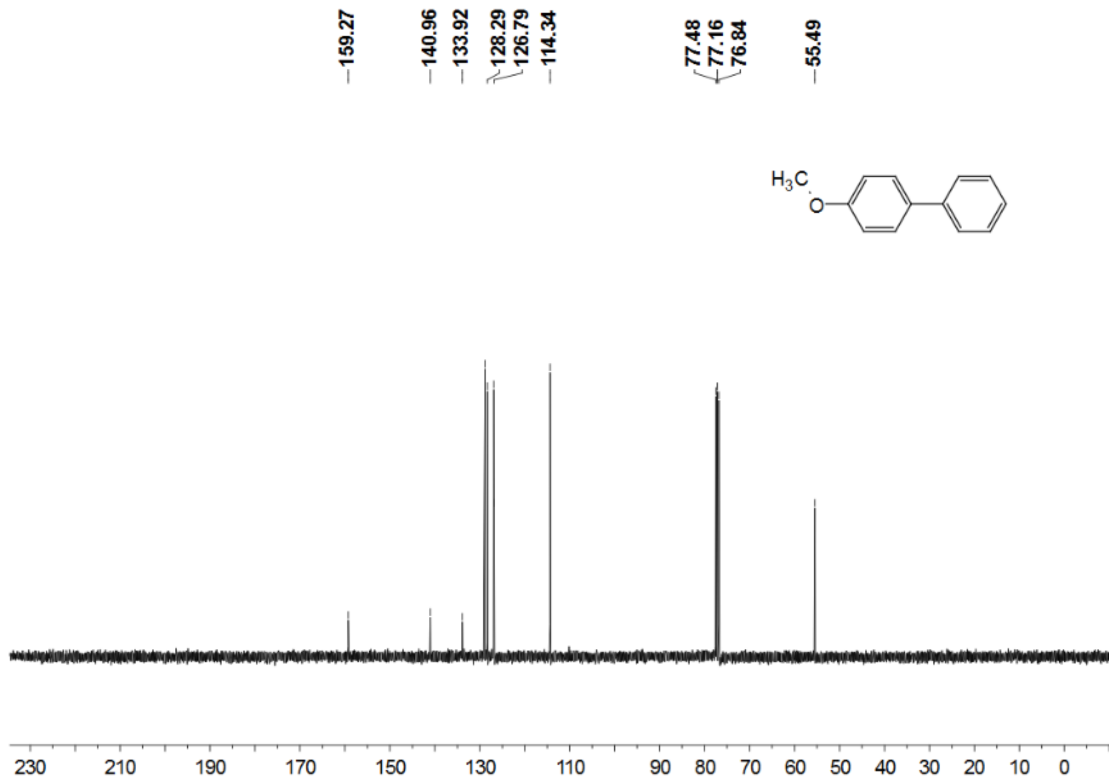

159.27
140.96
133.92
128.29
126.79
114.34
77.48
77.16
76.84
55.49
H3C
O

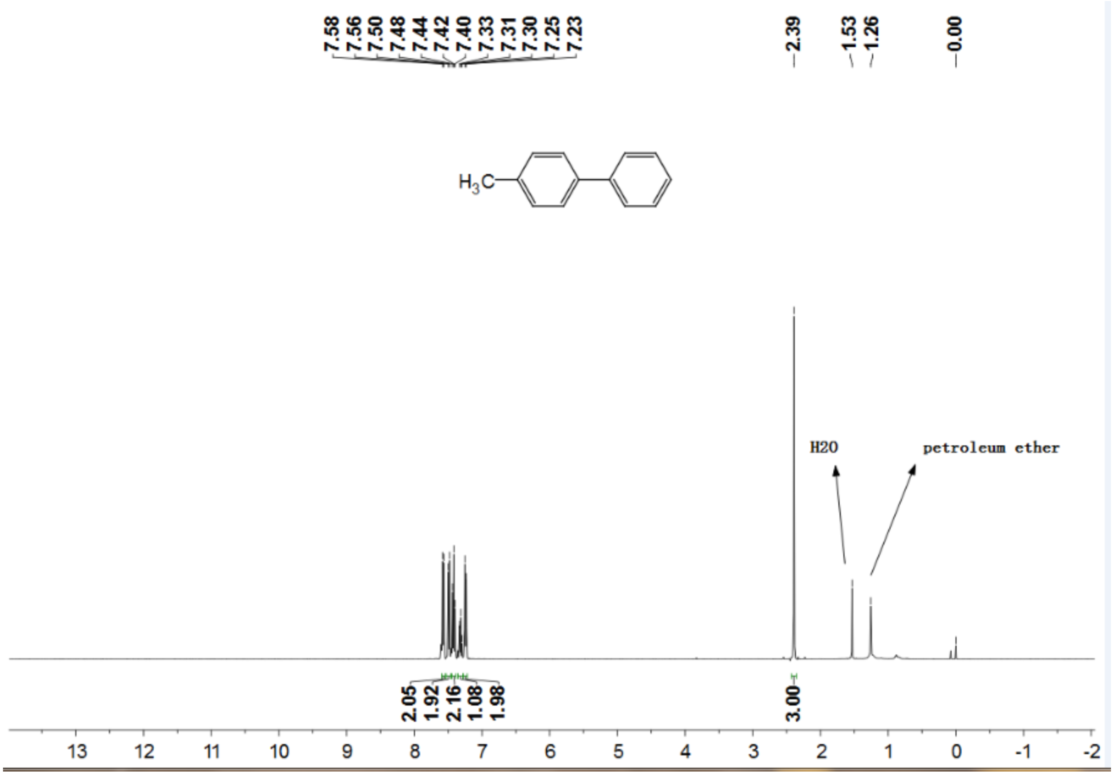
7.58
7.56
7.50
7.48
7.44
7.42
7.40
7.33
7.31
7.30
7.25
7.23
2.39
1.53
1.26
0.00
$H_3C$
H2O
petroleum ether
2.05
1.92
2.16
1.08
1.98
3.00
13 12 11 10 9 8 7 6 5 4 3 2 1 0 -1 -2

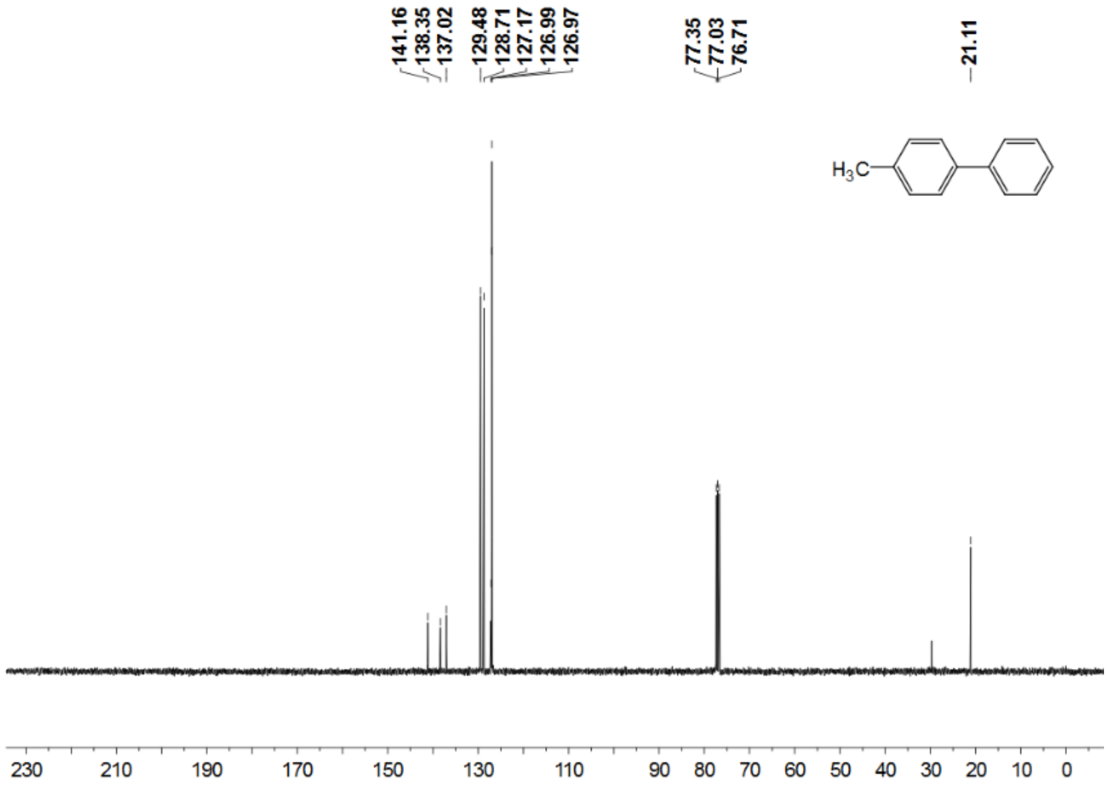
141.16
138.35
137.02
129.48
128.71
127.17
126.99
126.97
77.35
77.03
76.71
21.11
$H_3C$
230 210 190 170 150 130 110 90 80 70 60 50 40 30 20 10 0

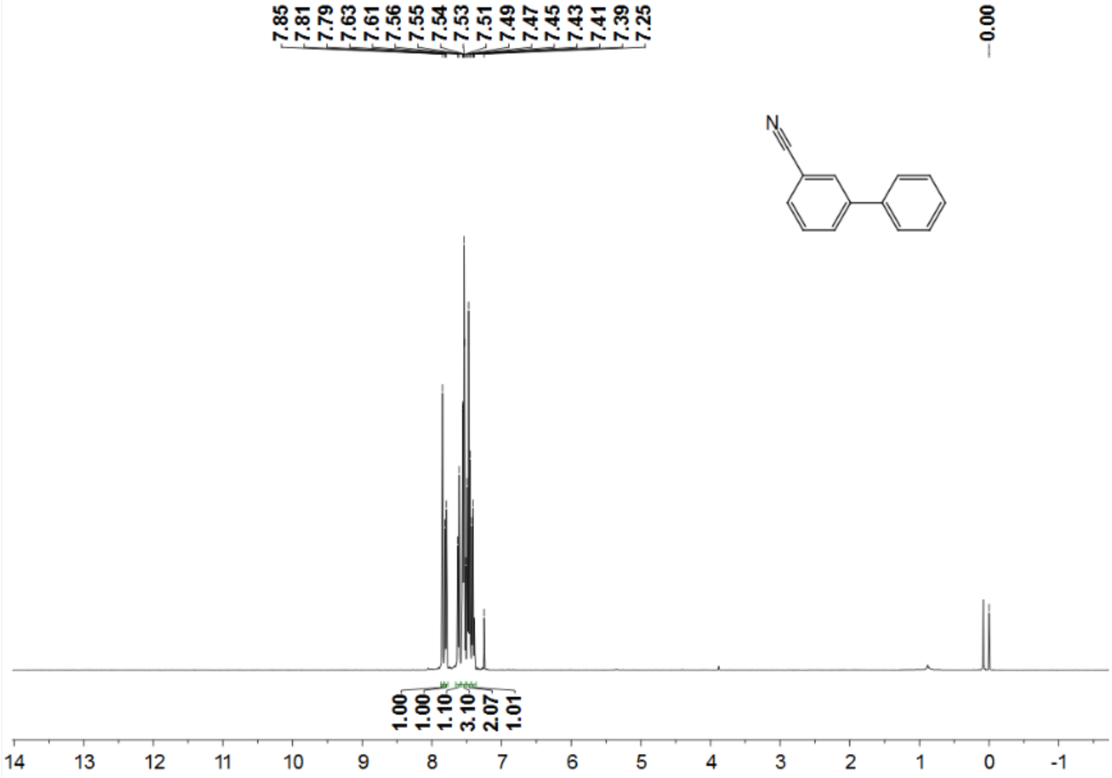
7.85
7.81
7.79
7.63
7.61
7.56
7.55
7.54
7.53
7.51
7.49
7.47
7.45
7.43
7.41
7.39
7.25
0.00
N
1.00
1.00
1.10
3.10
2.07
1.01
14 13 12 11 10 9 8 7 6 5 4 3 2 1 0 -1

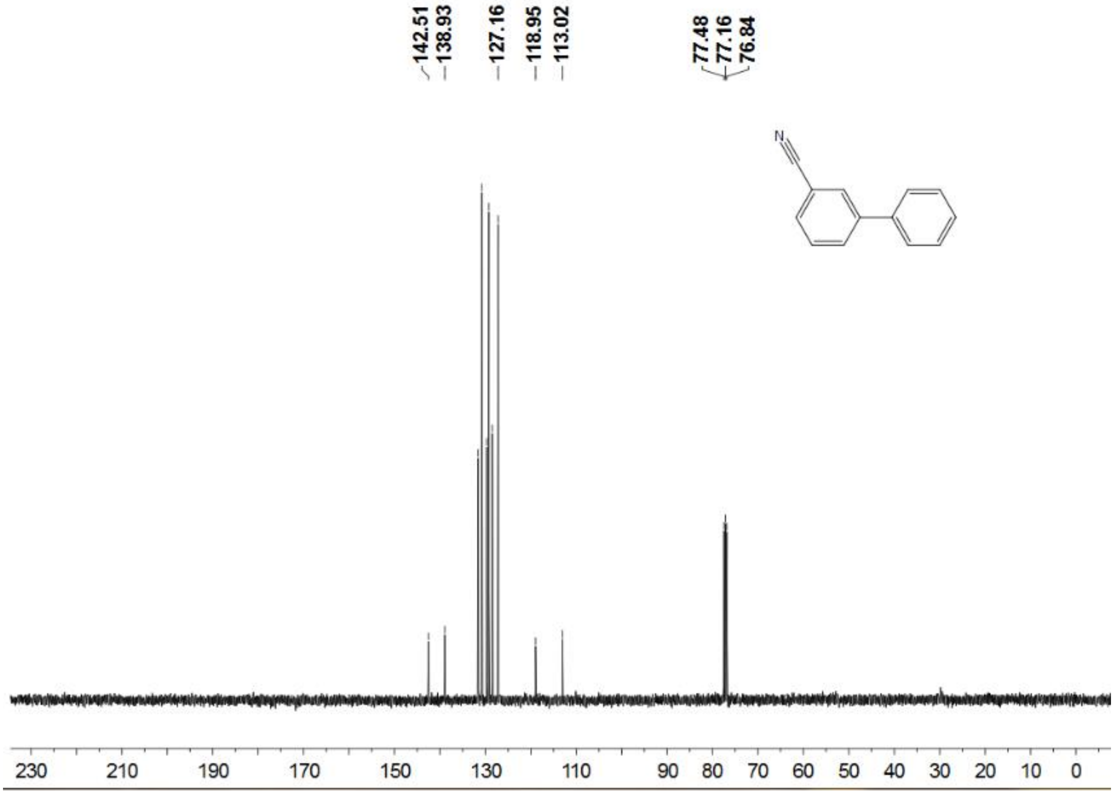
142.51
138.93
127.16
118.95
113.02
77.48
77.16
76.84
N
230 210 190 170 150 130 110 90 80 70 60 50 40 30 20 10 0

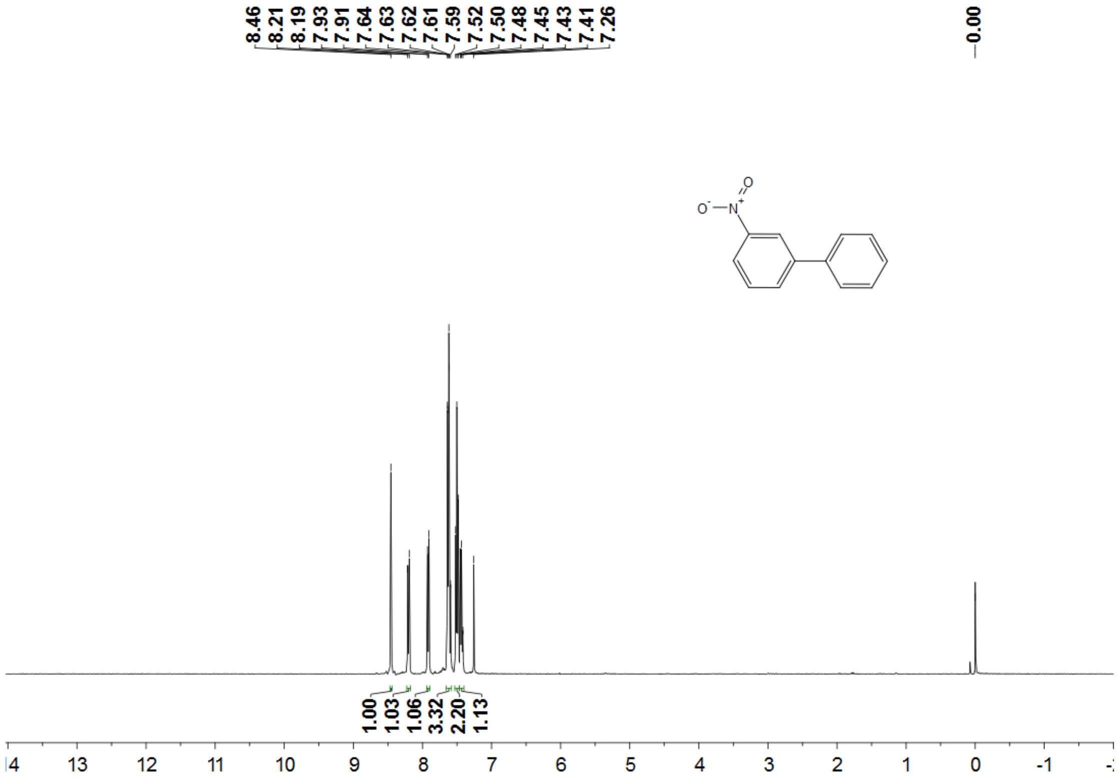
8.46
8.21
8.19
7.93
7.91
7.64
7.63
7.62
7.61
7.59
7.52
7.50
7.48
7.45
7.43
7.41
7.26
0.00
1.00
1.03
1.06
3.32
2.20
1.13

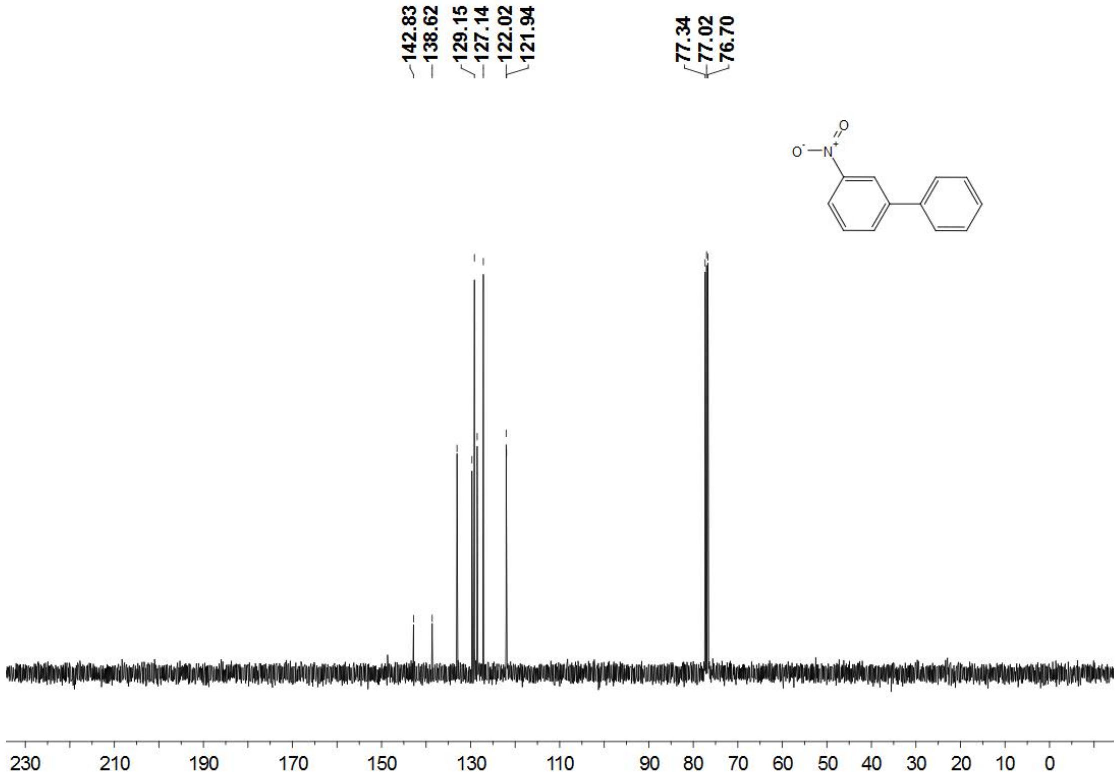
142.83
138.62
129.15
127.14
122.02
121.94
77.34
77.02
76.70

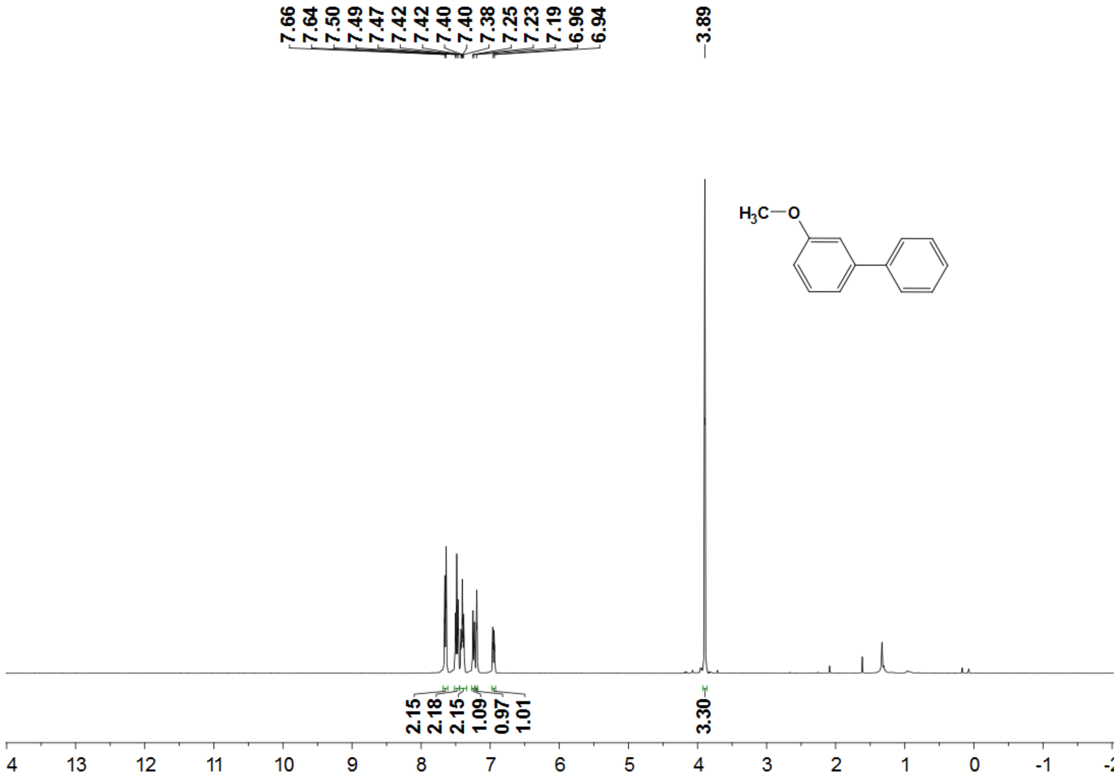
7.66
7.64
7.50
7.49
7.47
7.42
7.42
7.40
7.40
7.38
7.25
7.23
7.19
6.96
6.94
3.89
$H_3C-O$
2.15
2.18
2.15
1.09
0.97
1.01
3.30
4
13
12
11
10
9
8
7
6
5
4
3
2
1
0
-1

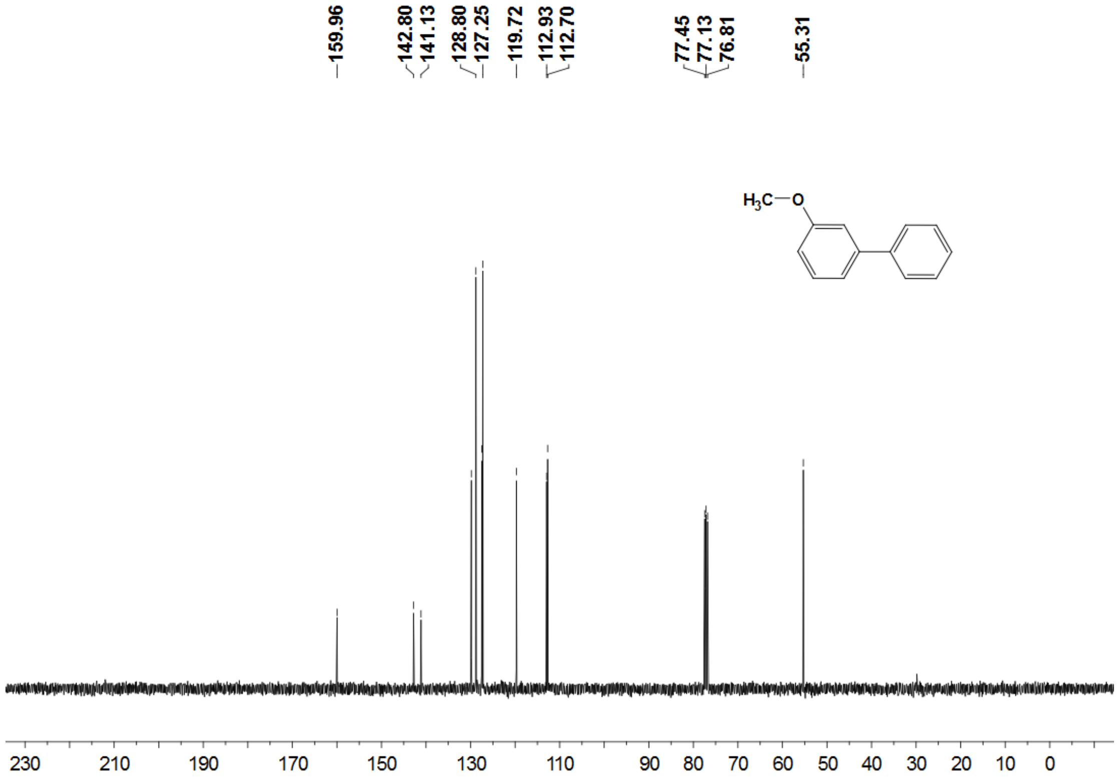
159.96
142.80
141.13
128.80
127.25
119.72
112.93
112.70
77.45
77.13
76.81
55.31
$H_3C-O$
230
210
190
170
150
130
110
90
80
70
60
50
40
30
20
10
0

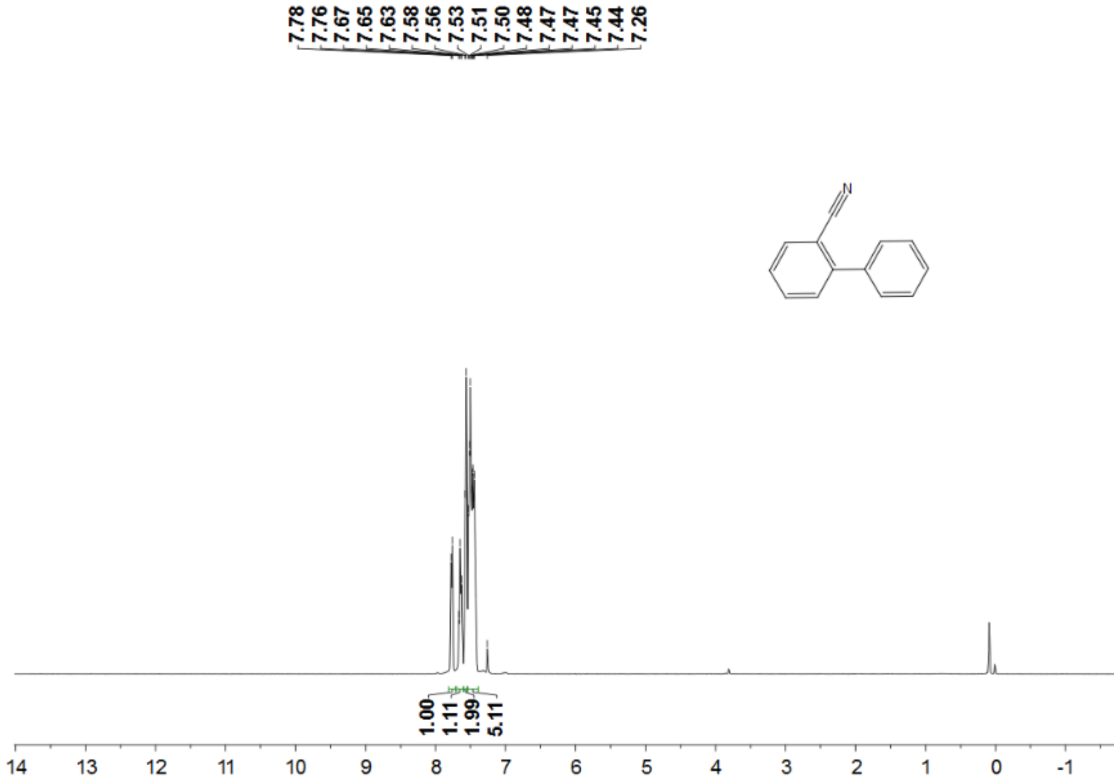
7.78
7.76
7.67
7.65
7.63
7.58
7.56
7.53
7.51
7.50
7.48
7.47
7.47
7.45
7.44
7.26
N
1.00
1.11
1.99
5.11
14 13 12 11 10 9 8 7 6 5 4 3 2 1 0 -1

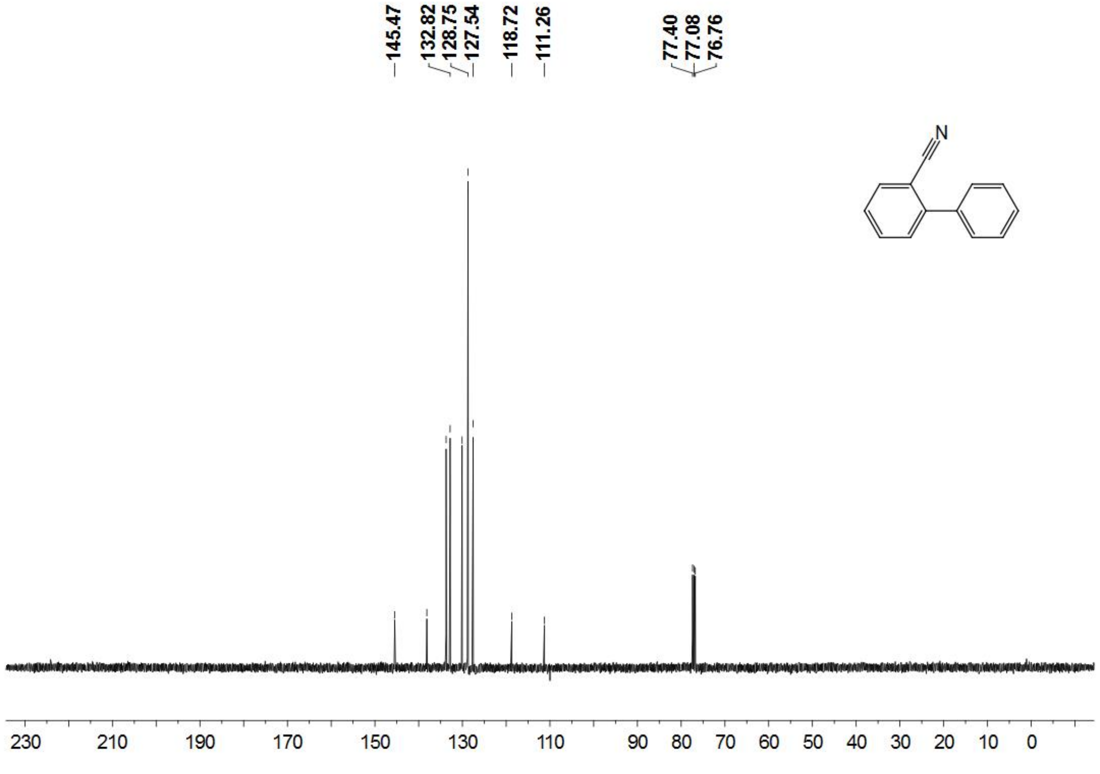
145.47
132.82
128.75
127.54
118.72
111.26
77.40
77.08
76.76
N
230 210 190 170 150 130 110 90 80 70 60 50 40 30 20 10 0

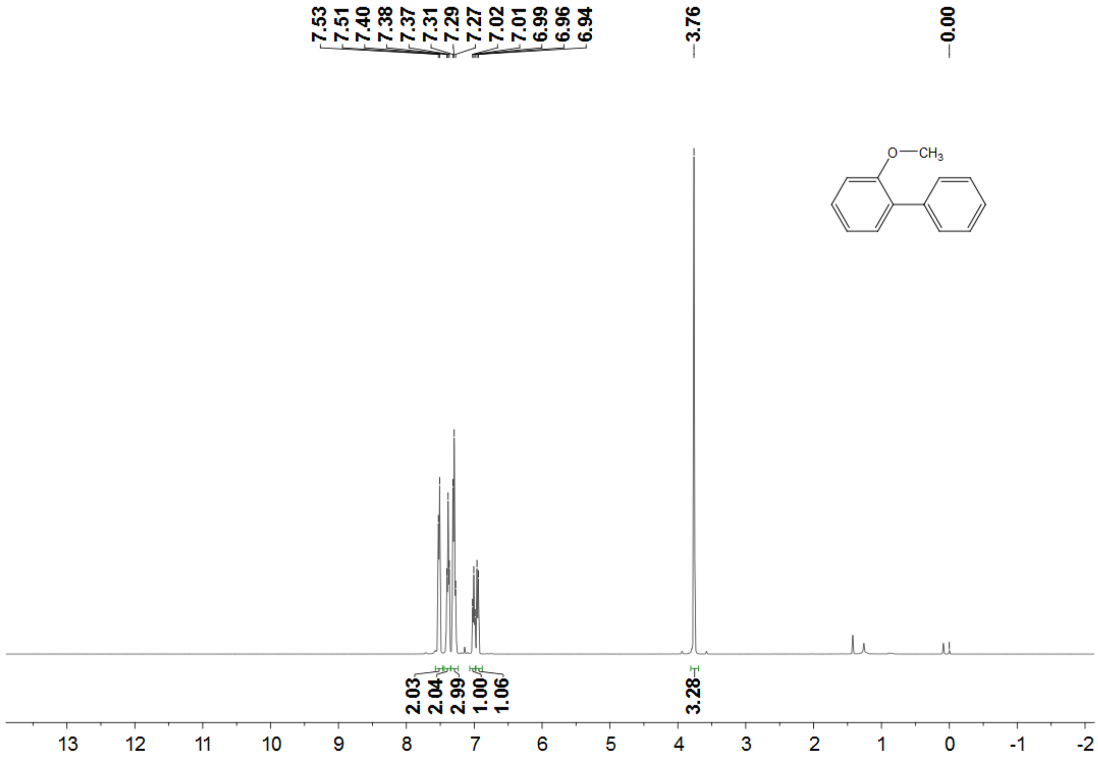
7.53
7.51
7.40
7.38
7.37
7.31
7.29
7.27
7.02
7.01
6.99
6.96
6.94
3.76
0.00
O—CH3
2.03
2.04
2.99
1.00
1.06
3.28
13
12
11
10
9
8
7
6
5
4
3
2
1
0
-1
-2

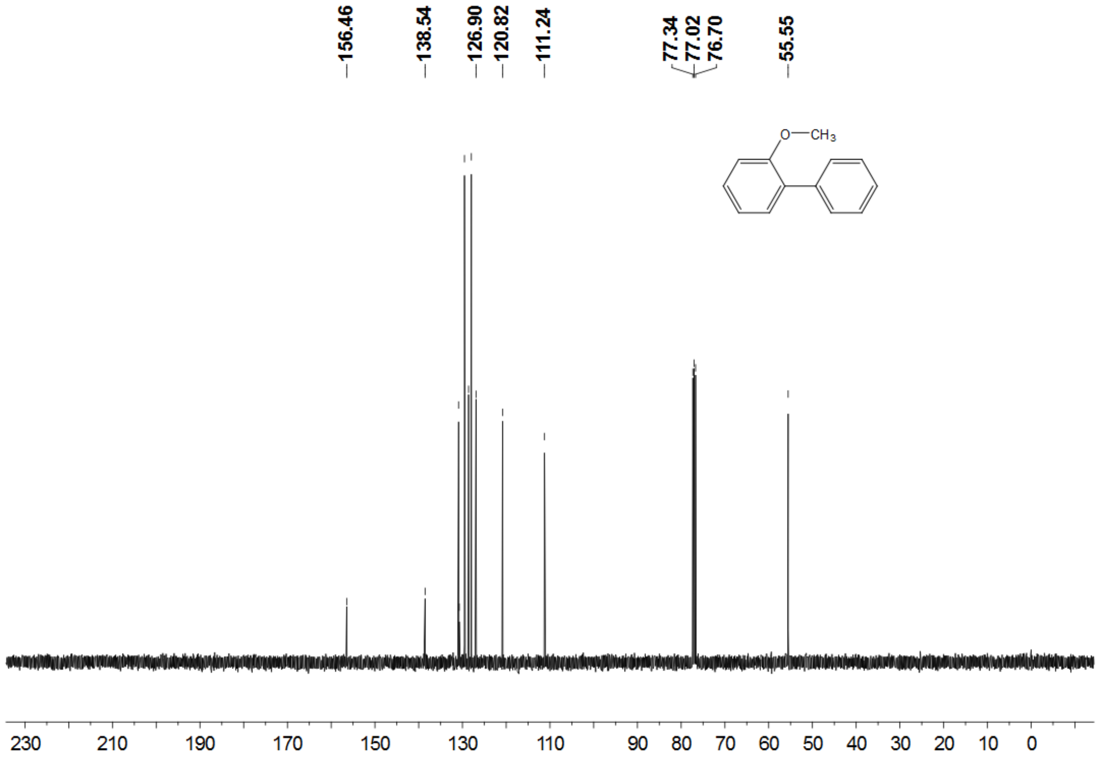
156.46
138.54
126.90
120.82
111.24
77.34
77.02
76.70
55.55
O—CH3
230
210
190
170
150
130
110
90
80
70
60
50
40
30
20
10
0

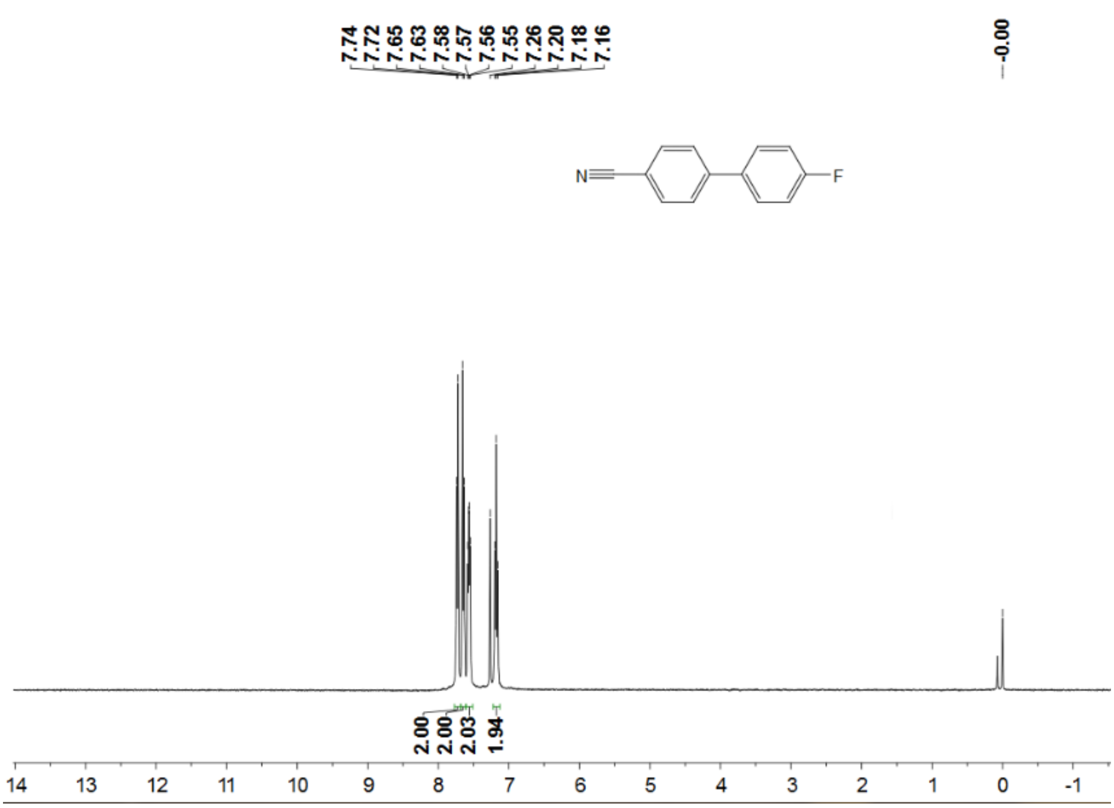
7.74
7.72
7.65
7.63
7.58
7.57
7.56
7.55
7.26
7.20
7.18
7.16
-0.00
N
F
2.00
2.00
2.03
1.94
14
13
12
11
10
9
8
7
6
5
4
3
2
1
0
-1

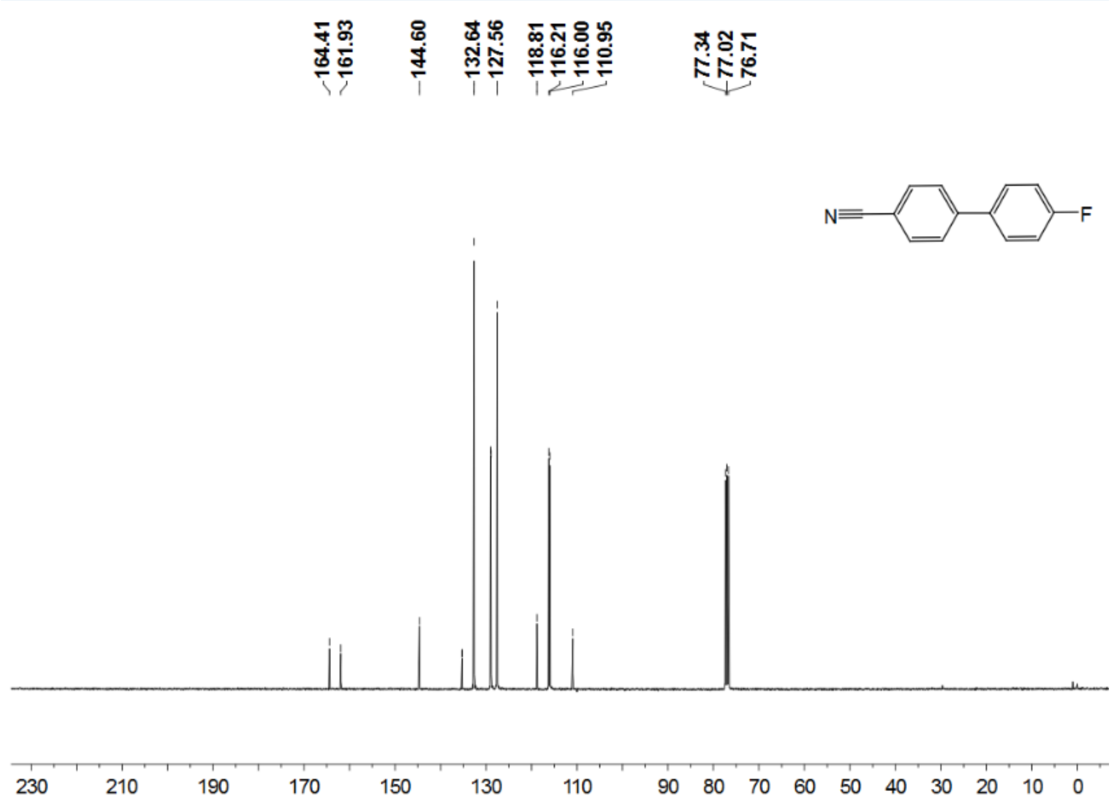
164.41
161.93
144.60
132.64
127.56
118.81
116.21
116.00
110.95
77.34
77.02
76.71
N
F
230
210
190
170
150
130
110
90
80
70
60
50
40
30
20
10
0

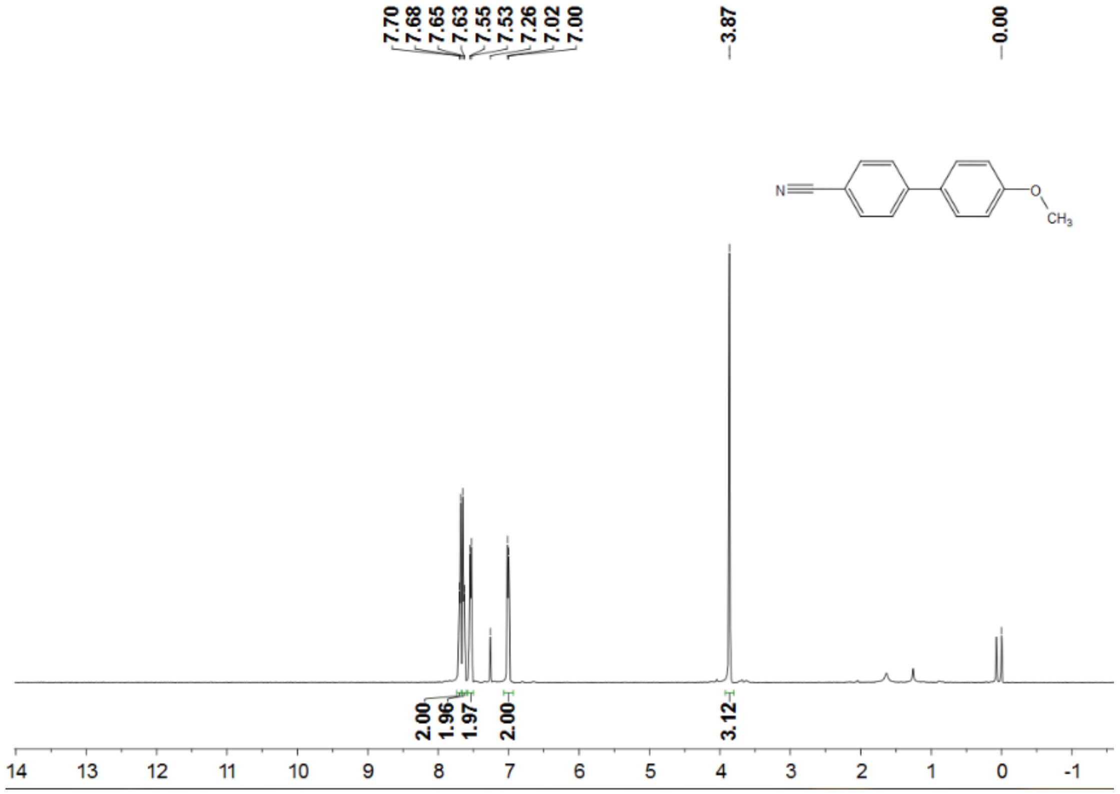
7.70
7.68
7.65
7.63
7.55
7.53
7.26
7.02
7.00
3.87
0.00
N
O
CH3
2.00
1.96
1.97
2.00
3.12
14 13 12 11 10 9 8 7 6 5 4 3 2 1 0 -1

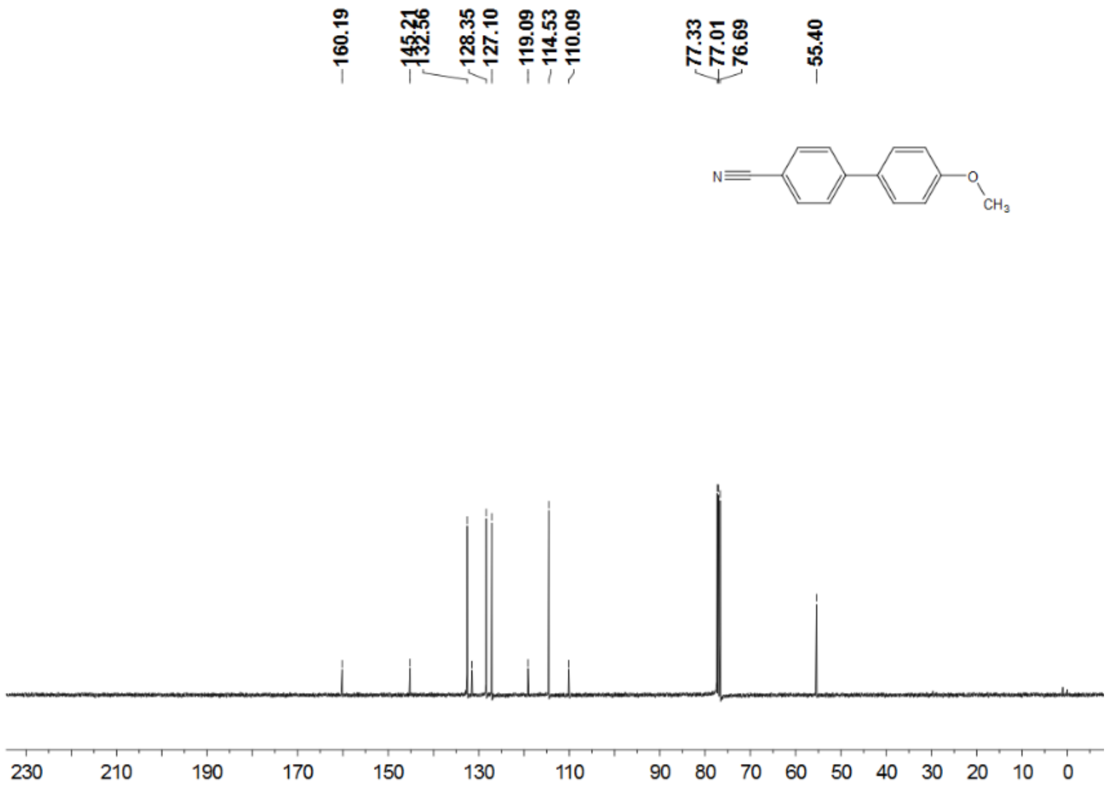
160.19
145.21
132.56
128.35
127.10
119.09
114.53
110.09
77.33
77.01
76.69
55.40
N
O
CH3
230 210 190 170 150 130 110 90 80 70 60 50 40 30 20 10 0

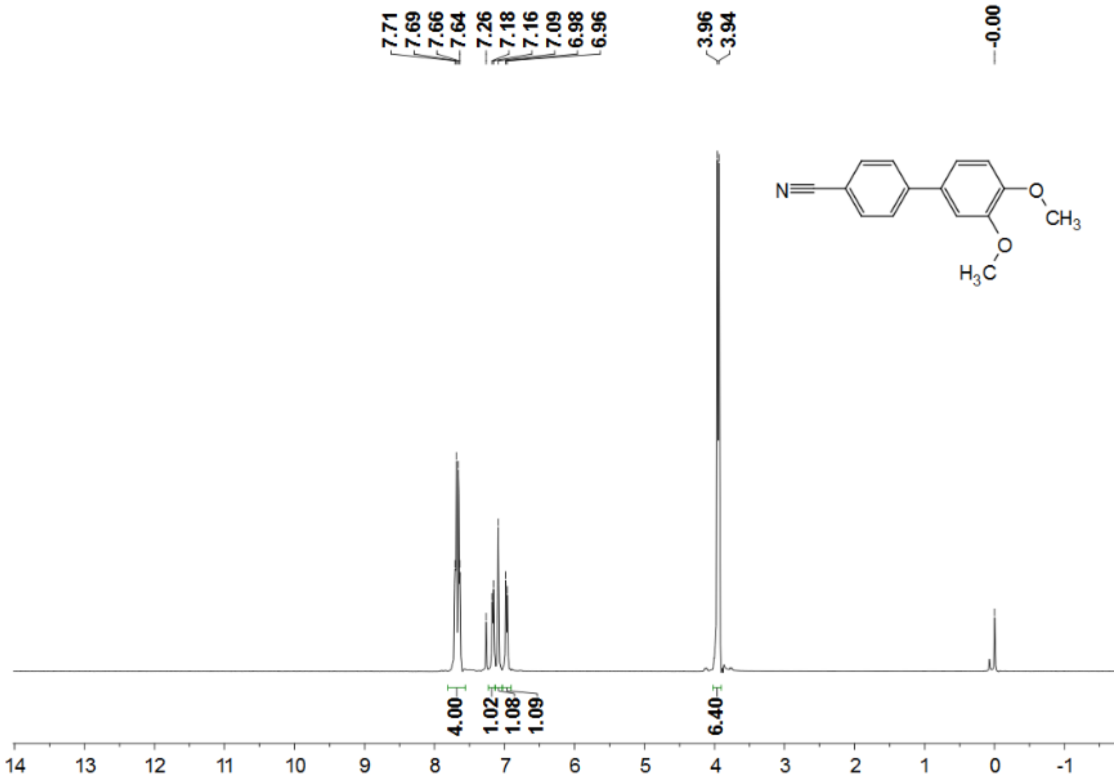
7.71
7.69
7.66
7.64
7.26
7.18
7.16
7.09
6.98
6.96
3.96
3.94
-0.00
N
O
CH3
O
H3C
4.00
1.02
1.08
1.09
6.40
14 13 12 11 10 9 8 7 6 5 4 3 2 1 0 -1

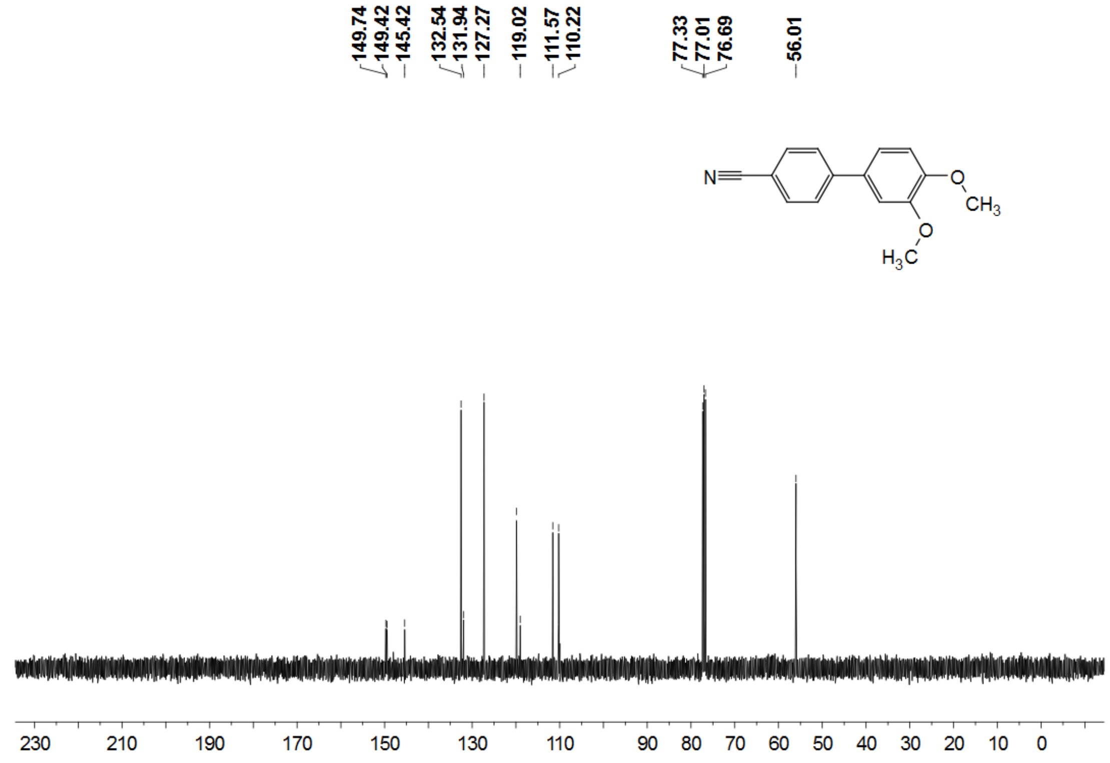
149.74
149.42
145.42
132.54
131.94
127.27
119.02
111.57
110.22
77.33
77.01
76.69
56.01
N
O
CH3
O
H3C
230 210 190 170 150 130 110 90 80 70 60 50 40 30 20 10 0

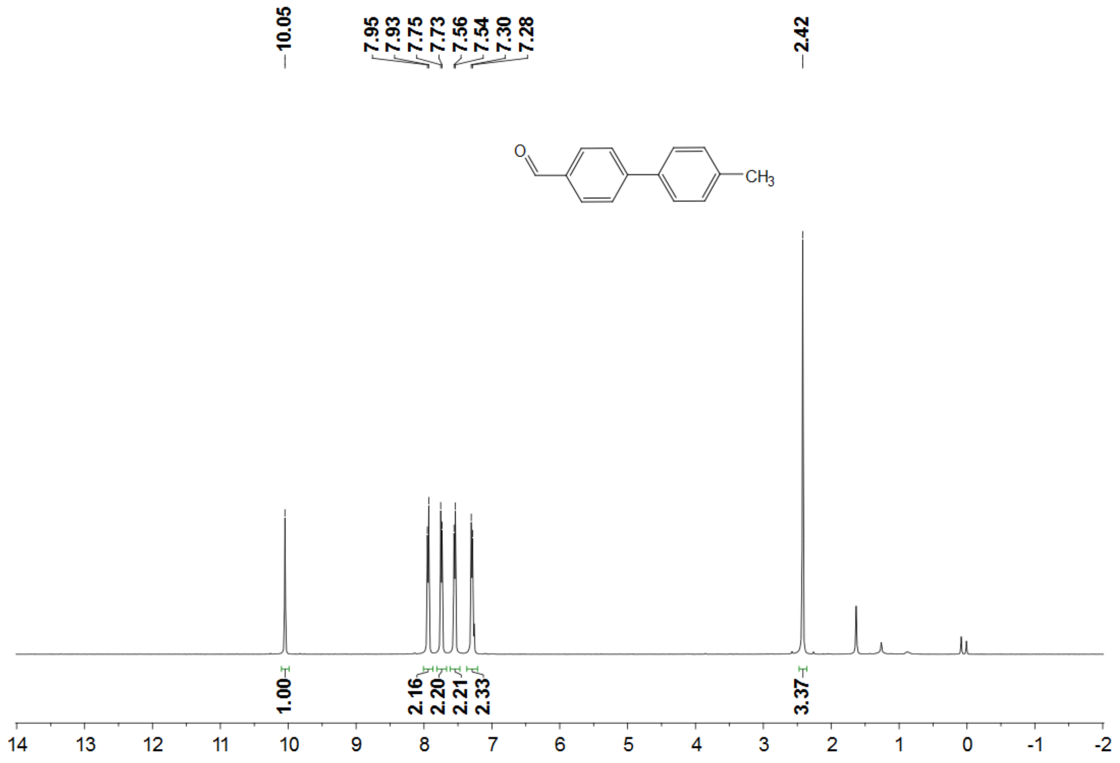
10.05
7.95
7.93
7.75
7.73
7.56
7.54
7.30
7.28
2.42
CH3
1.00
2.16
2.20
2.21
2.33
3.37
14 13 12 11 10 9 8 7 6 5 4 3 2 1 0 -1 -2

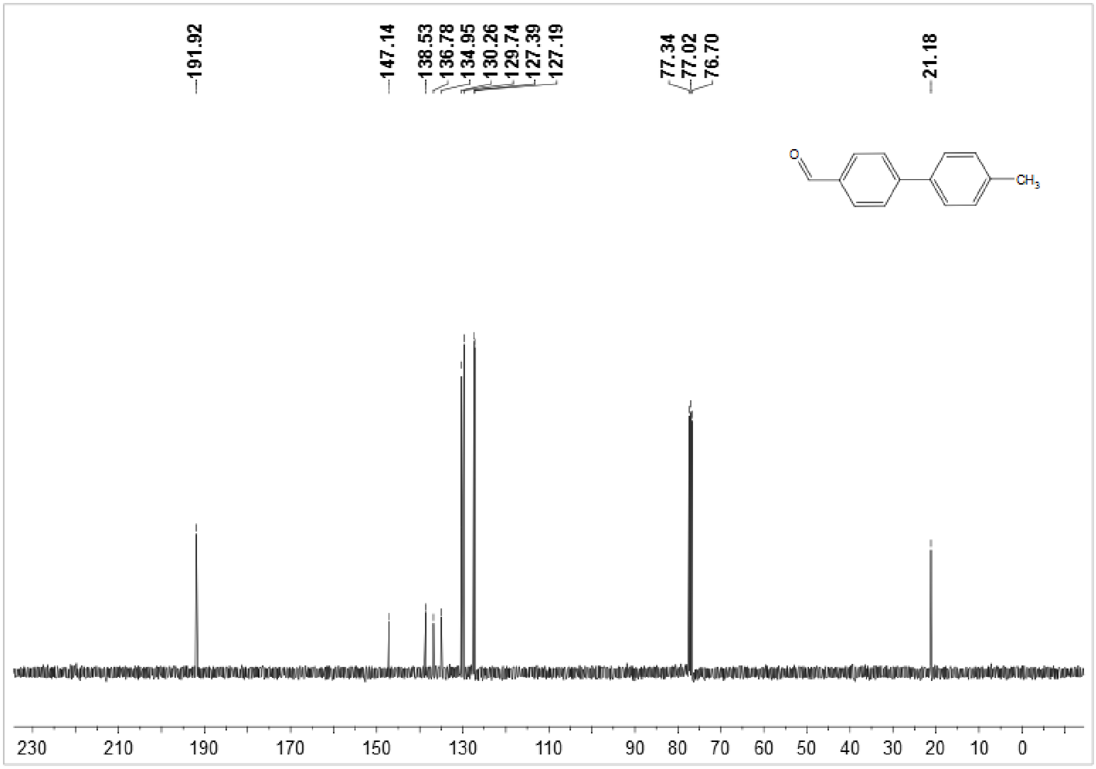
191.92
147.14
138.53
136.78
134.95
130.26
129.74
127.39
127.19
77.34
77.02
76.70
21.18
CH3
230 210 190 170 150 130 110 90 80 70 60 50 40 30 20 10 0

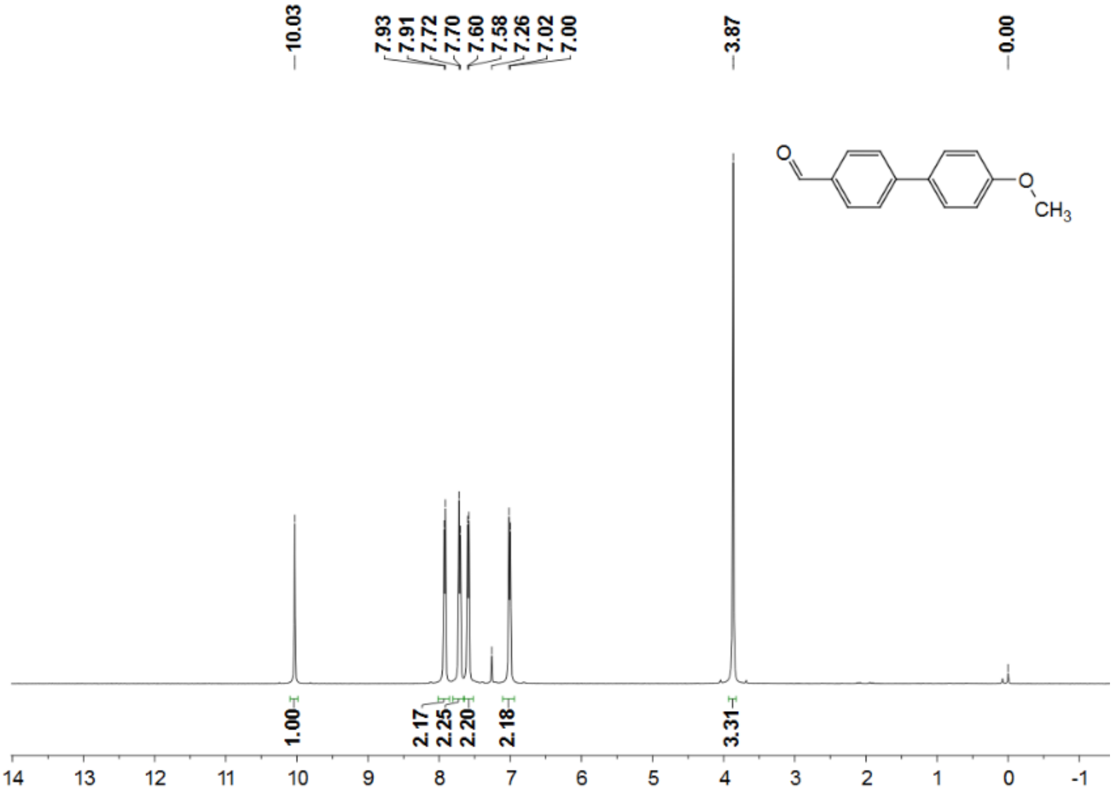
10.03
7.93
7.91
7.72
7.70
7.60
7.58
7.26
7.02
7.00
3.87
0.00
O
CH3
1.00
2.17
2.25
2.20
2.18
3.31
14 13 12 11 10 9 8 7 6 5 4 3 2 1 0 -1

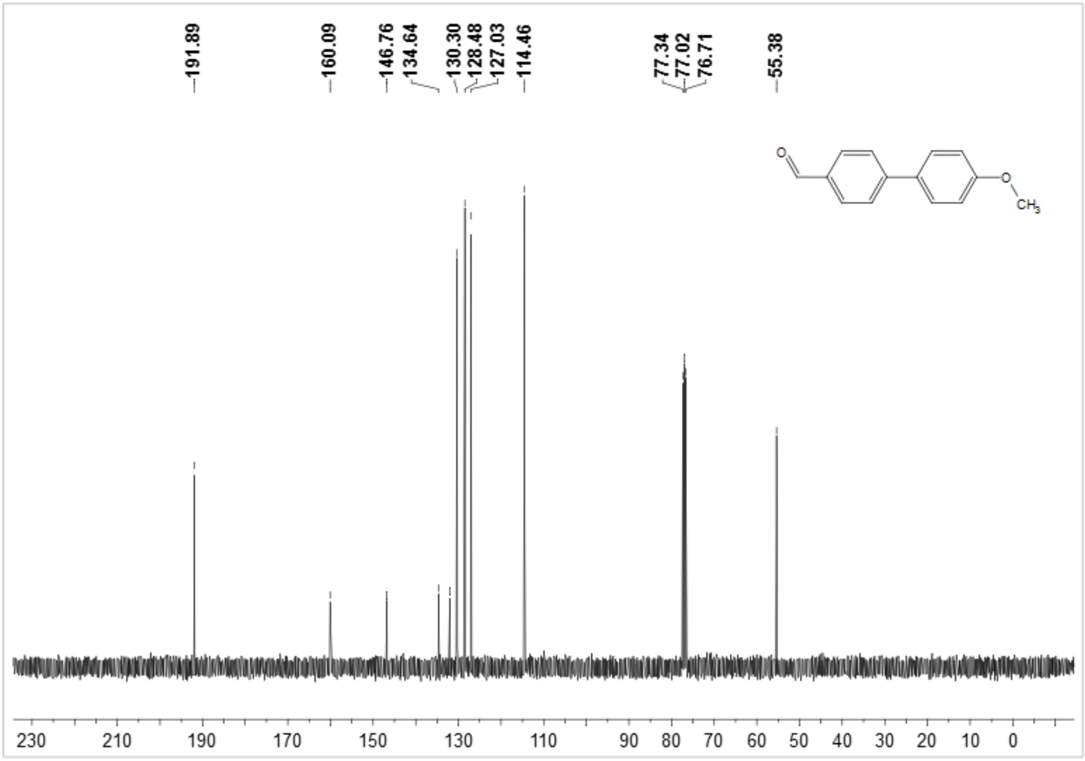
191.89
160.09
146.76
134.64
130.30
128.48
127.03
114.46
77.34
77.02
76.71
55.38
O
CH3
230 210 190 170 150 130 110 90 80 70 60 50 40 30 20 10 0

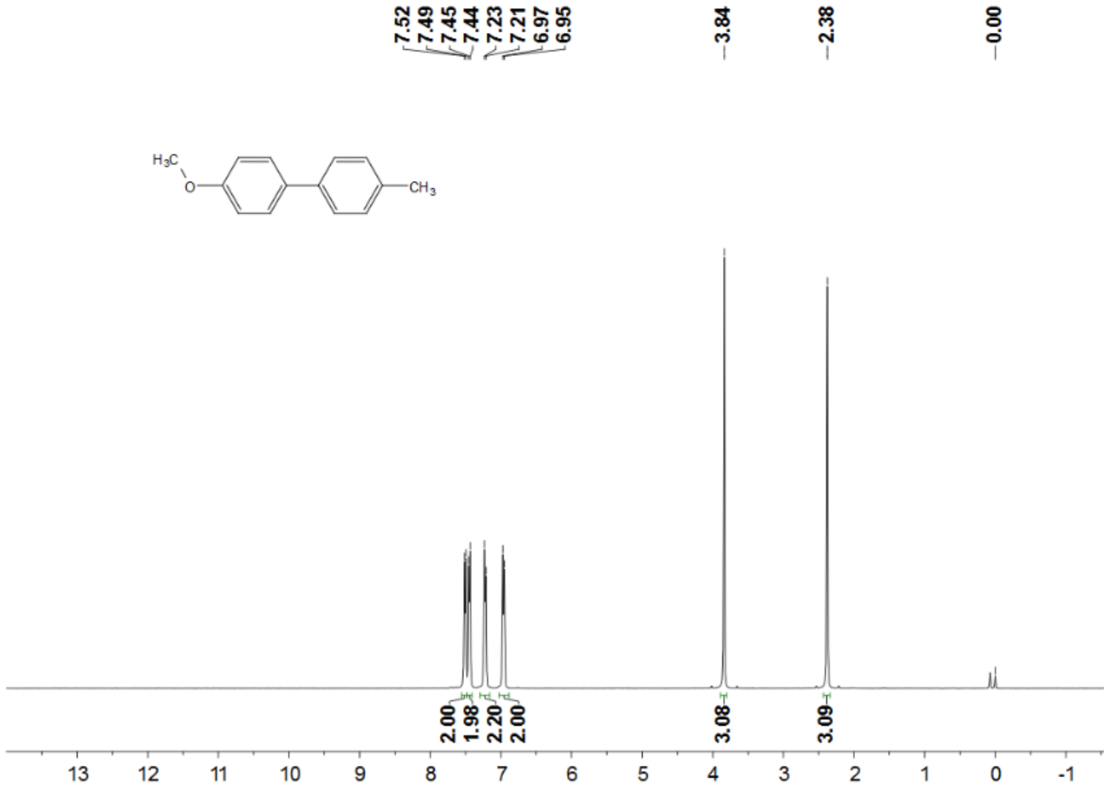
7.52
7.49
7.45
7.44
7.23
7.21
6.97
6.95
3.84
2.38
0.00
H3C
O
CH3
2.00
1.98
2.20
2.00
3.08
3.09
13 12 11 10 9 8 7 6 5 4 3 2 1 0 -1

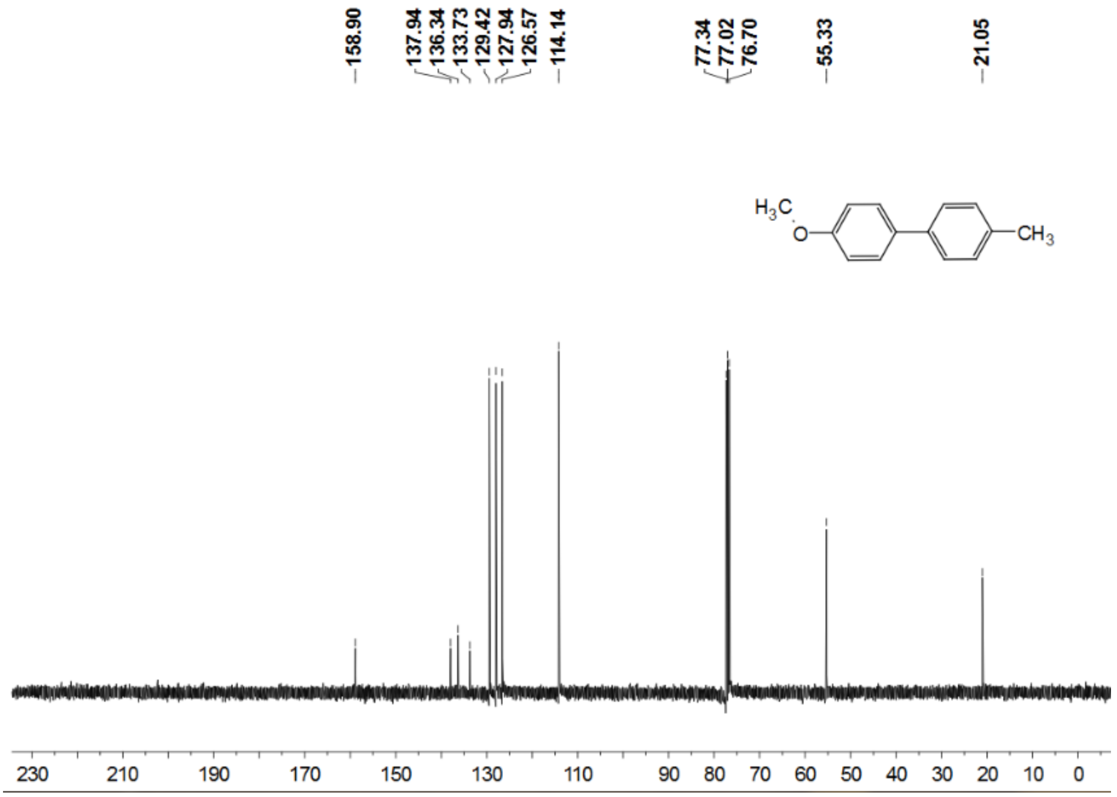
158.90
137.94
136.34
133.73
129.42
127.94
126.57
114.14
77.34
77.02
76.70
55.33
21.05
H3C
O
CH3
230 210 190 170 150 130 110 90 80 70 60 50 40 30 20 10 0

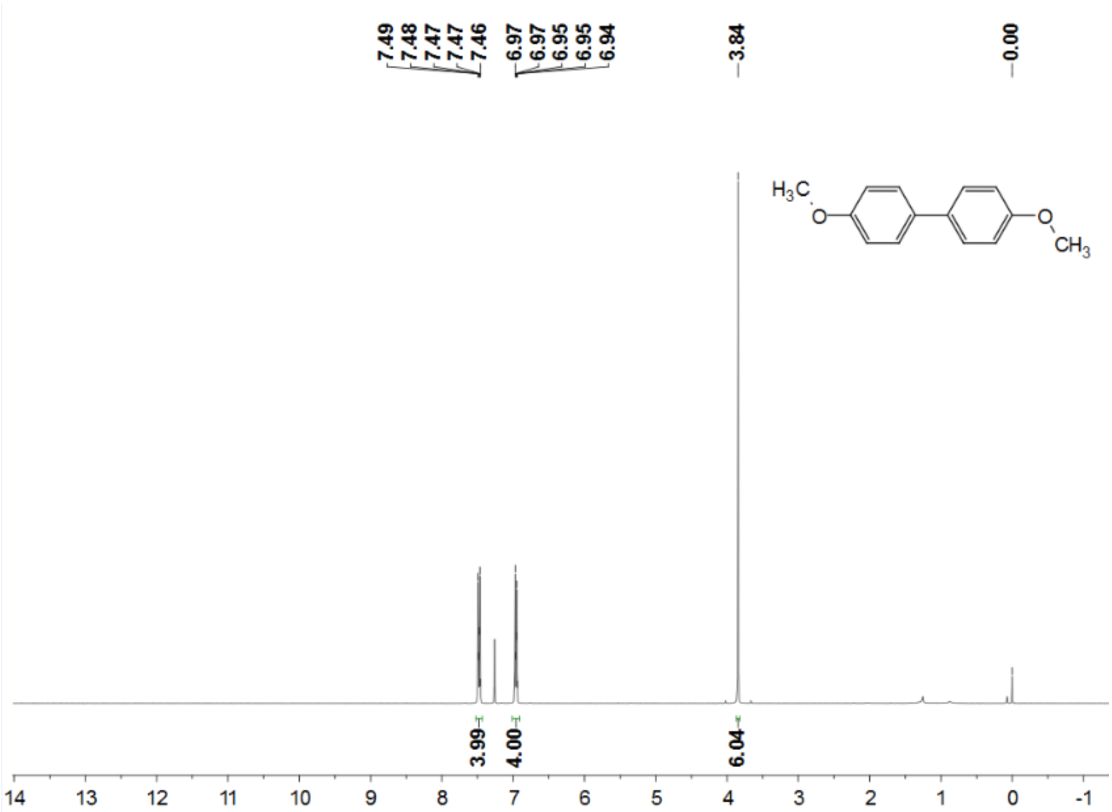
7.49
7.48
7.47
7.47
7.46
6.97
6.97
6.95
6.95
6.94
3.84
0.00
H3C
O
O
CH3
3.99
4.00
6.04
14 13 12 11 10 9 8 7 6 5 4 3 2 1 0 -1

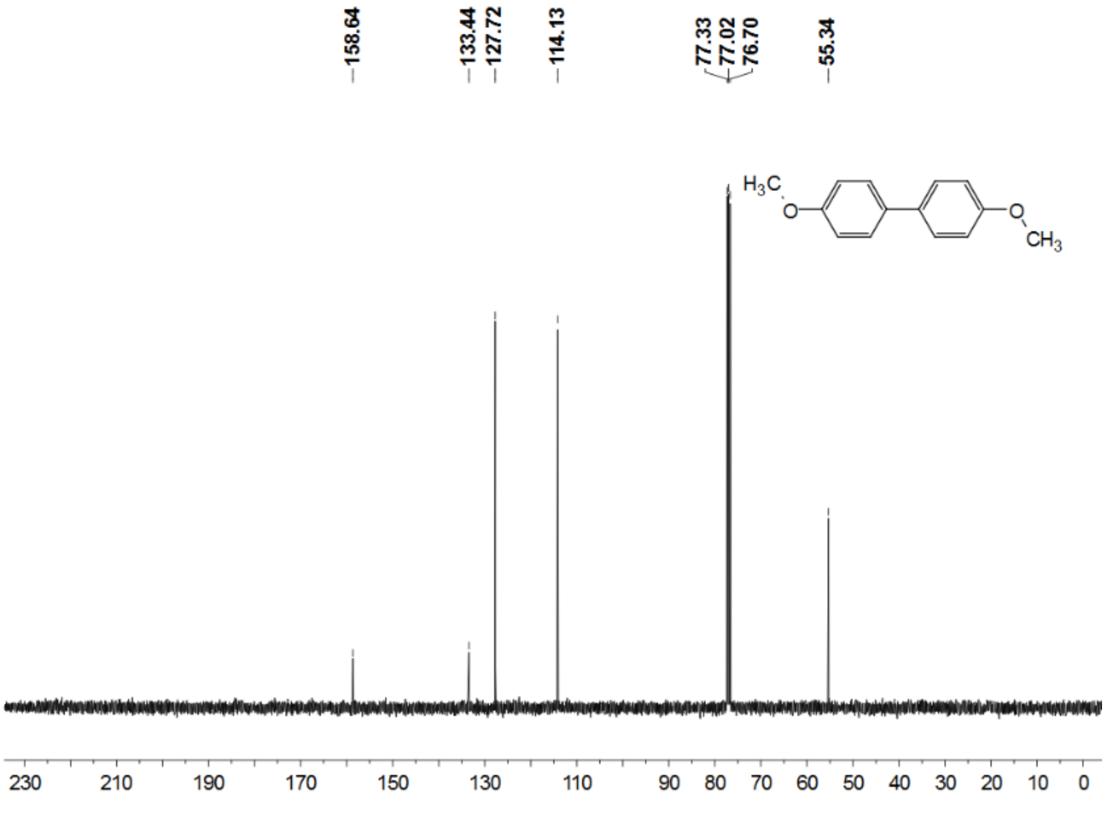
158.64
133.44
127.72
114.13
77.33
77.02
76.70
55.34
H3C
O
O
CH3
230 210 190 170 150 130 110 90 80 70 60 50 40 30 20 10 0

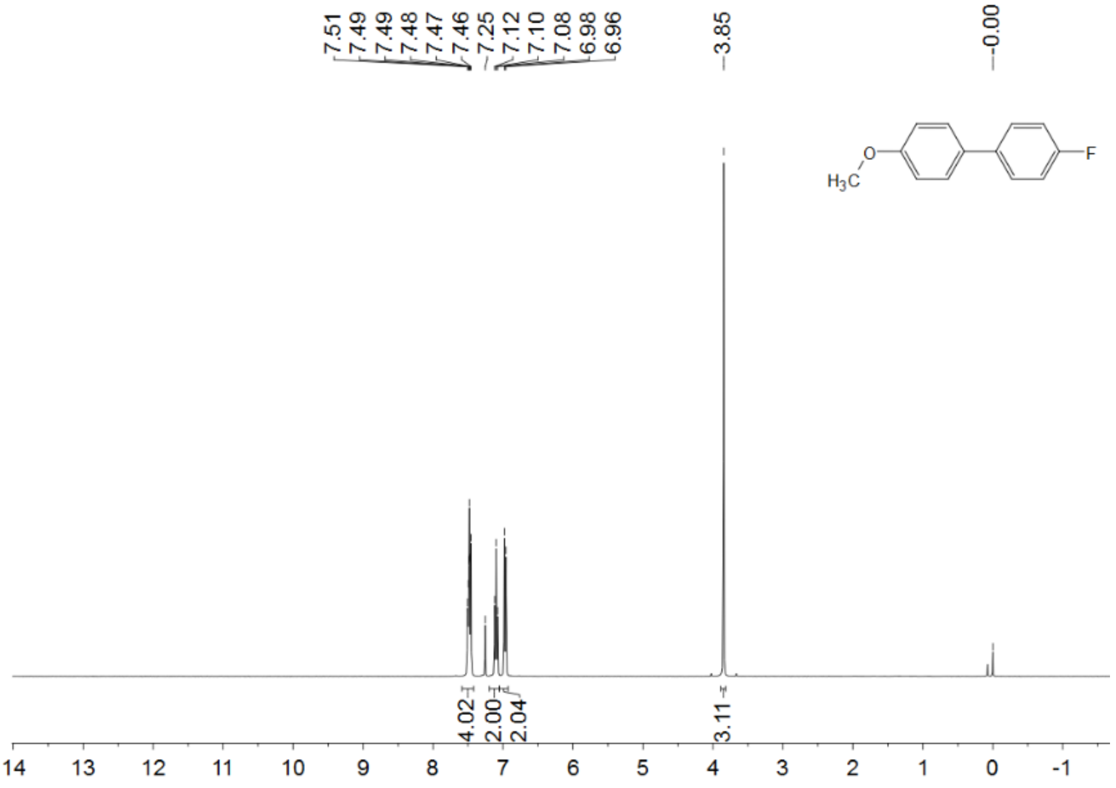

7.51
7.49
7.49
7.48
7.47
7.46
7.25
7.12
7.10
7.08
6.98
6.96
3.85
0.00
4.02
2.00
2.04
3.11
14 13 12 11 10 9 8 7 6 5 4 3 2 1 0 -1


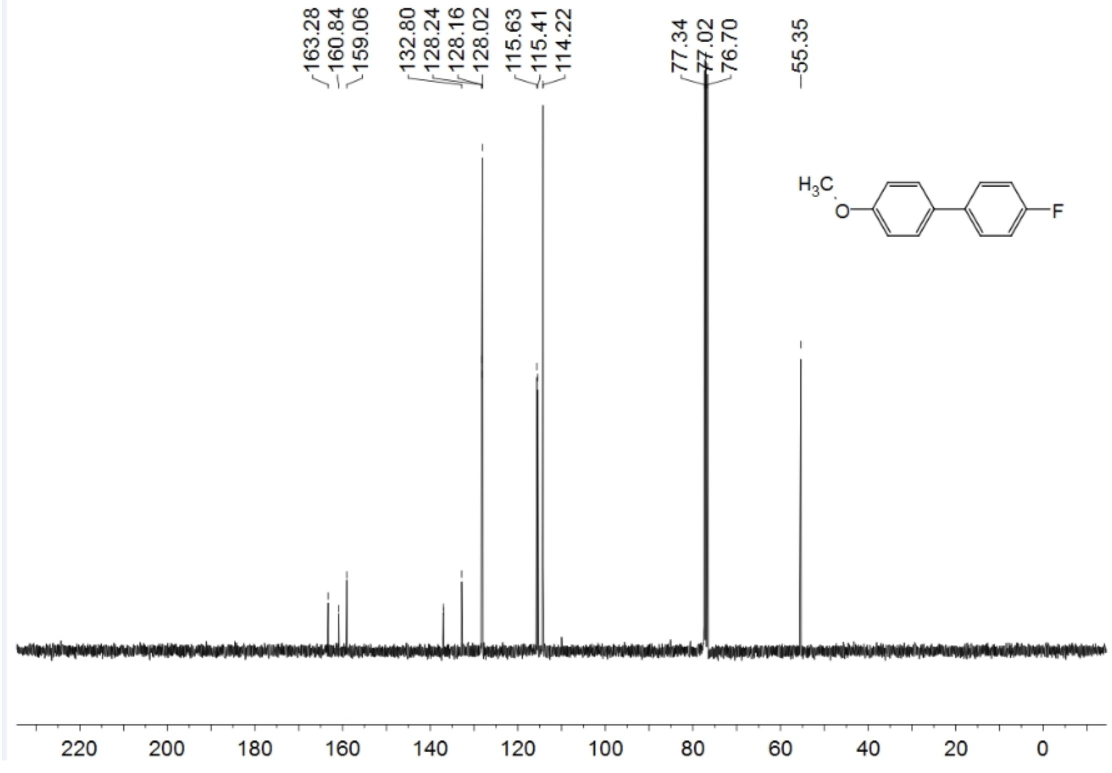

163.28
160.84
159.06
132.80
128.24
128.16
128.02
115.63
115.41
114.22
77.34
77.02
76.70
55.35
220 200 180 160 140 120 100 80 60 40 20 0

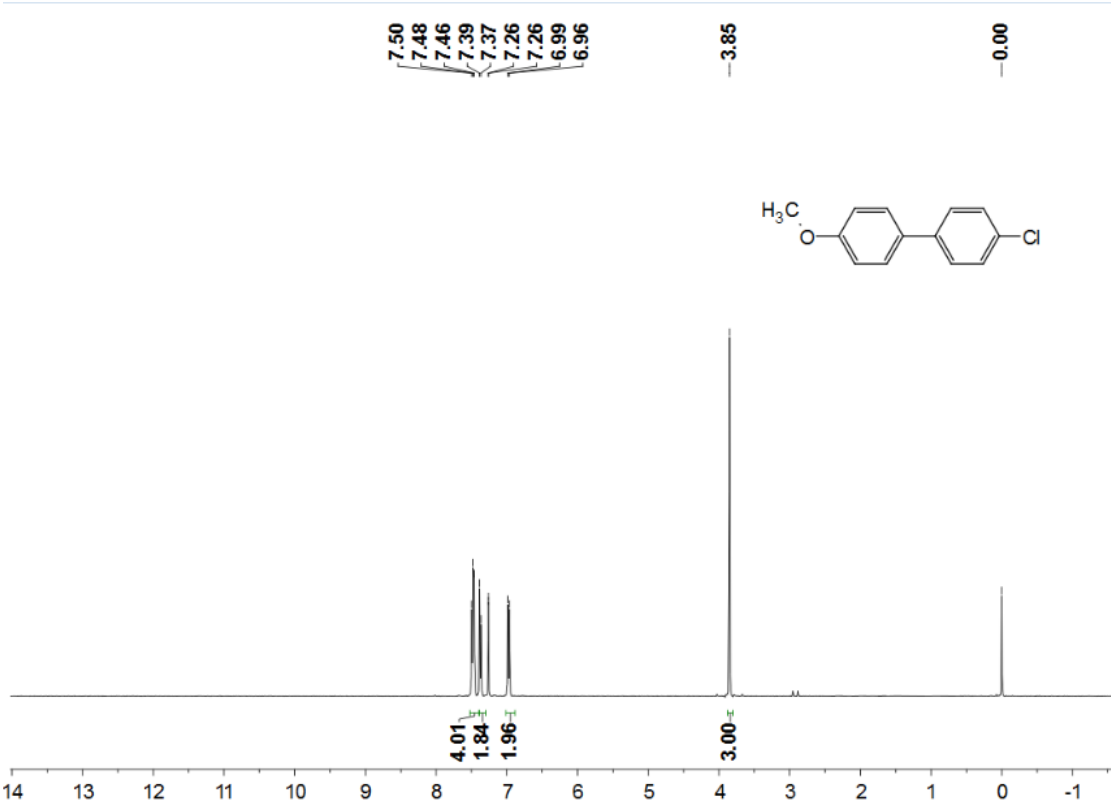
7.50
7.48
7.46
7.39
7.37
7.26
7.26
6.99
6.96
3.85
0.00
H3C
O
Cl
4.01
1.84
1.96
3.00
14 13 12 11 10 9 8 7 6 5 4 3 2 1 0 -1

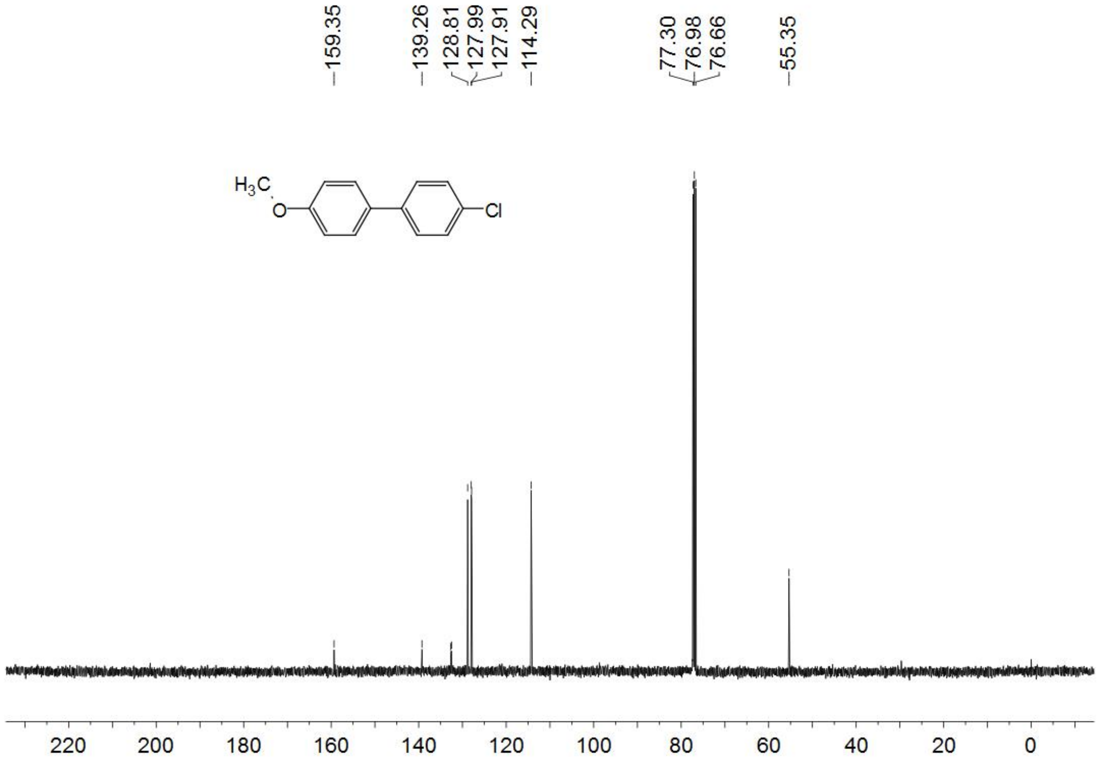
159.35
139.26
128.81
127.99
127.91
114.29
77.30
76.98
76.66
55.35
H3C
O
Cl
220 200 180 160 140 120 100 80 60 40 20 0

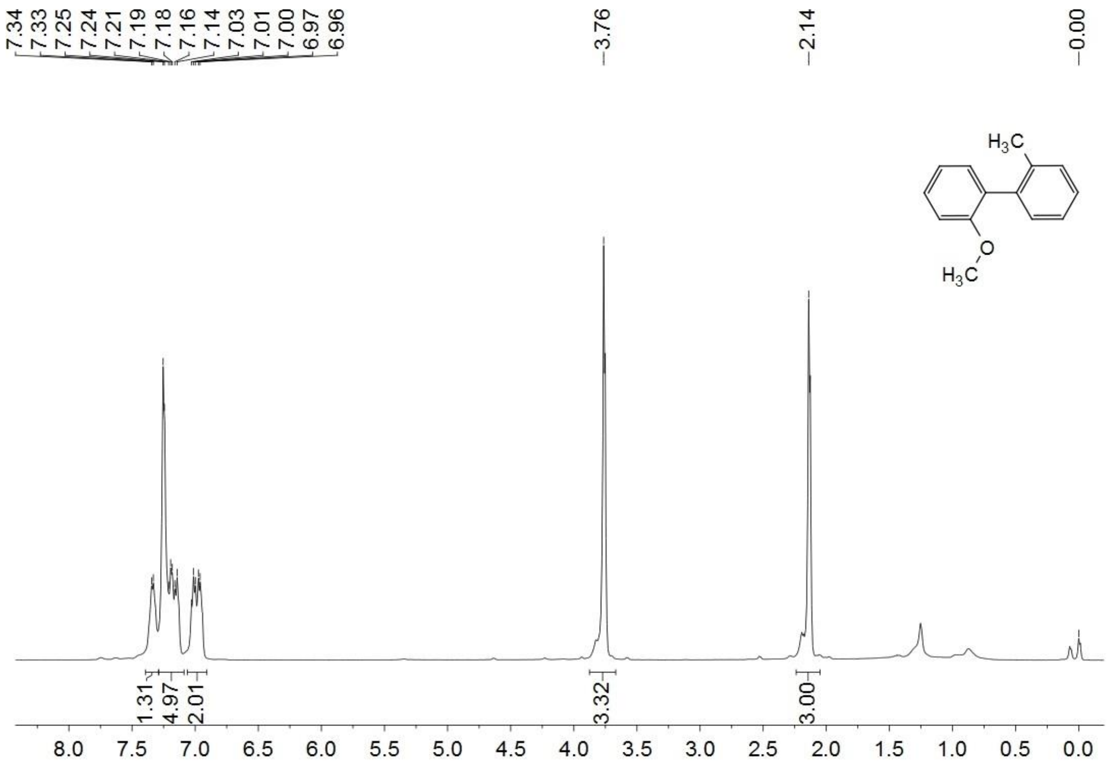

7.34
7.33
7.25
7.24
7.21
7.19
7.18
7.16
7.14
7.03
7.01
7.00
6.97
6.96
3.76
2.14
0.00
$H_3C$
O
$H_3C$
1.31
4.97
2.01
3.32
3.00
8.0 7.5 7.0 6.5 6.0 5.5 5.0 4.5 4.0 3.5 3.0 2.5 2.0 1.5 1.0 0.5 0.0


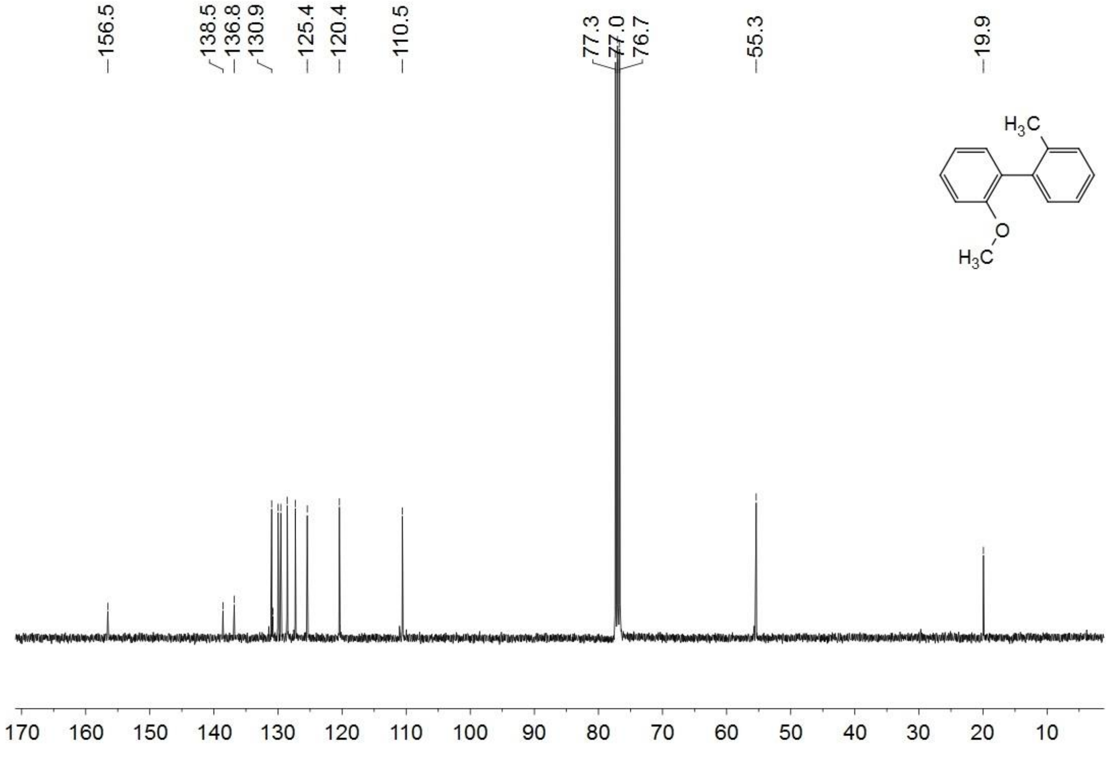

156.5
138.5
136.8
130.9
125.4
120.4
110.5
77.3
77.0
76.7
55.3
19.9
$H_3C$
O
$H_3C$
170 160 150 140 130 120 110 100 90 80 70 60 50 40 30 20 10

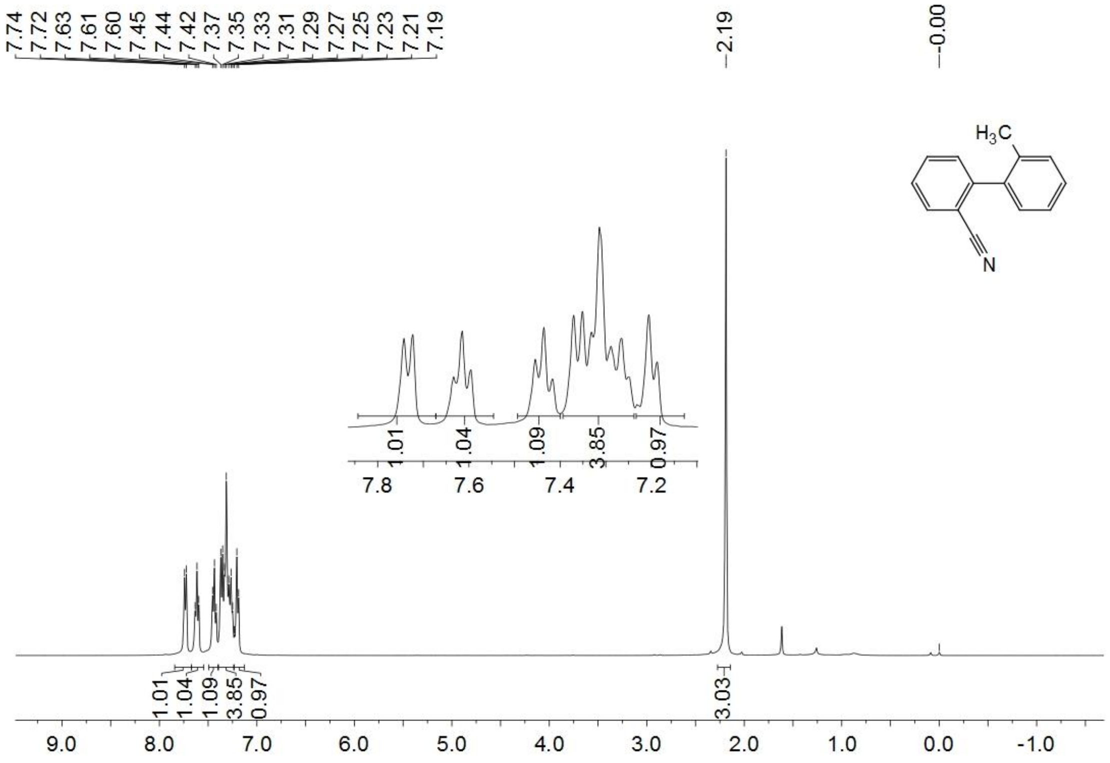

7.74
7.72
7.63
7.61
7.60
7.45
7.44
7.42
7.37
7.35
7.33
7.31
7.29
7.27
7.25
7.23
7.21
7.19
2.19
0.00
$H_3C$
N
1.01
1.04
1.09
3.85
0.97
7.8
7.6
7.4
7.2
3.03
9.0
8.0
7.0
6.0
5.0
4.0
3.0
2.0
1.0
0.0
-1.0


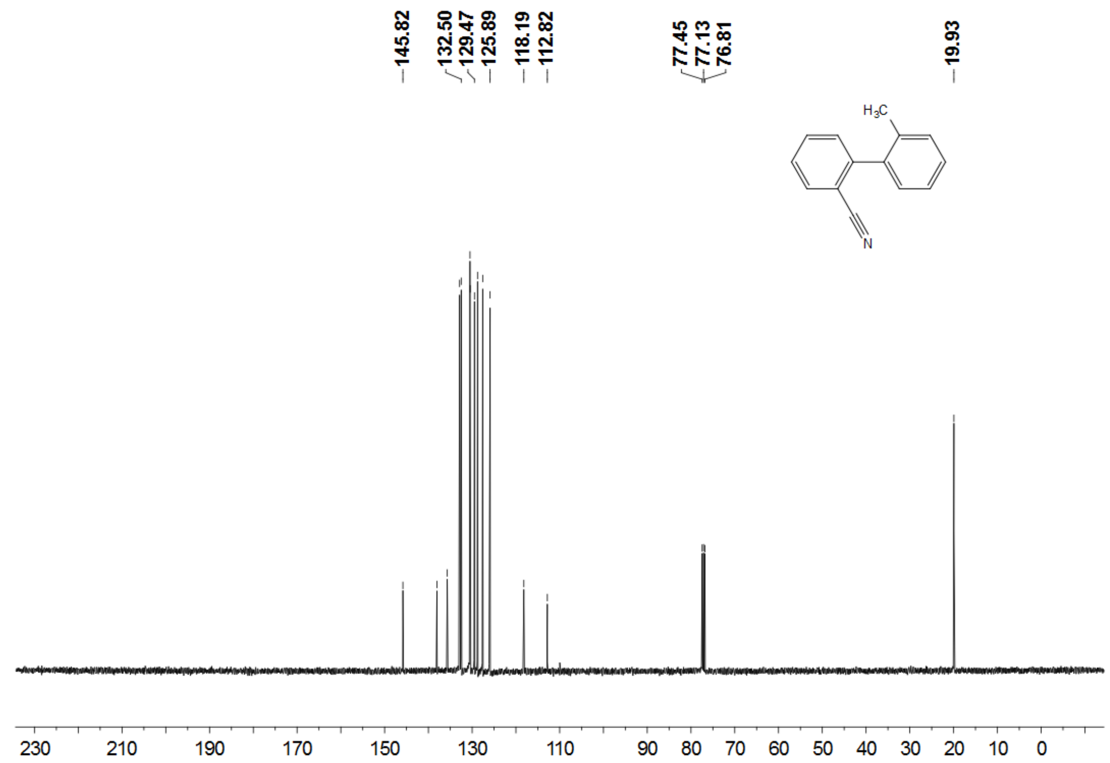

145.82
132.50
129.47
125.89
118.19
112.82
77.45
77.13
76.81
19.93
$H_3C$
N
230
210
190
170
150
130
110
90
80
70
60
50
40
30
20
10
0

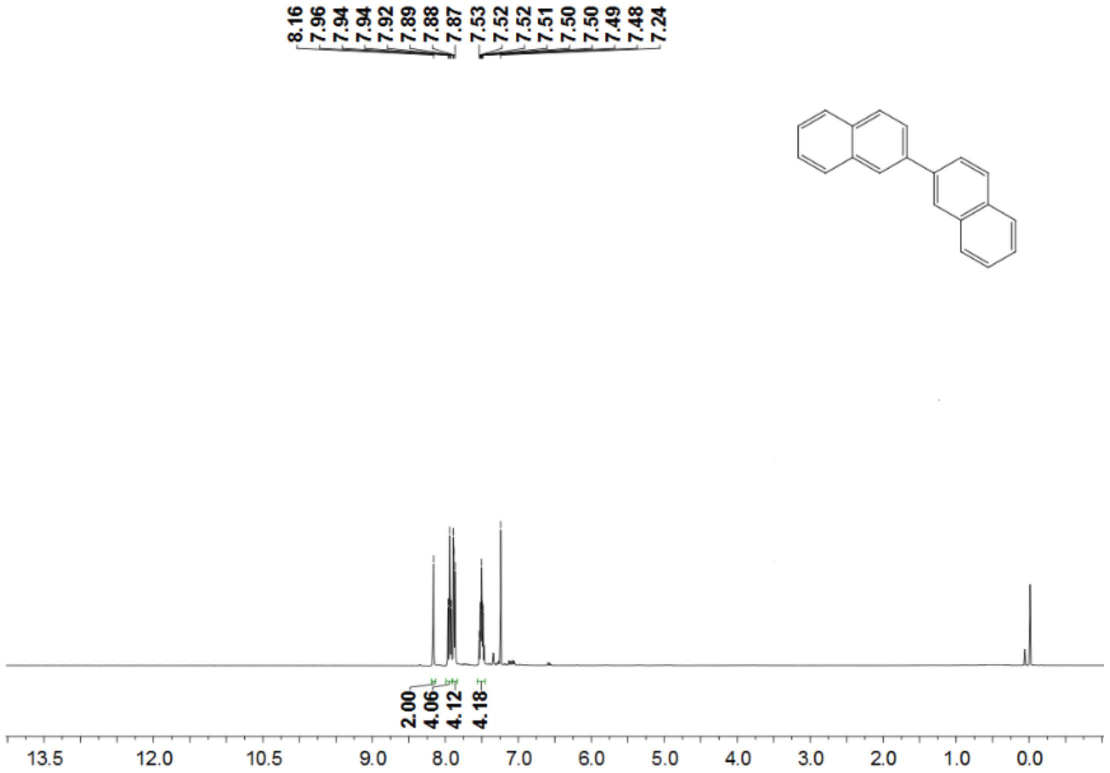
8.16
7.96
7.94
7.94
7.92
7.89
7.88
7.87
7.53
7.52
7.52
7.51
7.50
7.50
7.49
7.48
7.24
2.00
4.06
4.12
4.18
13.5
12.0
10.5
9.0
8.0
7.0
6.0
5.0
4.0
3.0
2.0
1.0
0.0

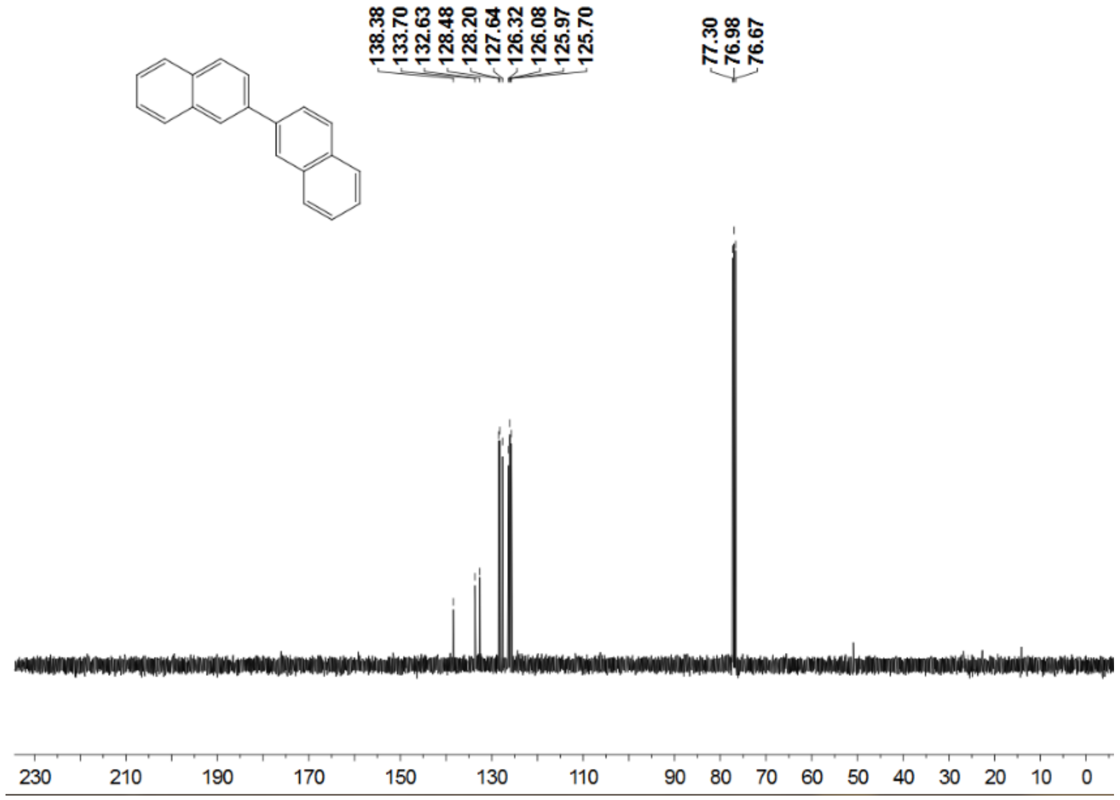
138.38
133.70
132.63
128.48
128.20
127.64
126.32
126.08
125.97
125.70
77.30
76.98
76.67
230
210
190
170
150
130
110
90
80
70
60
50
40
30
20
10
0

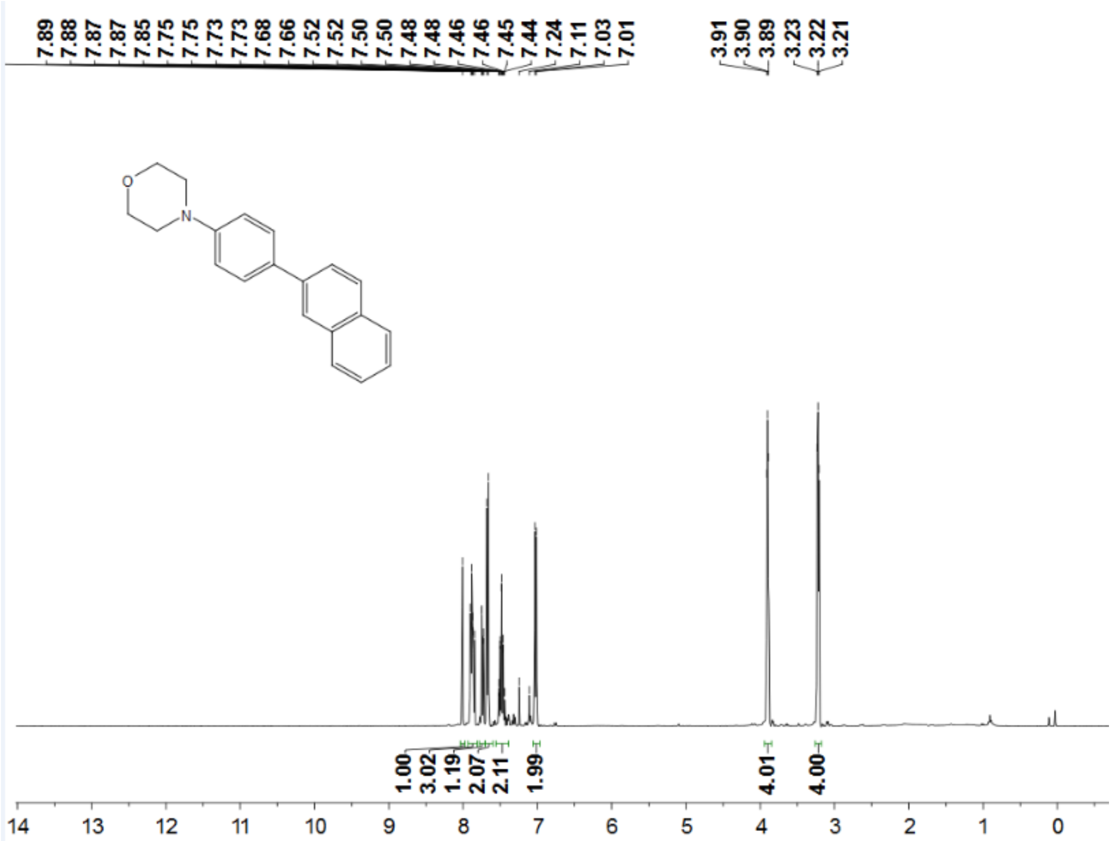
7.89 7.88 7.87 7.87 7.85 7.75 7.73 7.73 7.68 7.66 7.52 7.52 7.50 7.50 7.48 7.48 7.46 7.46 7.45 7.44 7.24 7.11 7.03 7.01
3.91 3.90 3.89 3.23 3.22 3.21
O
N
1.00 3.02 1.19 2.07 2.11 1.99
4.01
4.00
14 13 12 11 10 9 8 7 6 5 4 3 2 1 0

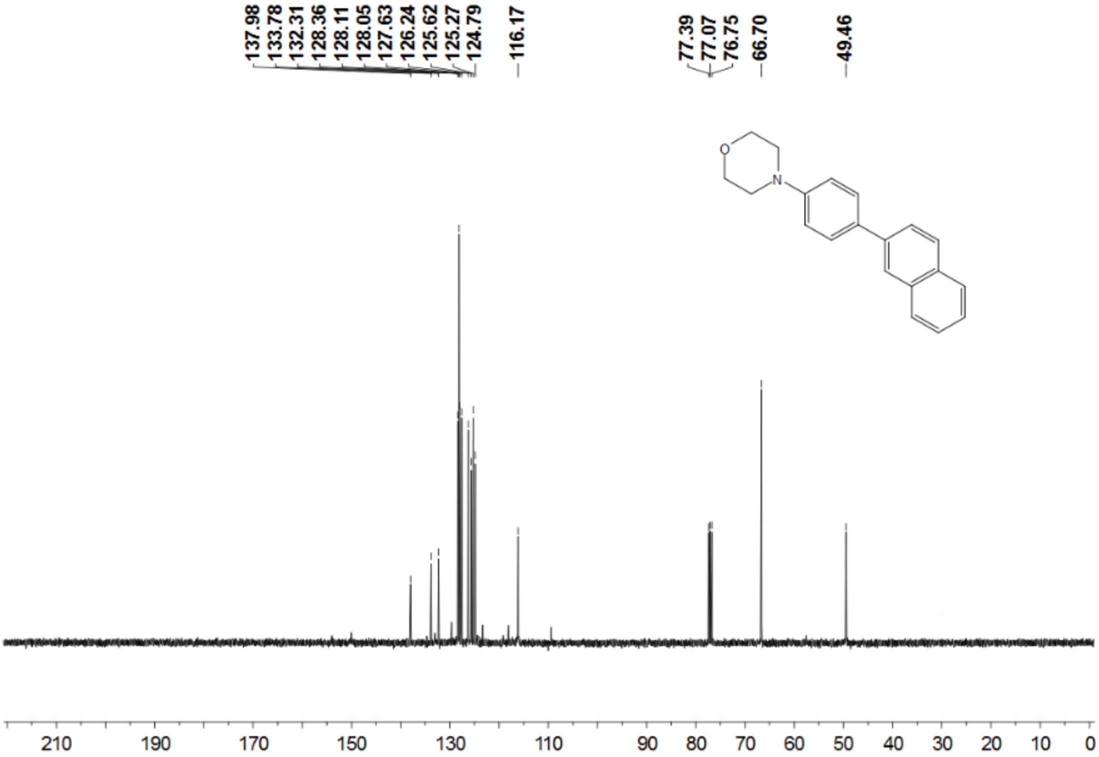
137.98 133.78 132.31 128.36 128.11 128.05 127.63 126.24 125.62 125.27 124.79
116.17
77.39 77.07 76.75
66.70
49.46
O
N
210 190 170 150 130 110 90 80 70 60 50 40 30 20 10 0

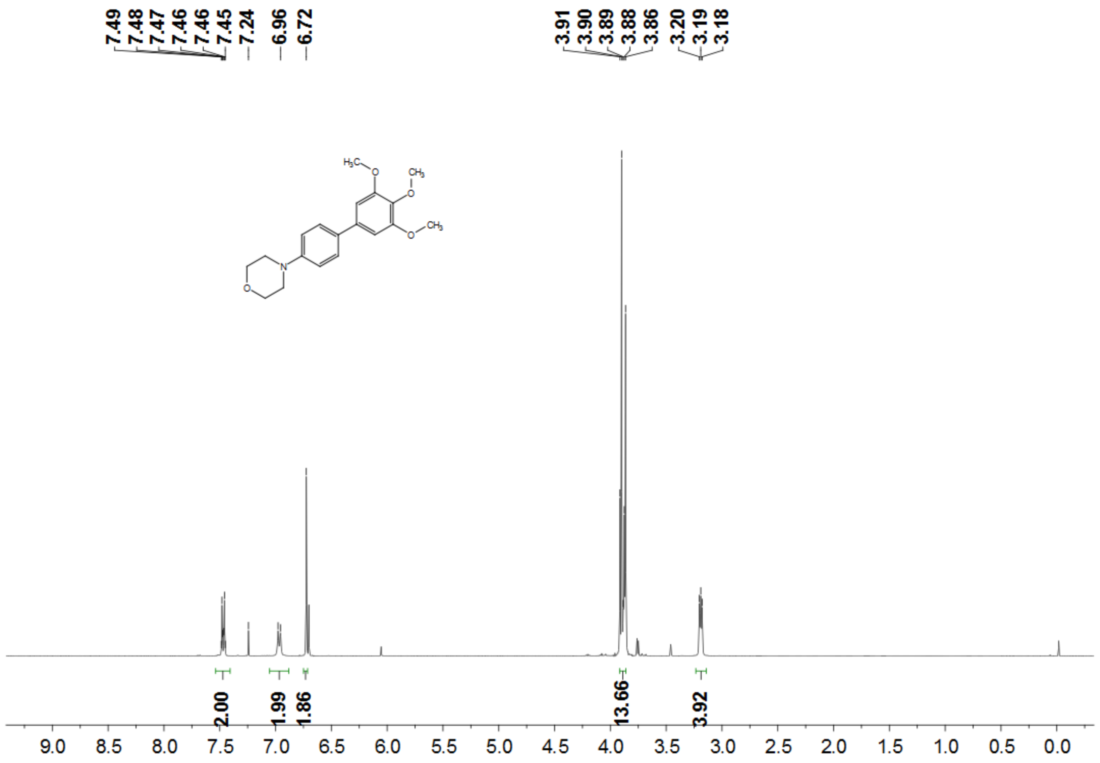
7.49
7.48
7.47
7.46
7.46
7.45
7.24
6.96
6.72
3.91
3.90
3.89
3.88
3.86
3.20
3.19
3.18
2.00
1.99
1.86
13.66
3.92
9.0 8.5 8.0 7.5 7.0 6.5 6.0 5.5 5.0 4.5 4.0 3.5 3.0 2.5 2.0 1.5 1.0 0.5 0.0

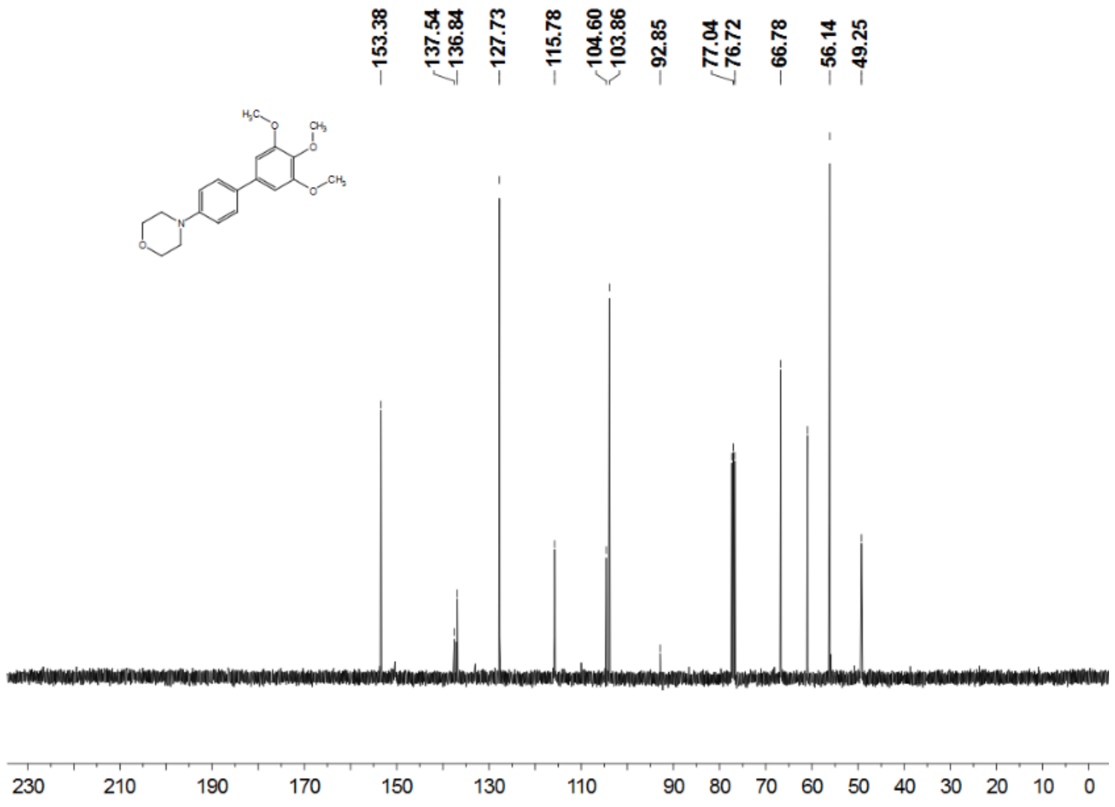
153.38
137.54
136.84
127.73
115.78
104.60
103.86
92.85
77.04
76.72
66.78
56.14
49.25
230 210 190 170 150 130 110 90 80 70 60 50 40 30 20 10 0

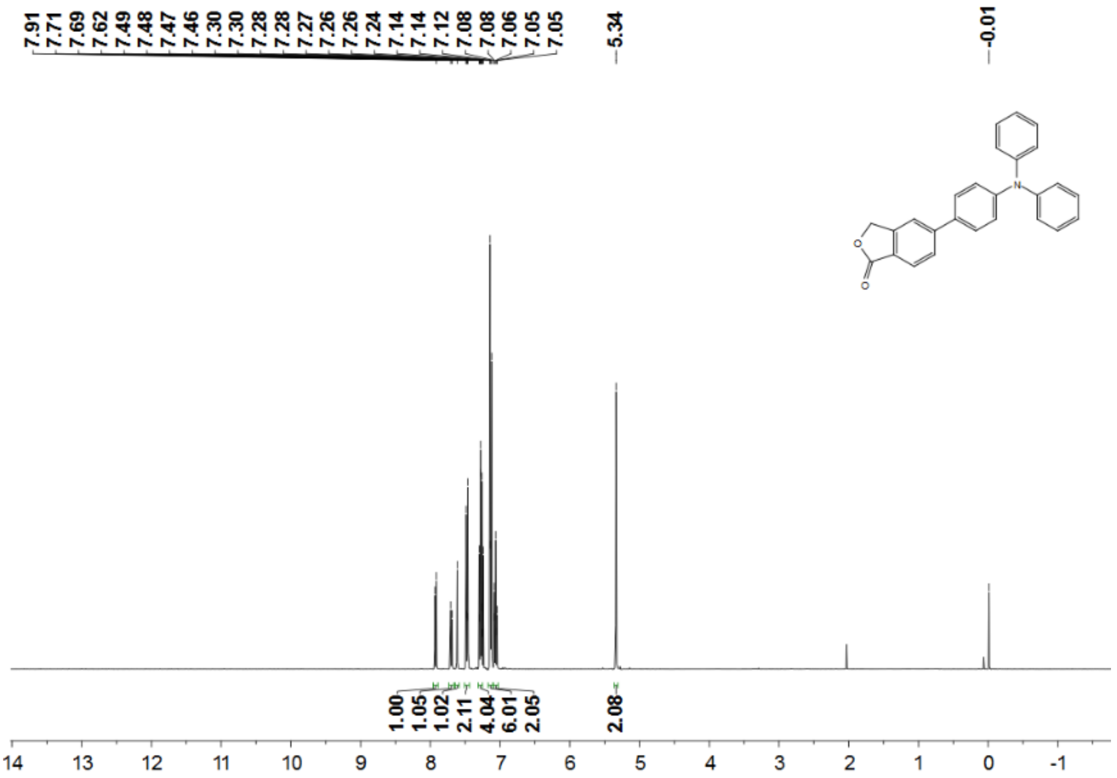
7.91 7.71 7.69 7.62 7.49 7.48 7.47 7.46 7.30 7.30 7.28 7.28 7.27 7.26 7.26 7.24 7.14 7.14 7.12 7.08 7.08 7.06 7.05 7.05
5.34
-0.01
1.00 1.05 1.02 2.11 4.04 6.01 2.05
2.08
N
O
O
14 13 12 11 10 9 8 7 6 5 4 3 2 1 0 -1

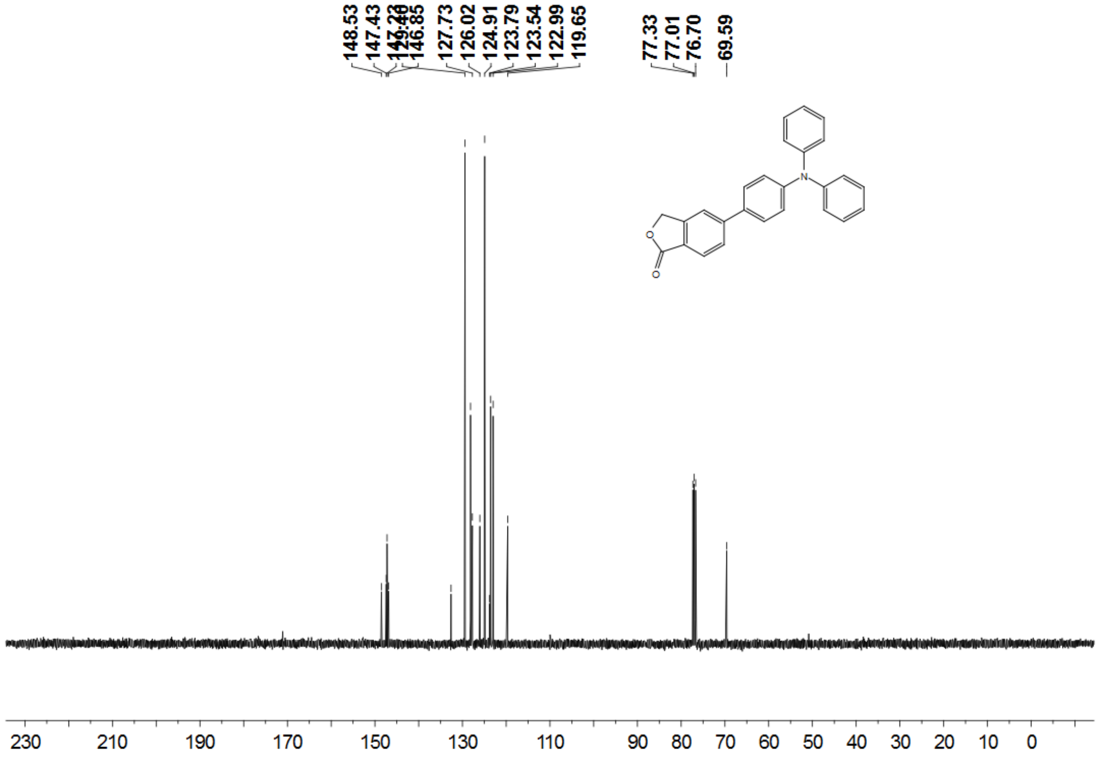
148.53 147.43 146.85 127.73 126.02 124.91 123.79 123.54 122.99 119.65
77.33 77.01 76.70
69.59
N
O
O
230 210 190 170 150 130 110 90 80 70 60 50 40 30 20 10 0

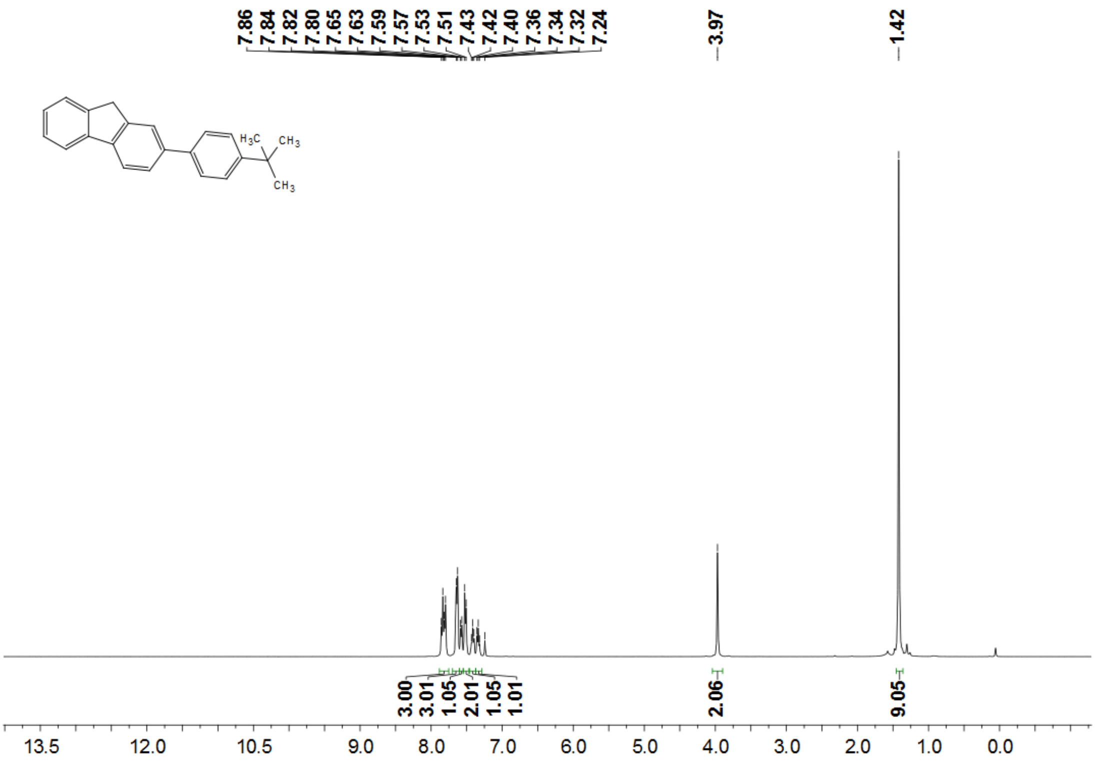
7.86
7.84
7.82
7.80
7.65
7.63
7.59
7.57
7.53
7.51
7.43
7.42
7.40
7.36
7.34
7.32
7.24
3.97
1.42
$H_3C$
$CH_3$
$CH_3$
3.00
3.01
1.05
2.01
1.05
1.01
2.06
9.05
13.5
12.0
10.5
9.0
8.0
7.0
6.0
5.0
4.0
3.0
2.0
1.0
0.0

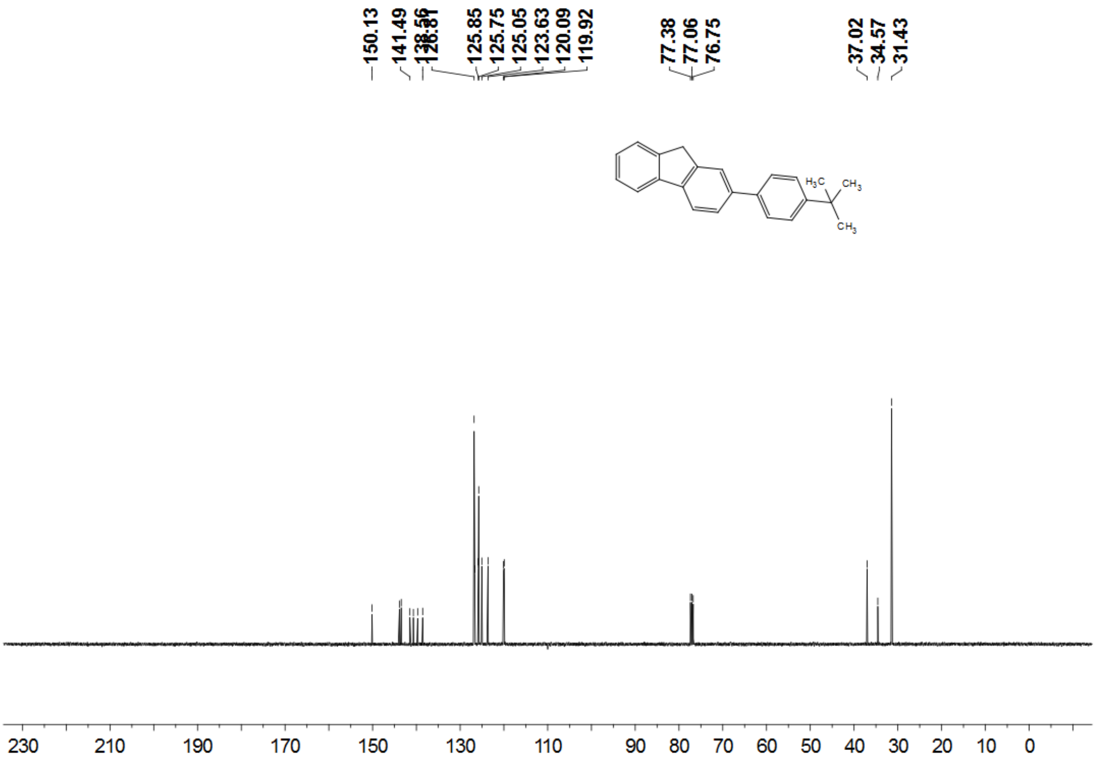
150.13
141.49
125.85
125.75
125.05
123.63
120.09
119.92
77.38
77.06
76.75
37.02
34.57
31.43
$H_3C$
$CH_3$
$CH_3$
230
210
190
170
150
130
110
90
80
70
60
50
40
30
20
10
0

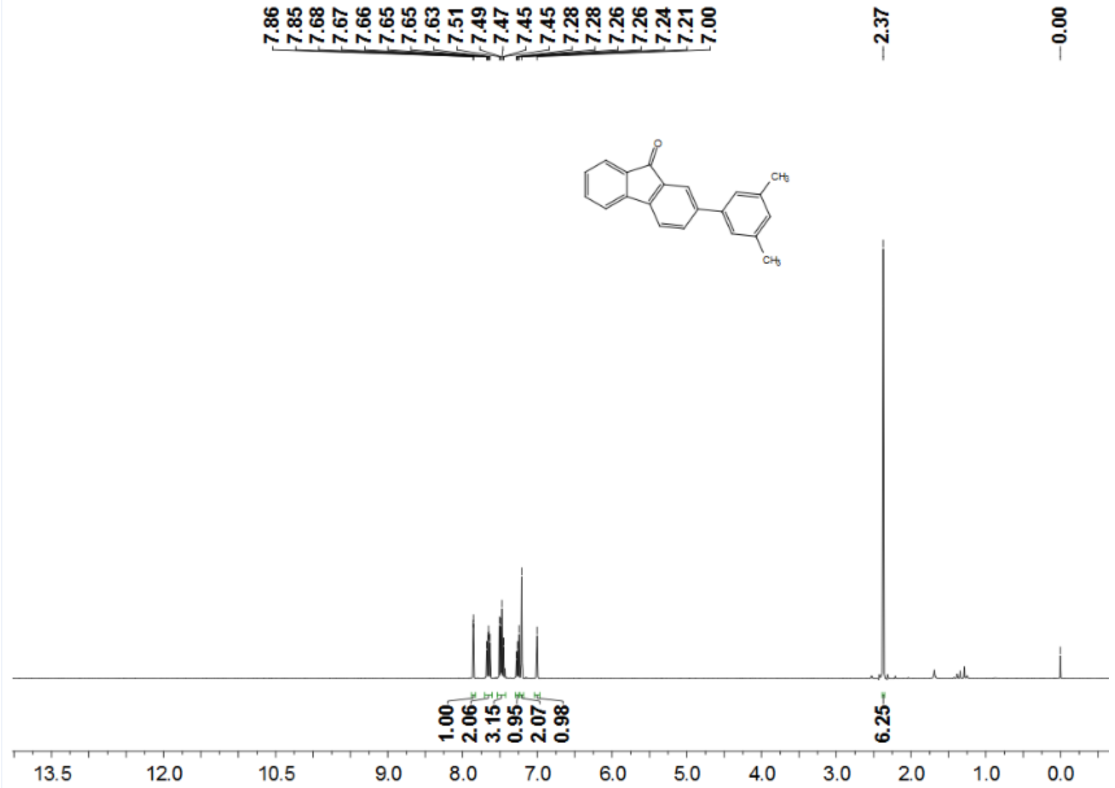
7.86 7.85 7.68 7.67 7.66 7.65 7.65 7.63 7.51 7.49 7.47 7.45 7.45 7.28 7.28 7.26 7.26 7.24 7.21 7.00
2.37
0.00
CH3
CH3
1.00 2.06 3.15 0.95 2.07 0.98
6.25
13.5 12.0 10.5 9.0 8.0 7.0 6.0 5.0 4.0 3.0 2.0 1.0 0.0

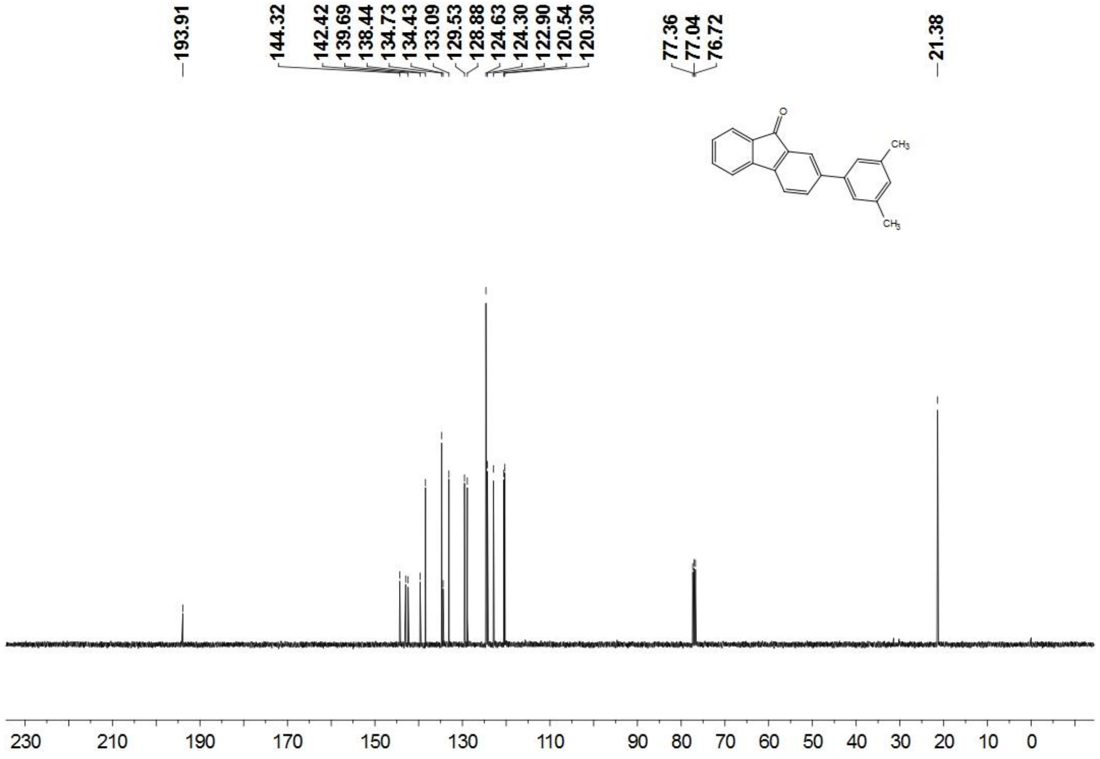
193.91
144.32 142.42 139.69 138.44 134.73 134.43 133.09 129.53 128.88 124.63 124.30 122.90 120.54 120.30
77.36 77.04 76.72
21.38
CH3
CH3
230 210 190 170 150 130 110 90 80 70 60 50 40 30 20 10 0

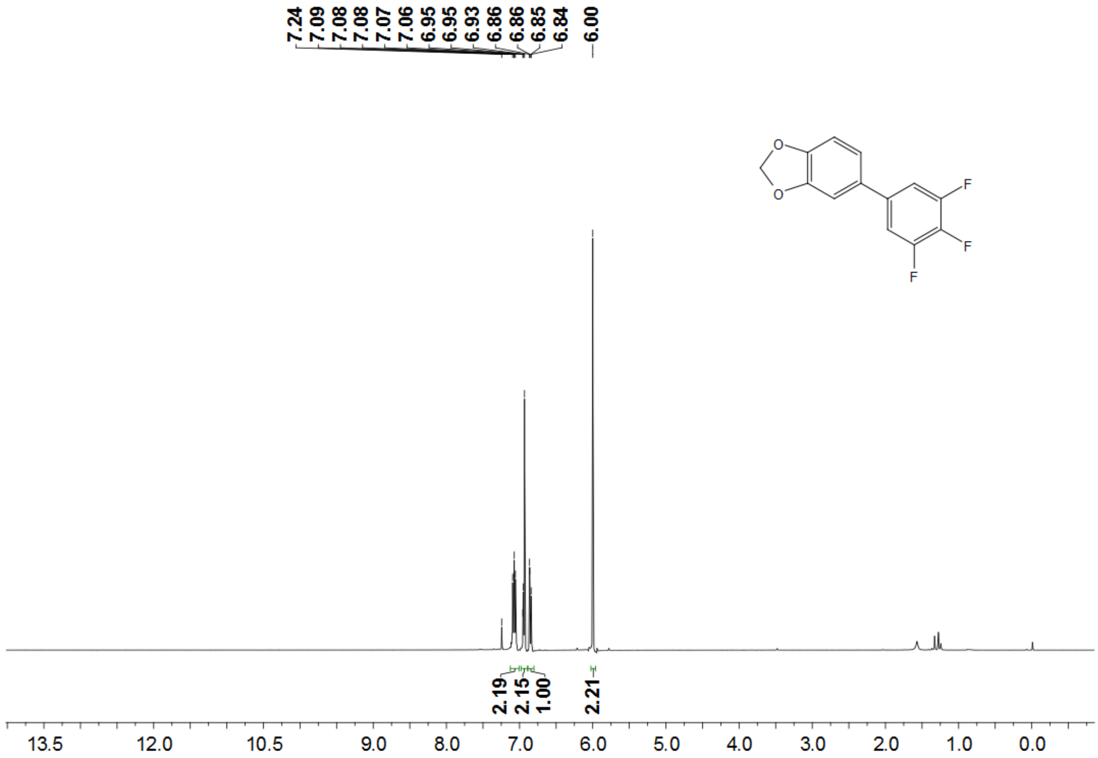
7.24
7.09
7.08
7.08
7.07
7.06
6.95
6.95
6.93
6.86
6.86
6.85
6.84
6.00
O
O
F
F
F
2.19
2.15
1.00
2.21
13.5
12.0
10.5
9.0
8.0
7.0
6.0
5.0
4.0
3.0
2.0
1.0
0.0

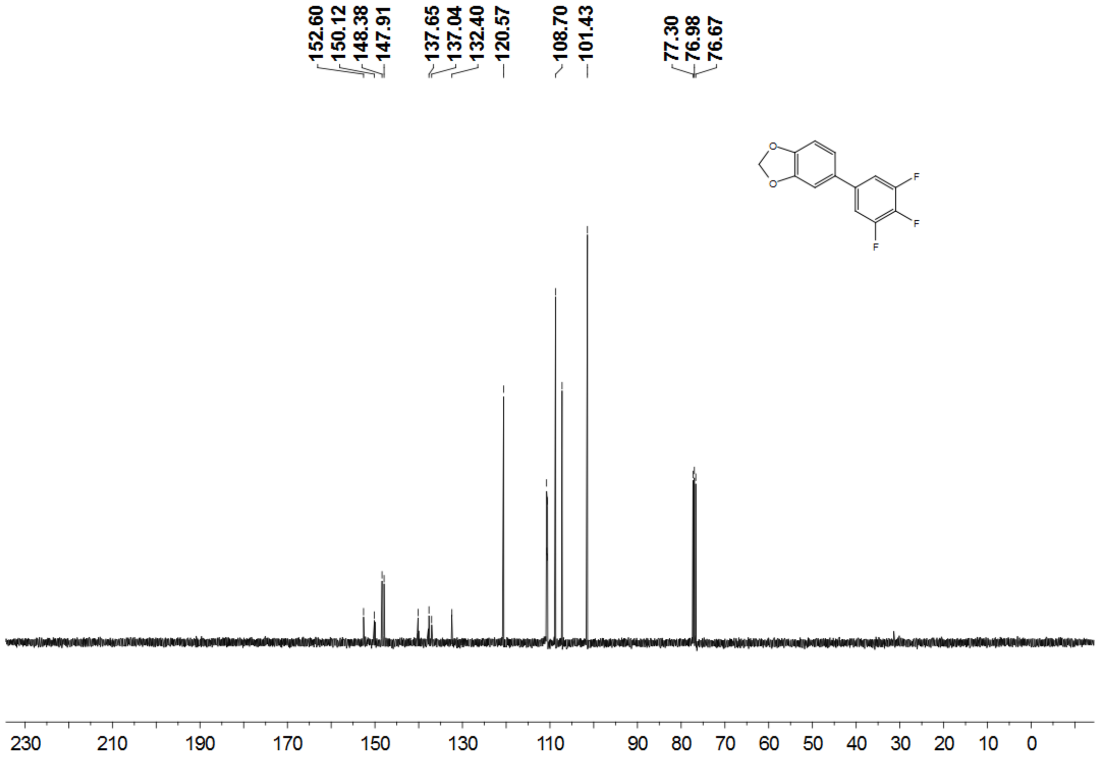
152.60
150.12
148.38
147.91
137.65
137.04
132.40
120.57
108.70
101.43
77.30
76.98
76.67
O
O
F
F
F
230
210
190
170
150
130
110
90
80
70
60
50
40
30
20
10
0

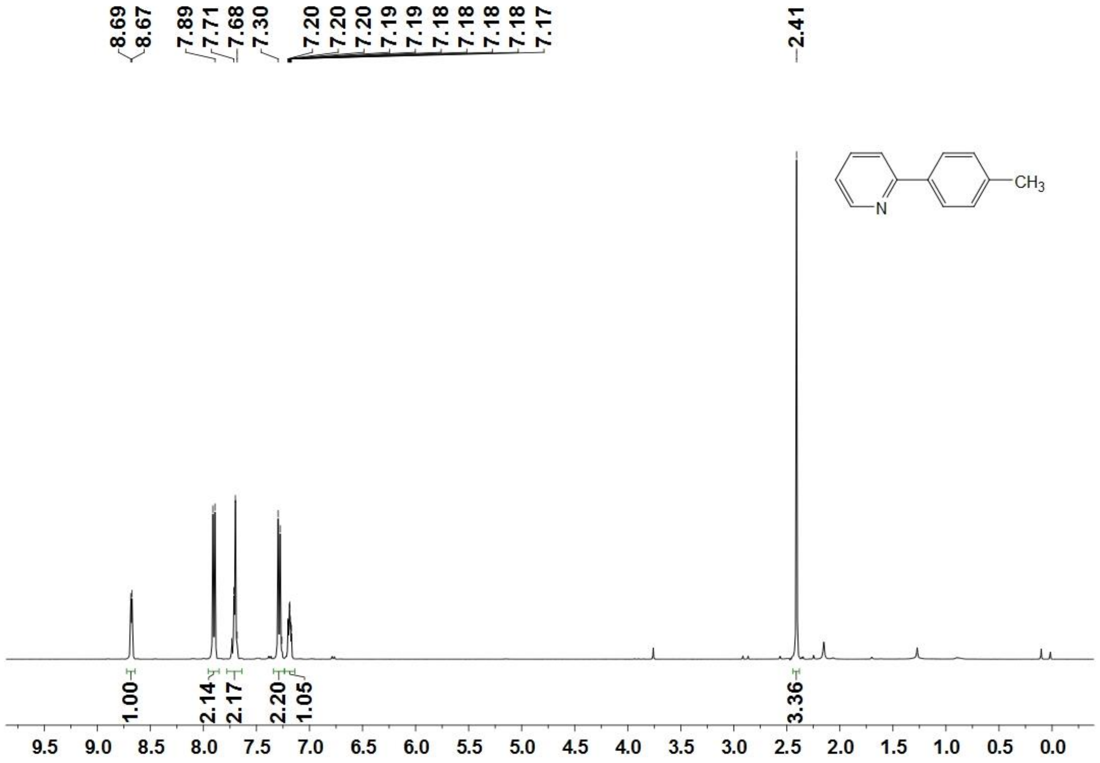
8.69
8.67
7.89
7.71
7.68
7.30
7.20
7.20
7.20
7.19
7.19
7.18
7.18
7.18
7.18
7.17
2.41
N
CH3
1.00
2.14
2.17
2.20
1.05
3.36
9.5 9.0 8.5 8.0 7.5 7.0 6.5 6.0 5.5 5.0 4.5 4.0 3.5 3.0 2.5 2.0 1.5 1.0 0.5 0.0

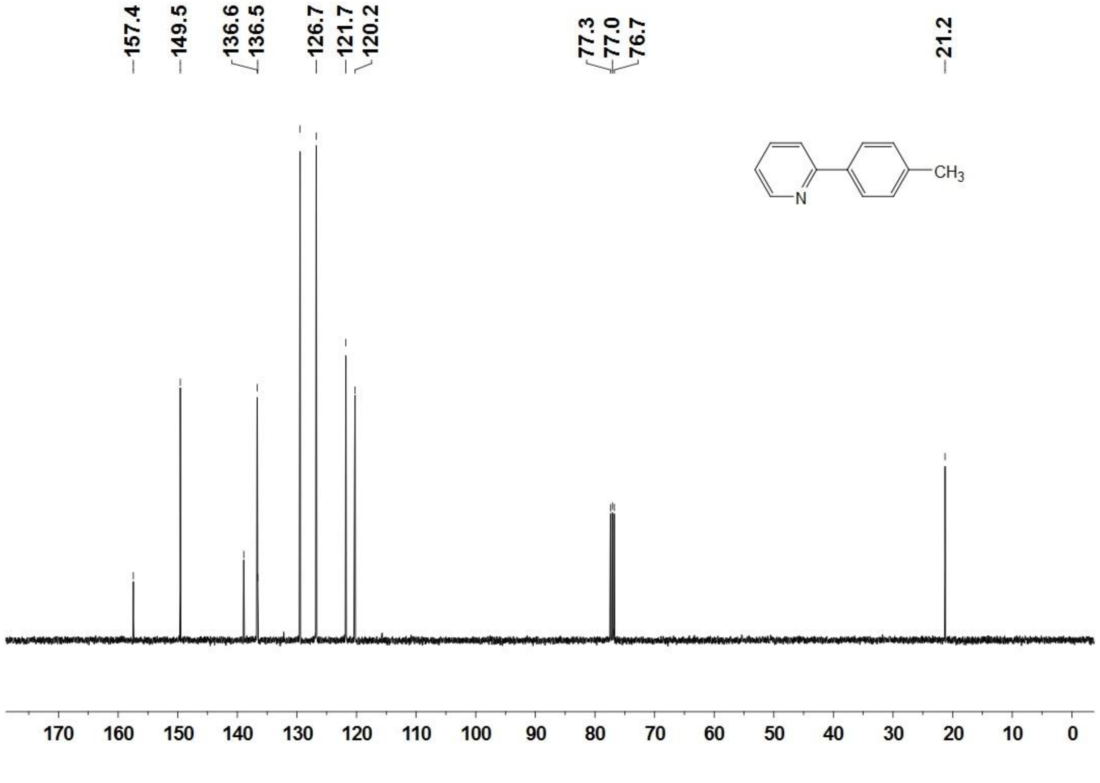
157.4
149.5
136.6
136.5
126.7
121.7
120.2
77.3
77.0
76.7
21.2
N
CH3
170 160 150 140 130 120 110 100 90 80 70 60 50 40 30 20 10 0

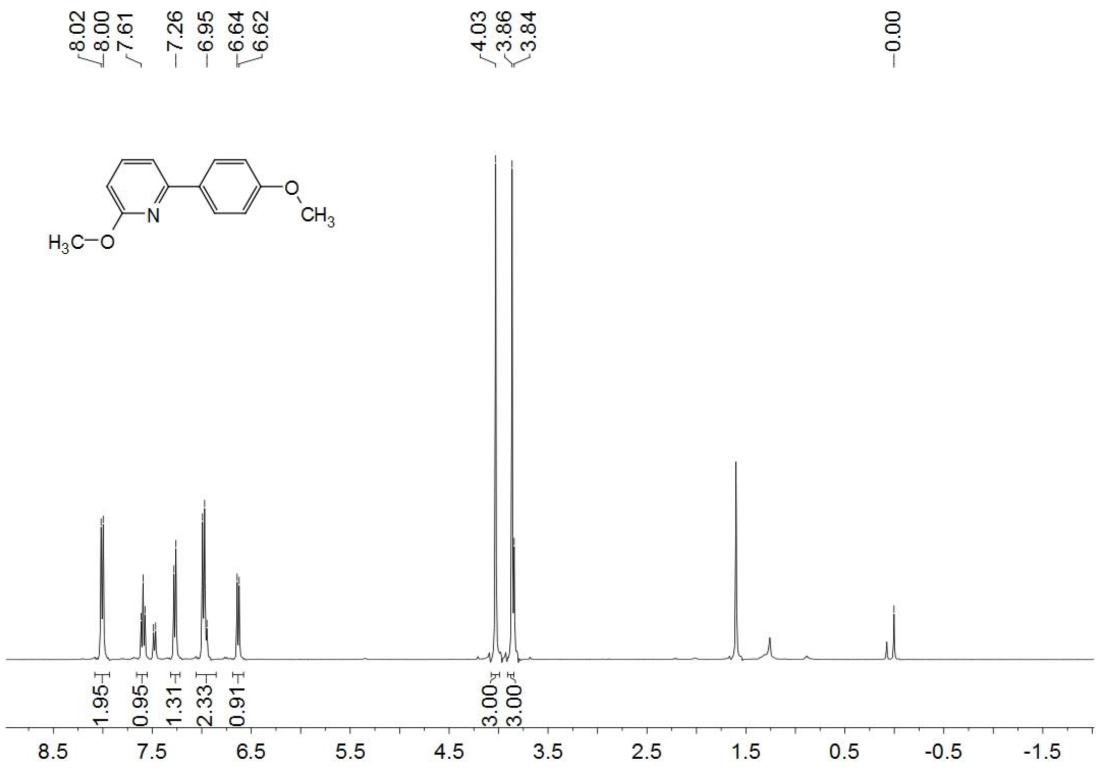
8.02
8.00
7.61
7.26
6.95
6.64
6.62
4.03
3.86
3.84
0.00
H3C−O
N
O
CH3
1.95
0.95
1.31
2.33
0.91
3.00
3.00
8.5
7.5
6.5
5.5
4.5
3.5
2.5
1.5
0.5
-0.5
-1.5

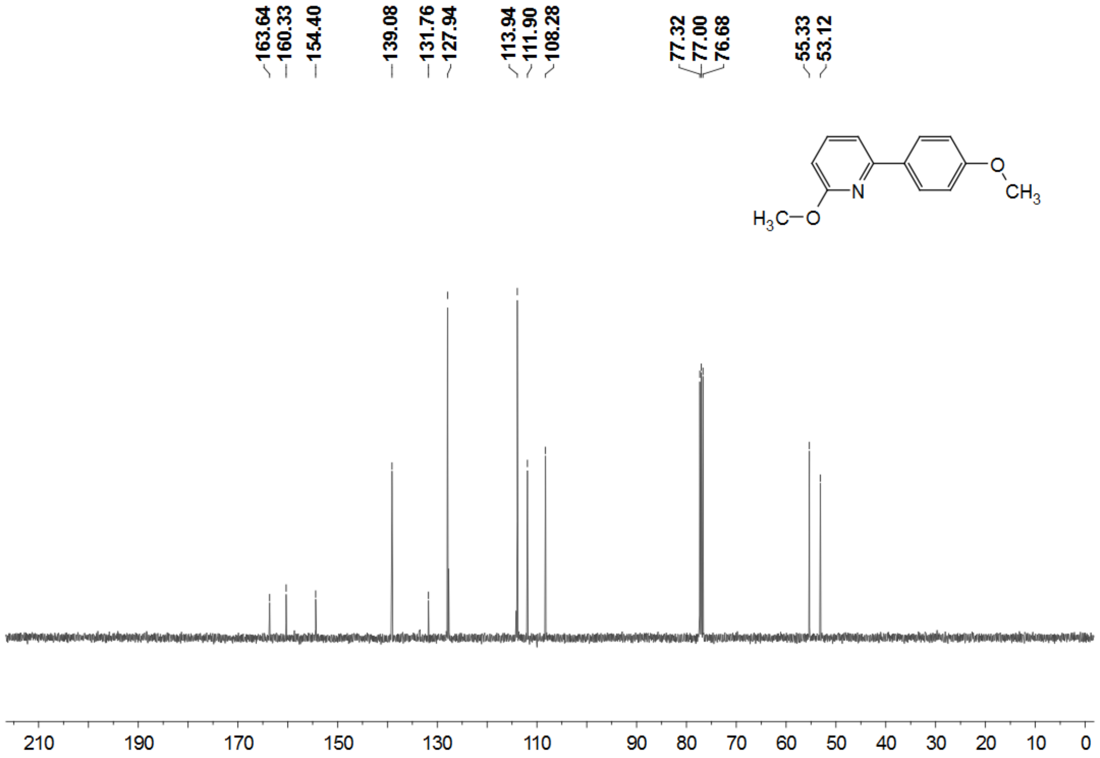
163.64
160.33
154.40
139.08
131.76
127.94
113.94
111.90
108.28
77.32
77.00
76.68
55.33
53.12
H3C−O
N
O
CH3
210
190
170
150
130
110
90
80
70
60
50
40
30
20
10
0

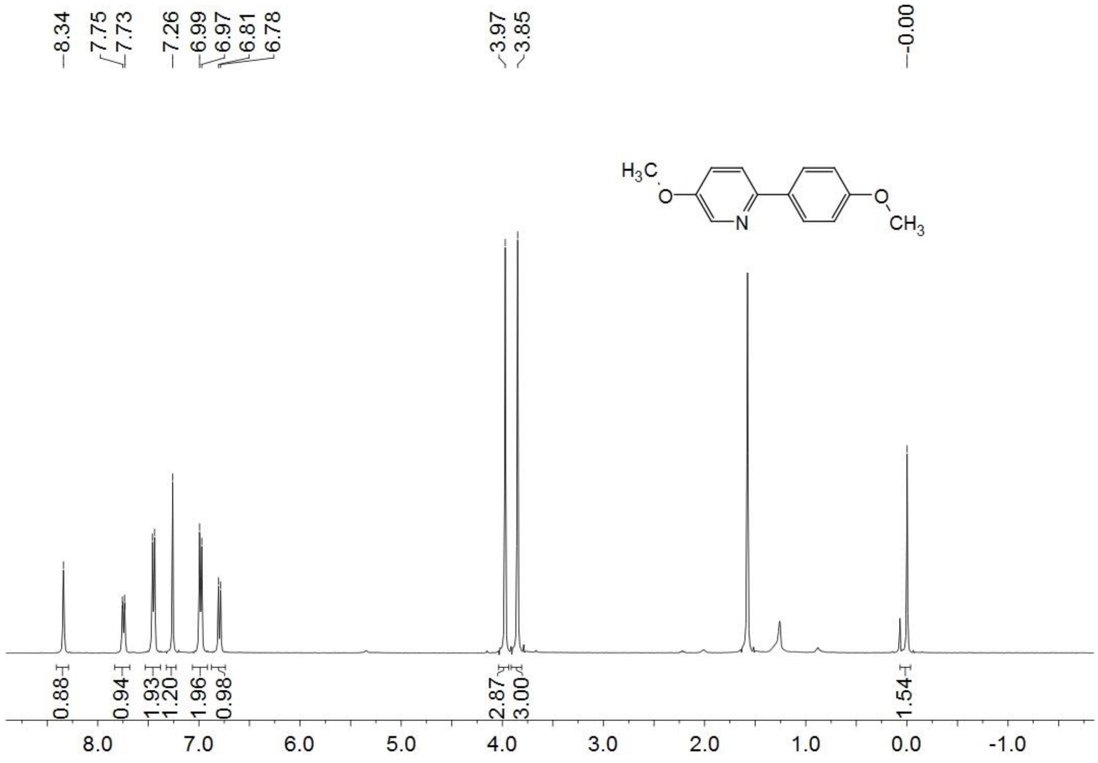
8.34
7.75
7.73
7.26
6.99
6.97
6.81
6.78
3.97
3.85
0.00
H3C
O
N
O
CH3
0.88
0.94
1.93
1.20
1.96
0.98
2.87
3.00
1.54
8.0
7.0
6.0
5.0
4.0
3.0
2.0
1.0
0.0
-1.0

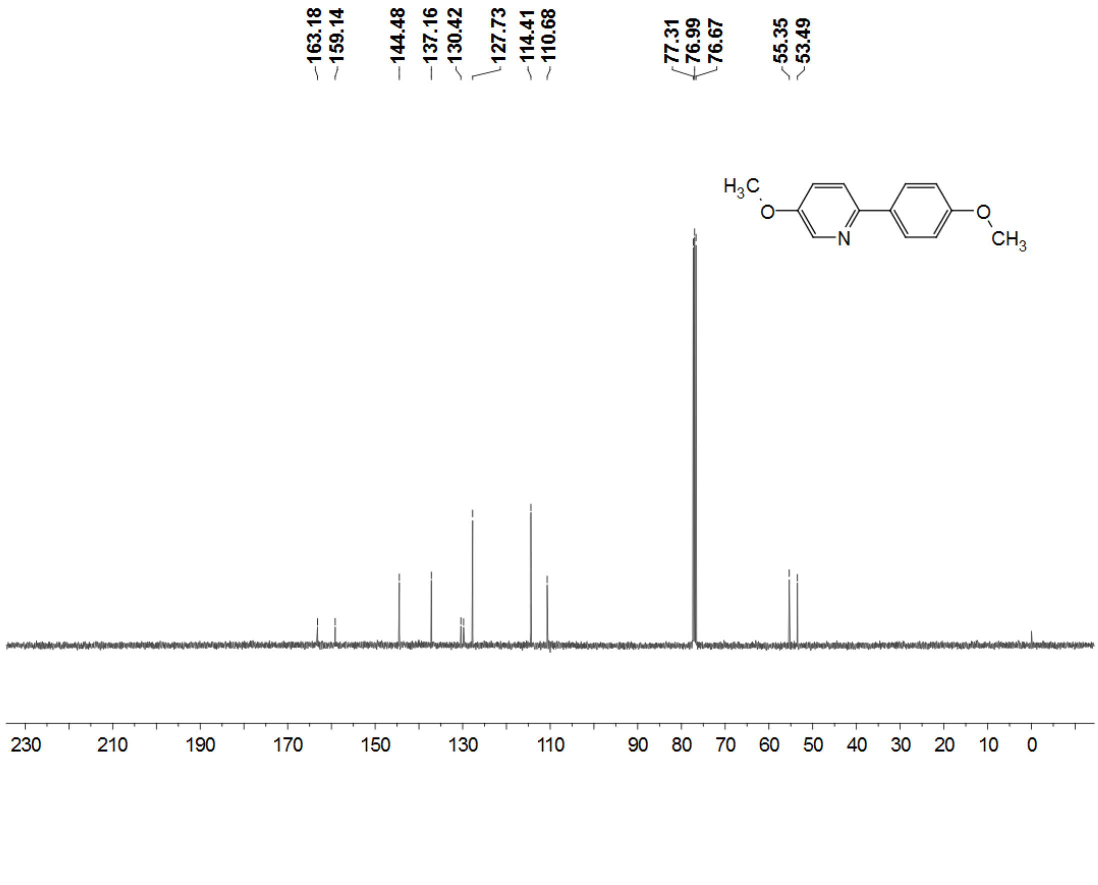
163.18
159.14
144.48
137.16
130.42
127.73
114.41
110.68
77.31
76.99
76.67
55.35
53.49
H3C
O
N
O
CH3
230
210
190
170
150
130
110
90
80
70
60
50
40
30
20
10
0

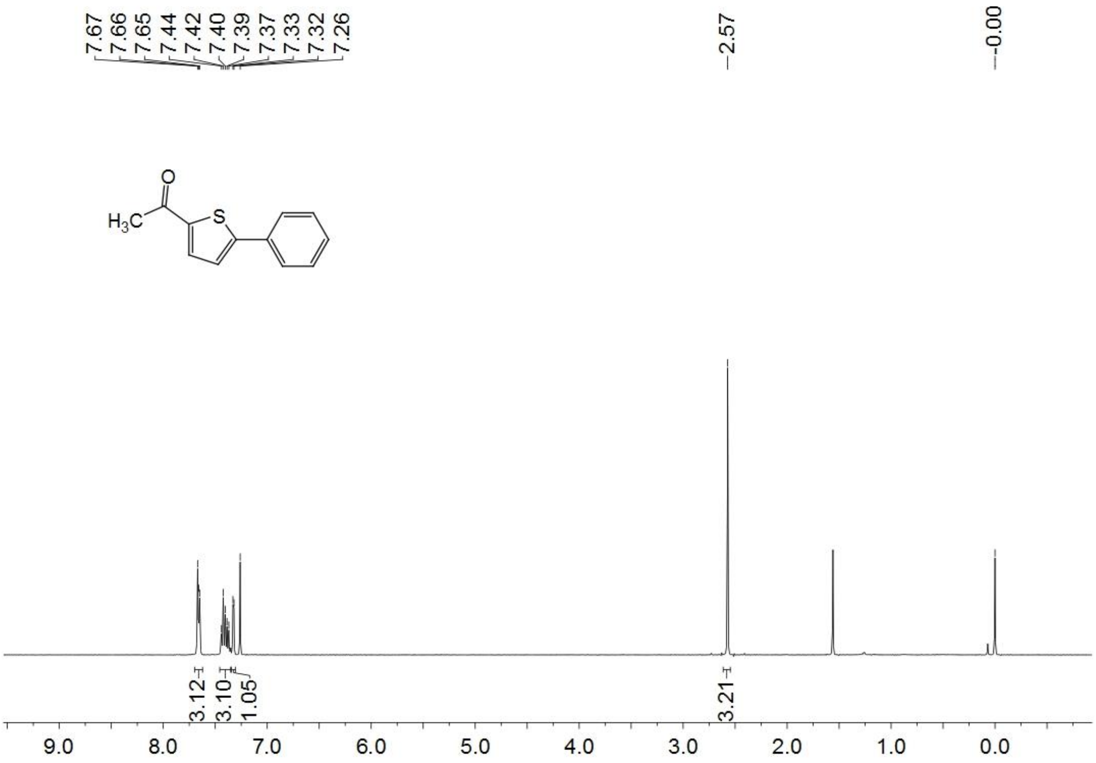
7.67
7.66
7.65
7.44
7.42
7.40
7.39
7.37
7.33
7.32
7.26
2.57
0.00
O
$H_3C$
S
3.12
3.10
1.05
3.21
9.0 8.0 7.0 6.0 5.0 4.0 3.0 2.0 1.0 0.0

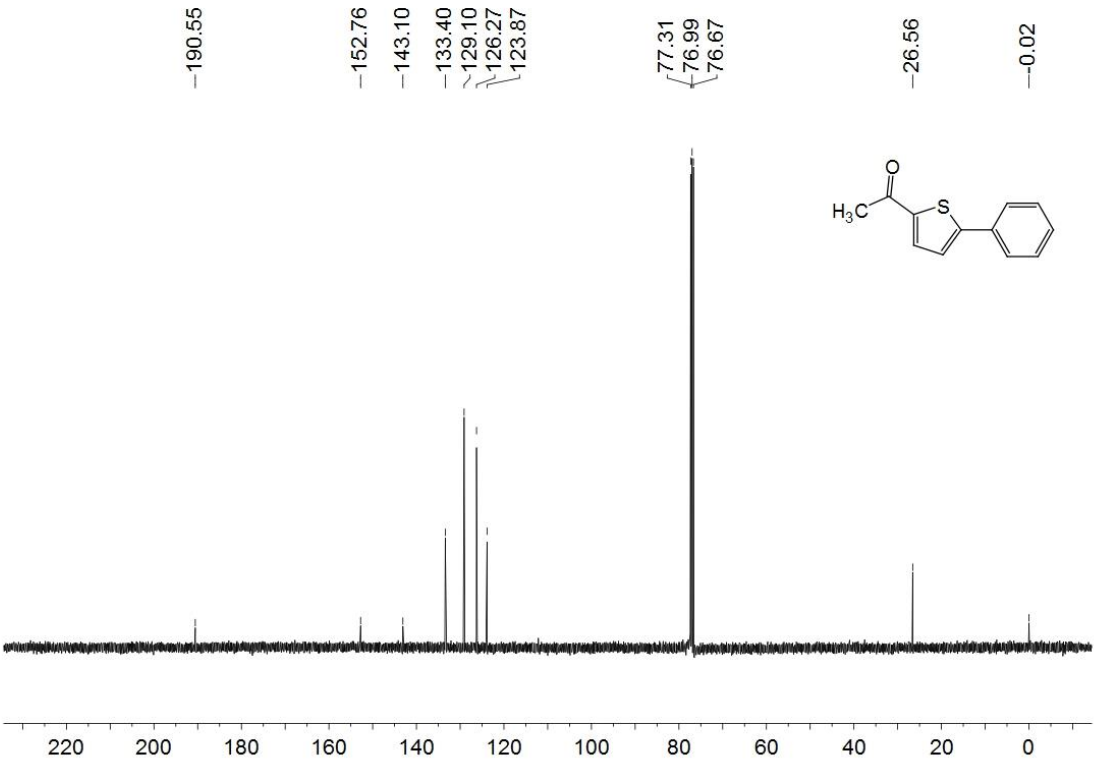
190.55
152.76
143.10
133.40
129.10
126.27
123.87
77.31
76.99
76.67
26.56
0.02
O
$H_3C$
S
220 200 180 160 140 120 100 80 60 40 20 0

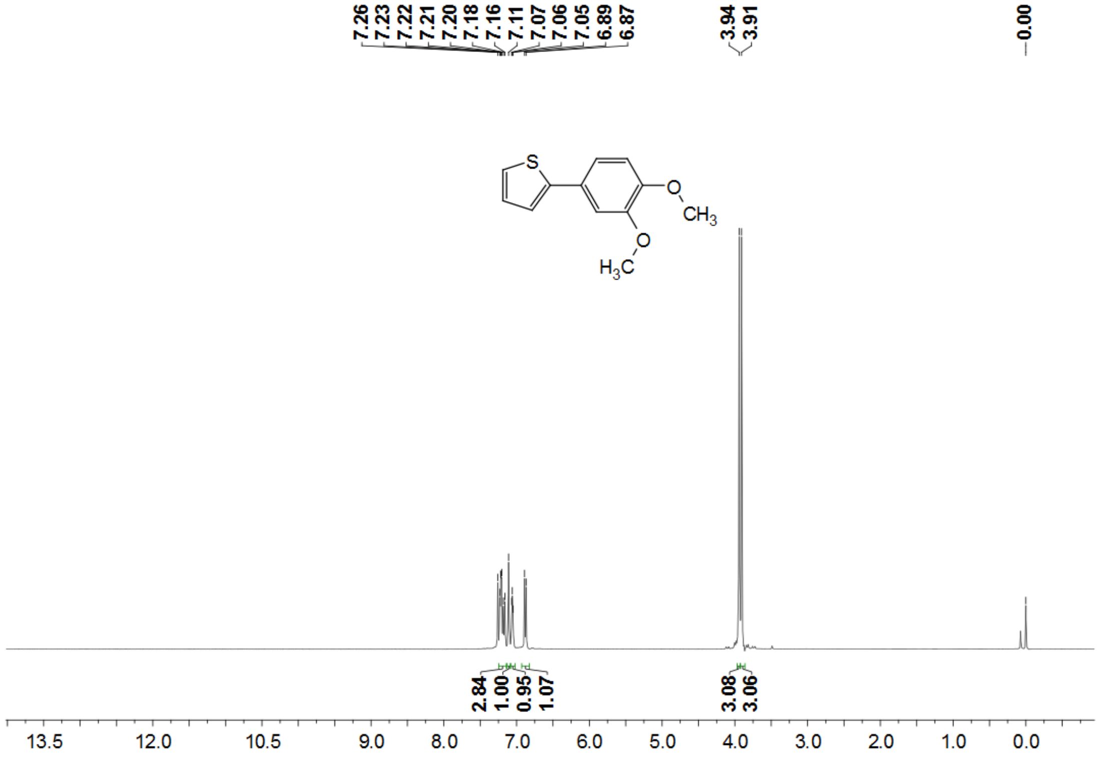
7.26
7.23
7.22
7.21
7.20
7.18
7.16
7.11
7.07
7.06
7.05
6.89
6.87
3.94
3.91
0.00
S
O
CH3
O
H3C
2.84
1.00
0.95
1.07
3.08
3.06
13.5 12.0 10.5 9.0 8.0 7.0 6.0 5.0 4.0 3.0 2.0 1.0 0.0

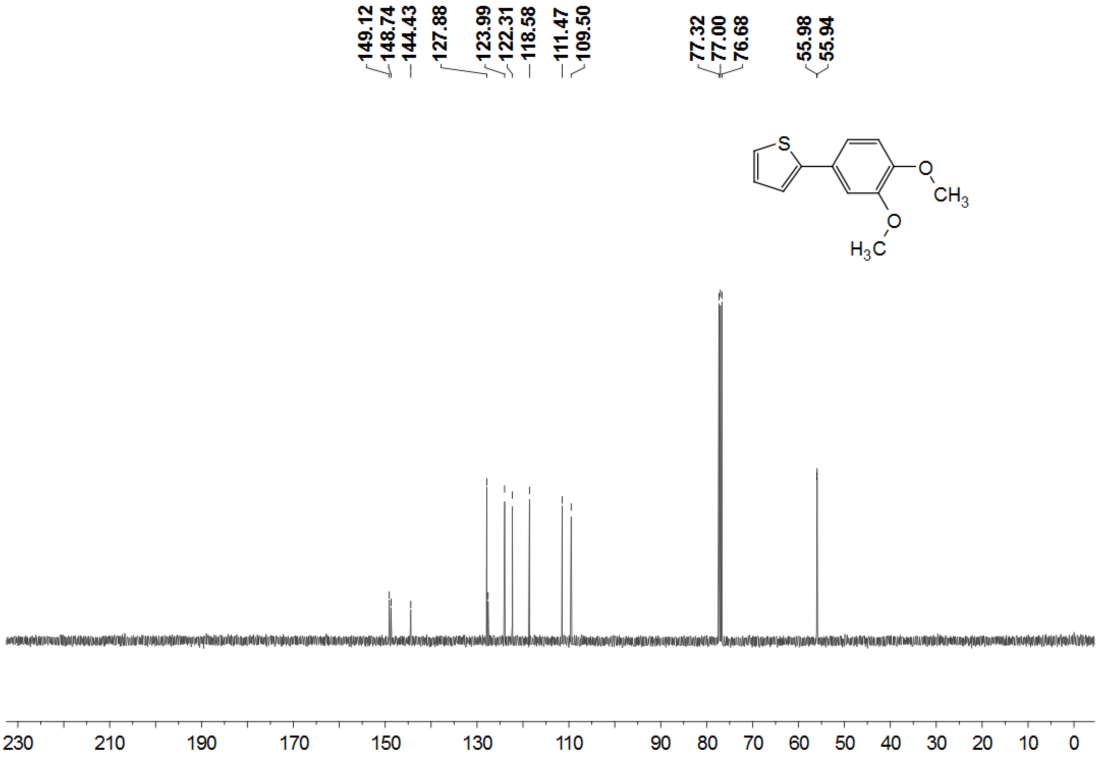
149.12
148.74
144.43
127.88
123.99
122.31
118.58
111.47
109.50
77.32
77.00
76.68
55.98
55.94
S
O
CH3
O
H3C
230 210 190 170 150 130 110 90 80 70 60 50 40 30 20 10 0

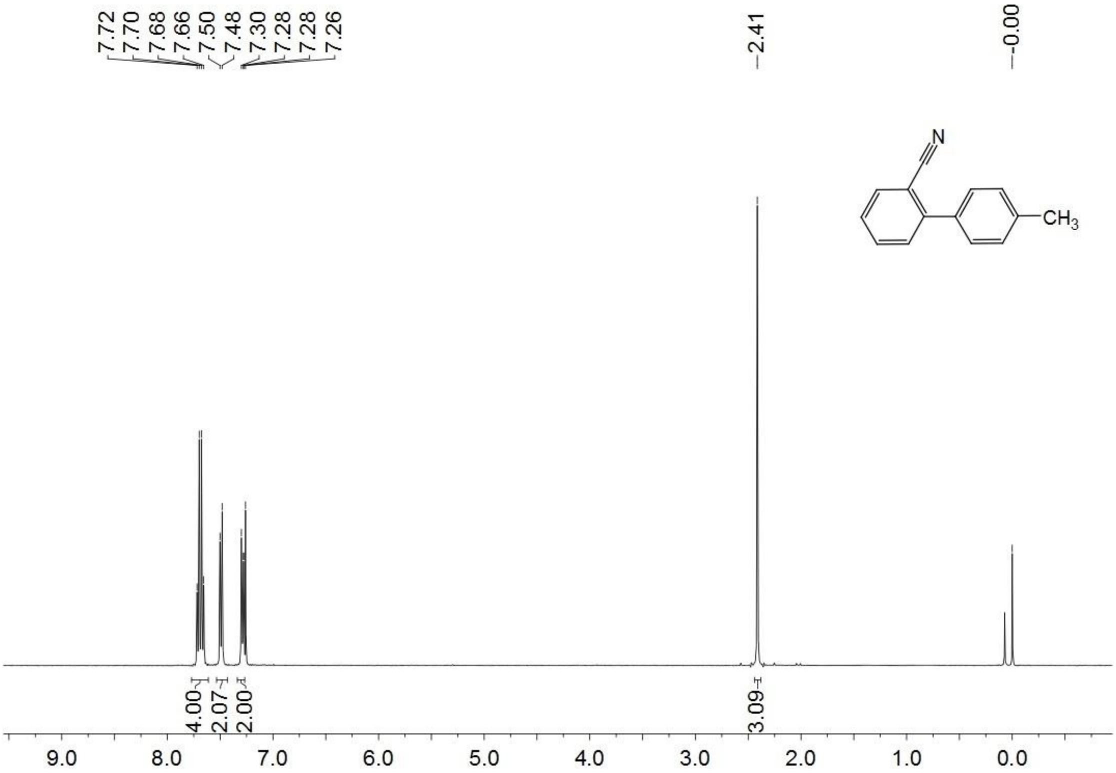
7.72
7.70
7.68
7.66
7.50
7.48
7.30
7.28
7.28
7.26
2.41
0.00
N
CH3
4.00
2.07
2.00
3.09
9.0
8.0
7.0
6.0
5.0
4.0
3.0
2.0
1.0
0.0

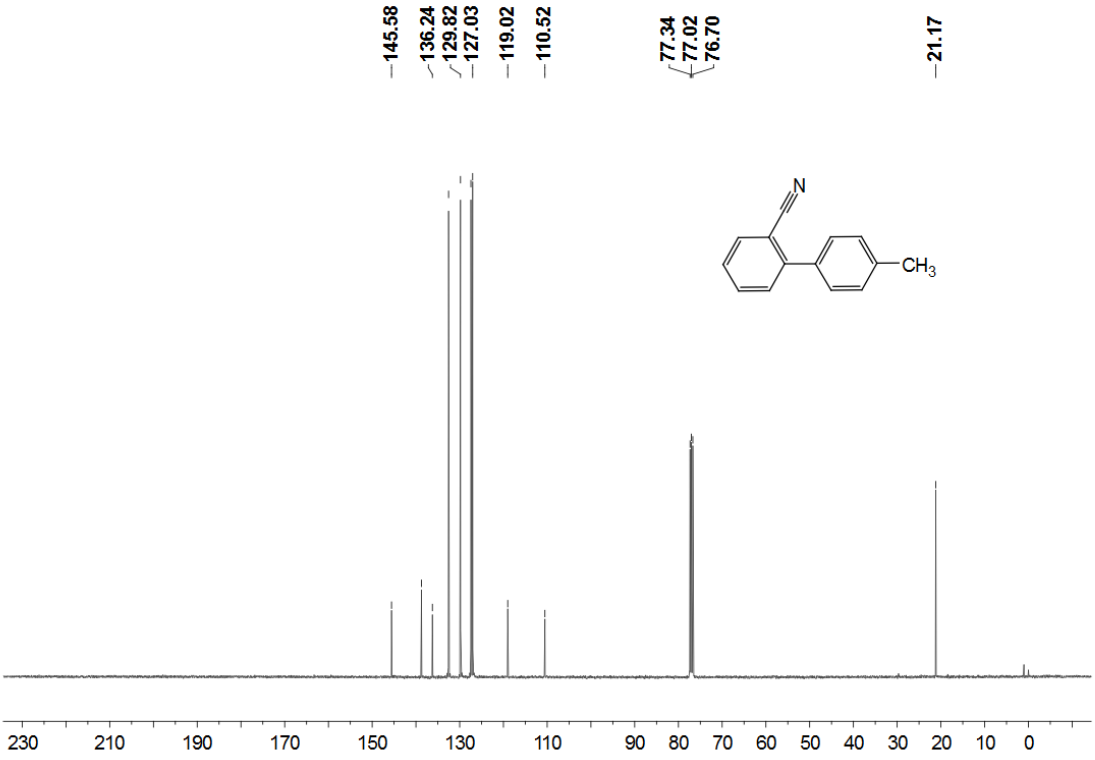
145.58
136.24
129.82
127.03
119.02
110.52
77.34
77.02
76.70
21.17
N
CH3
230
210
190
170
150
130
110
90
80
70
60
50
40
30
20
10
0
